# The burden of illness in Prader-Willi syndrome: a systematic literature review

**DOI:** 10.1186/s13023-025-03787-0

**Published:** 2025-07-24

**Authors:** Dairine Dempsey, Maria Hall, Ben Lanning, Ben Barron-Millar, Michael Huang, Neil Cowen, Mitch Nagao, Raj Gandhi, Anish Bhatnagar

**Affiliations:** 1 Soleno Therapeutics Europe Ltd, Dublin, Ireland; 2 Maria Hall Consulting Ltd, Thame, England UK; 3 Kintiga, Cambridge, England UK; 4 Soleno Therapeutics, Inc, Redwood City, CA 94065 USA

**Keywords:** Burden of illness, Direct costs, Indirect costs, Caregiver burden, Health-related quality of life, Humanistic burden, Mortality, Prader-Willi syndrome, Hyperphagia

## Abstract

**Background:**

Prader-Willi syndrome (PWS) is a rare, genetic neurobehavioral and metabolic disorder marked by hyperphagia, behavioral challenges, and significant comorbidities, requiring a multidisciplinary approach for effective management. This systematic review aimed to comprehensively evaluate the burden of disease associated with PWS, focusing on mortality, healthcare resource utilization, economic burden, and quality of life.

**Methods:**

The literature search, conducted on August 13, 2024, included the MEDLINE, Embase, and Cochrane Library databases, as well as conference proceedings. Original studies published since 2014 were selected based on relevance to PWS patient burden, covering mortality, humanistic and economic impacts. Data from the selected studies were extracted, and currency conversions were standardized.

**Results:**

For the topics of mortality, humanistic burden and economic burden, a total of 11 studies, 95 studies, and 33 studies were included, respectively. Individuals with PWS faced significantly reduced life expectancy compared to the general population, with leading causes of death including respiratory failure, consequences of uncontrolled hyperphagia, and cardiovascular complications. Hyperphagia contributed substantially to the disease burden, necessitating constant food security measures to prevent life-threatening complications. Primary caregivers, predominantly parents of individuals with PWS, experienced significant emotional and psychological strain. The time-intensive responsibilities of implementing food security measures heavily impacted their daily lives, social and family dynamics, as well as their financial health. Quality of life for patients was less frequently reported but markedly impaired, driven by physical health challenges, behavioral issues, and social isolation. Wider family dynamics were also often impacted, with siblings reporting increased psychosocial stress and feelings of neglect. The direct costs of managing PWS, including frequent hospitalizations and specialized care, were consistently reported to exceed those of matched controls without PWS, highlighting the substantial economic burden associated with the condition.

**Conclusion:**

This systematic literature review highlights the profound burden of PWS on patients, caregivers, payers of care, and healthcare systems. Complications of PWS reduce life expectancy, impair quality of life, and impose considerable financial strain. The findings underscore an urgent need for comprehensive support and innovative treatments that address the complex manifestations and consequences of PWS, particularly hyperphagia, to improve outcomes for patients and their families.

**Supplementary Information:**

The online version contains supplementary material available at 10.1186/s13023-025-03787-0.

## Background

PWS is a rare, complex and progressive neurobehavioral-metabolic disorder resulting from genetic alterations on chromosome 15q11.2-q13 [1–4]. Globally, the prevalence of PWS is estimated at approximately 1 in 10,000 to 30,000 individuals, with no substantial differences between sexes or ethnic groups [5, 6].

Neonates and infants with PWS struggle to thrive due to severe hypotonia, poor appetite, and feeding difficulties [1]. Between ages 2 and 8, children with PWS begin to prominently exhibit the lifelong hallmark feature of the syndrome: hyperphagia. Hyperphagia is a chronic and life-threatening condition marked by a persistent feeling of hunger, lack of satiety and intrusive food-seeking behavior [3]. With no pharmacological interventions to treat hyperphagia related to PWS, caregiver-implemented food security measures are the only management option available. Such measures involve locked refrigerators and pantries, keeping food items out of sight, and constant supervision of the affected individual [7, 8].

Other characteristics of PWS include behavioral complications, cognitive disabilities, and abnormal body composition [3, 8]. The behavioral profile in PWS includes aggression, temper outbursts, stubbornness, manipulative behavior, skin picking, and obsessive–compulsive behaviors, with patients at higher risk of developing psychiatric illnesses including psychosis, and mood and anxiety disorders [8]. Additionally, endocrine dysfunction, if not treated with hormone replacement therapy leads to short stature with small hands and feet and incomplete sexual development [3].

PWS is further complicated by a high prevalence of comorbidities, including sleep apnea, excessive daytime sleepiness, osteoporosis and osteopenia, and obesity that predisposes patients to impaired glucose tolerance and diabetes [9–11]. It is also associated with abnormal body composition, characterized by low lean muscle mass relative to body fat and lifelong hypotonia leading to low stamina and poor exercise tolerance [12–14]. Obesity commonly affects individuals with PWS [15–17], often leading to additional health issues [11]. Other common comorbidities include obstructive sleep apnea, respiratory problems, and scoliosis [18]. Mental health is also impacted, with high rates of self-injurious behaviors, anxiety, and depression [18].

While individual clinical aspects of PWS have been studied, no systematic evaluation has comprehensively assessed its burden on patients, caregivers, and healthcare systems. This systematic literature review (SLR) was conducted to identify and capture existing published evidence on the mortality, economic, and humanistic burden of disease associated with PWS. By providing a comprehensive overview of how this condition affects patients, caregivers, and healthcare systems, we aim to highlight the significant unmet needs in the management of PWS to inform future therapies and policies.

## Methods

### Literature search

We searched MEDLINE ALL (via Ovid.com), Embase (via Ovid.com), Cochrane Library (via the Wiley online platform), Database of Abstracts of Reviews of Effects (DARE), NHS Economic Evaluation Database (NHS EED), Health Technology Assessment (HTA) database (all via york.ac.uk/crd), and EconLIT (via Ovid.com). Additionally, grey literature sources outside of these main databases were manually searched, which consisted of selected conference proceedings and reference lists from relevant systematic reviews. Searches were conducted on August 13, 2024, and the full search strategy is provided in the Additional File 1.

### Eligibility criteria

This SLR focused on the mortality, economic impact and humanistic burden associated with PWS. We included or excluded studies in the SLR according to the population, intervention, comparator, outcomes, and study design (PICOS) criteria summarized in Table 1.

**Table 1:** PICOS study selection criteria

PICOS	Inclusion	Exclusion
Population	Patients with PWS and their caregivers, families, and siblings	Any other population
Intervention*	No intervention	N/A
Comparator*	No comparator	N/A
Outcomes	Mortality (limited to only from studies with the aim of investigating mortality) Humanistic burden on patients, caregivers and families (including siblings) Treatment burden of current treatment options Economic burden	Any other outcomes
Study design	Randomized controlled trials Non-randomized controlled studies Non-controlled studies	Case studies and series where N < 10 In vitro and in vivo studies Editorials Reviews Letters Comments Notes Erratum SLRs‡
Geographical location	No restriction	No restriction
Language	Publication in English language only	Publication in languages other than English
Publication date	Within the last 10 years (2014 to 2024)	Published prior to 2014

*N/A* not applicable, *PICOS* population-intervention-comparator-outcomes-study design, *PWS* Prader-Willi syndrome; SLR, systematic literature review

^*^The focus of this review was on disease burden independent of interventions, but interventional studies were included to extract baseline values where reported. No interventional outcomes were extracted

^‡^SLRs were included at the abstract review stage, for handsearching of the reference lists, then excluded at full-text screening

### Study selection

Following the removal of duplicate records across the databases searched, two independent reviewers screened the identified studies based on title and abstract. Full-text copies of all potentially relevant records were then obtained and evaluated in more detail against the eligibility criteria. All assessments were undertaken by two independent reviewers, with disagreements discussed and a third reviewer involved if required to reach consensus.

### Data extraction and synthesis

Data for each included study was extracted and quality-checked, producing extraction tables specifically designed for this review. Cost summaries from various studies were converted to US dollars (USD) using a straightforward exchange from Euros (EUR) and Australian dollars (AUD) at rates of 1 AUD = 0.66 USD and 1 EUR = 1.09 USD, based on xe.com exchange rates as of November 1, 2024. Sources containing verbatim quotes from caregivers were analyzed qualitatively to identify common themes and their frequency. These themes were then visualized using an online word cloud tool (WordArt.com), with more frequent themes displayed in larger fonts.

### Quality assessment/risk of bias

Quality assessments were not conducted, as the wide variation in study designs and reported outcomes would have necessitated the use of multiple checklists, making it inappropriate to draw consistent conclusions about relative study quality.

## Results

### Search results

**Fig. 1:**
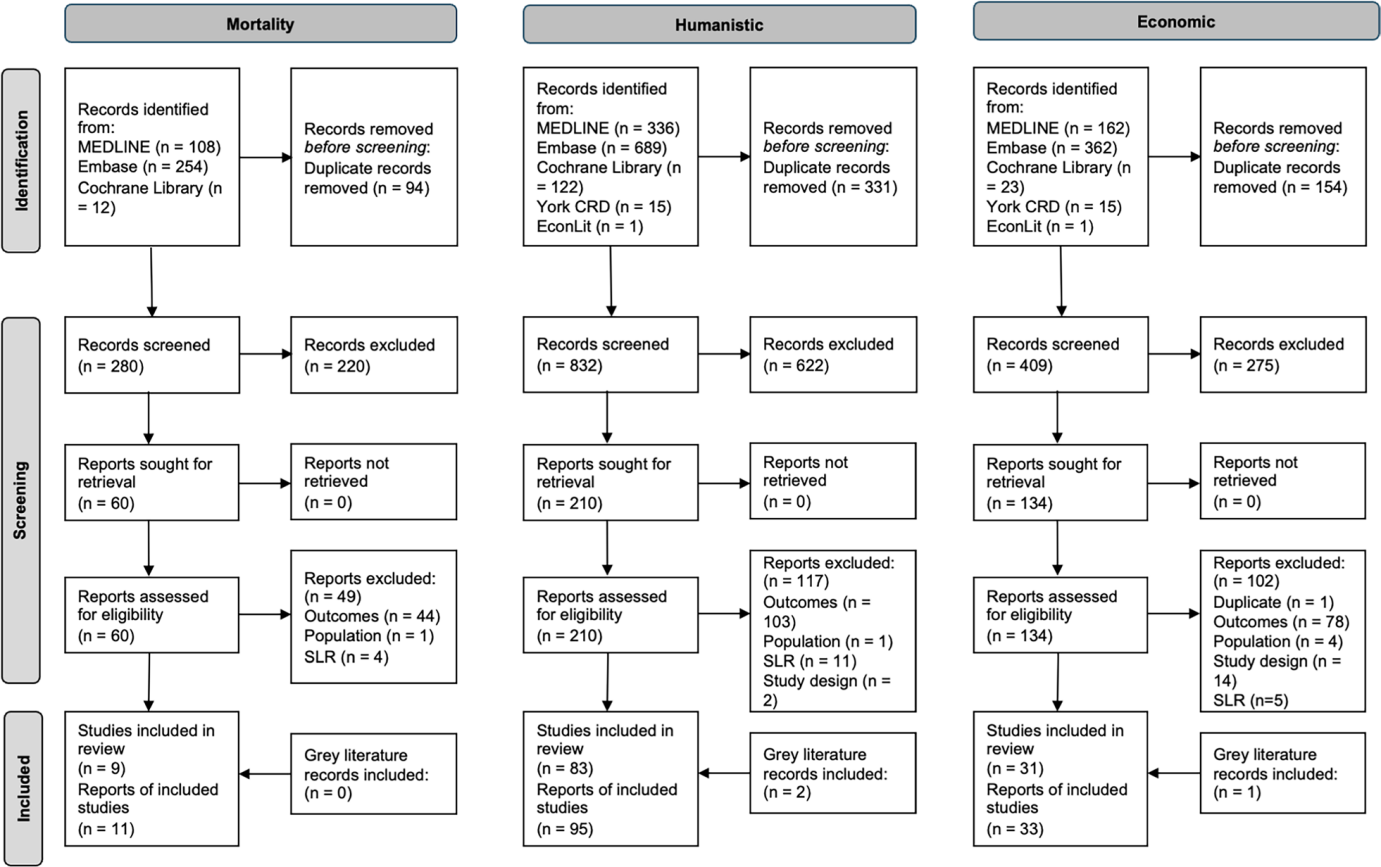
PRISMA diagram. Abbreviations: CRD, The Centre for Reviews and Dissemination; SLR, systematic literature review

For the mortality topic, a total of 374 articles were identified. After duplicate (n = 94) removal, 280 were screened according to the eligibility criteria. Following abstract screening, 60 articles were retained for full-text review, including articles identified by grey literature searches. Of these, 11 articles covering nine studies were included for narrative analysis. For the humanistic topic, from the electronic database searches, 1163 articles were identified. After duplicate (n = 331) removal, 832 were screened according to the eligibility criteria. Following abstract screening, 212 articles were retained for full-text review, including articles identified by grey literature searches. Of these, 95 articles encompassing 83 studies were included for narrative analysis. For the economic topic, from the electronic database searches, 563 articles were identified. After duplicate (n = 154) removal, 409 were screened according to the eligibility criteria. Following abstract screening, 135 articles were retained for full-text review, including articles identified by grey literature searches. Of these, 33 articles on 31 studies were included for narrative analysis. Numbers of publications included through each stage of the review are shown in Fig. 1. A tabulated summary of all included studies is provided in Additional File 2: Table S5. Elimination rates for this review were high, reflecting the broad search strategy employed to ensure a complete capture of the available literature.

### Mortality

Overall, 11 articles were identified that reported on mortality in PWS in nine distinct studies [9, 19–28]. These were based in the US (n = 5), Europe (n = 3), and Australia (n = 1).

#### Risk of death

A US claims study using 2014 data estimated the mortality rate in a population of 8,870 individuals with PWS to be nearly three times higher than in non-PWS individuals (2.7% vs 0.8%) [24]. Two further studies reported on the relative risk of mortality in PWS compared with control groups, one versus general population [9] and the other comparing recent era (2000–2015) versus early era PWS individuals (1975–2000) [23]. Compared to an age and sex-matched general population sample, Danish PWS patients hospitalized between 1995 and 2012 were at greater risk of death (relative risk [RR]: 11.0 [95% confidence interval [CI]: 5.7–21.1]) with risk peaking at 30–39 years of age (RR: 27.7 [95% CI: 9.1–84.1]) [9]. Additionally, the US cohort comparing eras of PWS patients highlighted a potential trend for improvements in mortality outcomes over time with increased all-cause mortality (hazard ratio [HR]: 1.5 [95% CI: 1.2–1.9]) in early versus recent era PWS patients [23].

#### Causes of death in PWS

**Fig. 2:**
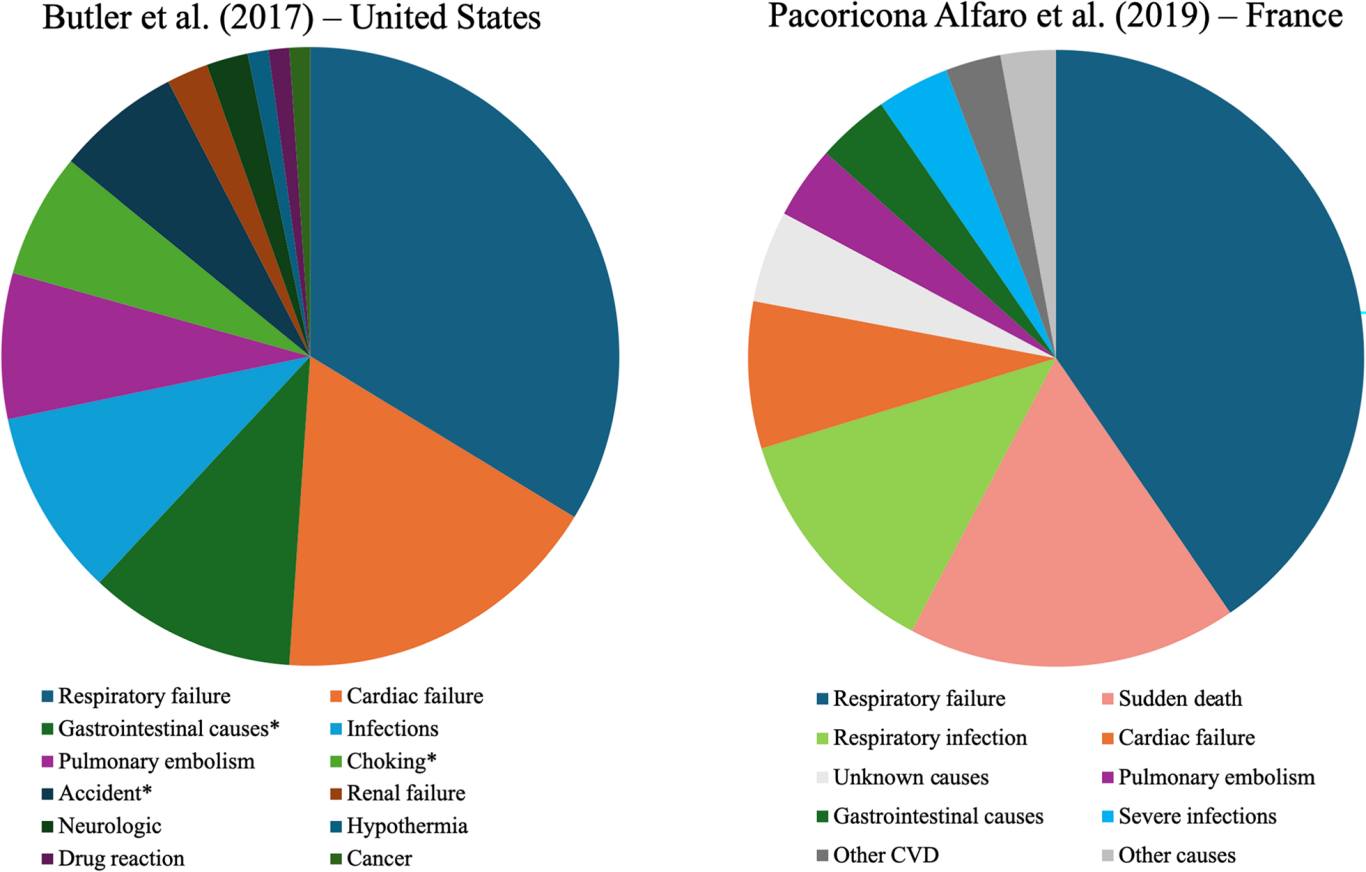
Common causes of death in PWS across two retrospective observational studies * These causes of death were presumed by Butler et al. to be related to uncontrolled hyperphagia. Abbreviations: CVD, cardiovascular disease; PWS, Prader-Willi syndrome Source: Adapted from Butler et al. [22] and Pacoricona Alfaro et al. [25]

Two studies reported in three publications specifically investigated the causes of death in PWS patients including a large US retrospective cohort study (N = 486) [22, 23] and a French retrospective, observational cohort study (N = 104) [25]. The US study reported 40 years of data from the PWS Association USA (PWSA USA) while the French retrospective observational study followed patients for 11 years [22, 25]. Across all age groups (infants, children, adolescents and adults), respiratory failure was the most common single cause of death in both studies, reported as 31.4% in the US study and 40.4% in the French study (Fig. 2) [22, 25]. In the US study, high rates of accidents, choking, and gastrointestinal problems (including perforation, distension or obstruction), likely related to uncontrolled hyperphagia and food seeking behaviors, contributed to approximately a third of all deaths and a half of deaths in patients under 18 years old [22]. Across two US surveys conducted in 2004 and 2014, of 114 reported deaths in patients with PWS, most deaths were classified by caregivers as ‘sudden’ (72.1%) or ‘unexpected’ (77.9%) [27].

#### Life expectancy

Four publications were identified that reported mortality age distribution trends in PWS [22, 24, 25, 27]. The mean mortality ages ranged from 23 to 32 years, reflecting a reduced life expectancy for PWS patients compared to the general population (Table 2).

**Table 2:** Mortality age distribution in PWS

Study references	Study type (n)	Study duration	Total number of deaths (n/N)	Mean (SD) age of death, years	Median (range) [IQR] age of death, years
Butler et al. [22]	Mortality survey database	1973 to 2015 [42 years]	425/486*	29.5 (15)	NR
Pacoricona Alfaro et al. [25]	Retrospective observational	2004 to 2014 (11 years)	104	NR	30 (0.1–58.0)
Proffitt et al. [27]	Familial-response questionnaire	2004 to 2014 (10 years)	114	31.6 (14.5)	NR
McCandless et al. [24]	Population-based study using a claims database	2012 to 2014 (2 years)	240/8,870‡	NR	23 [6–36]

*IQR* interquartile range, *NR* not reported, *PWS* Prader-Willi syndrome, *SD* standard deviation

^*^Out of these 425 deaths, 312 individuals reported a precise cause of death

^‡^PWS prevalence and mortality rates were calculated for 2014, then 2018 US census data was used to project rates for the total US population. Mortality rate of 2.7% used to estimate total number of deaths

### Humanistic burden

In this review, 95 publications reporting on 83 studies were identified that reported on the humanistic burden in PWS patients, families, or caregivers [9, 12, 15–17, 29–118].

#### Hyperphagia

Hyperphagia is a debilitating and hallmark feature of PWS associated with a chronic and life-threatening persistent feeling of hunger and lack of satiety as well as extreme food preoccupation and food-seeking behavior [8]. Despite this, no studies were identified that directly reported the impact of hyperphagia on patient QoL. However, two US time-trade off (TTO) studies in caregivers of people with PWS (N = 790) found that avoiding hyperphagia in a hypothetical health state decreased the burden of PWS [38, 71]. Quality-adjusted life years (QALYs), as per disease burden assessment of patient vignette health states, were estimated to be higher in the hypothetical health state of patients with treated hyperphagia and obesity (0.91–0.92; based on investigational therapies’ targeted product profile) versus those with treated obesity and untreated hyperphagia (0.79–0.83) or untreated PWS (0.69–0.74) [38, 71].

#### Non-food-related behaviors

**Fig. 3:**
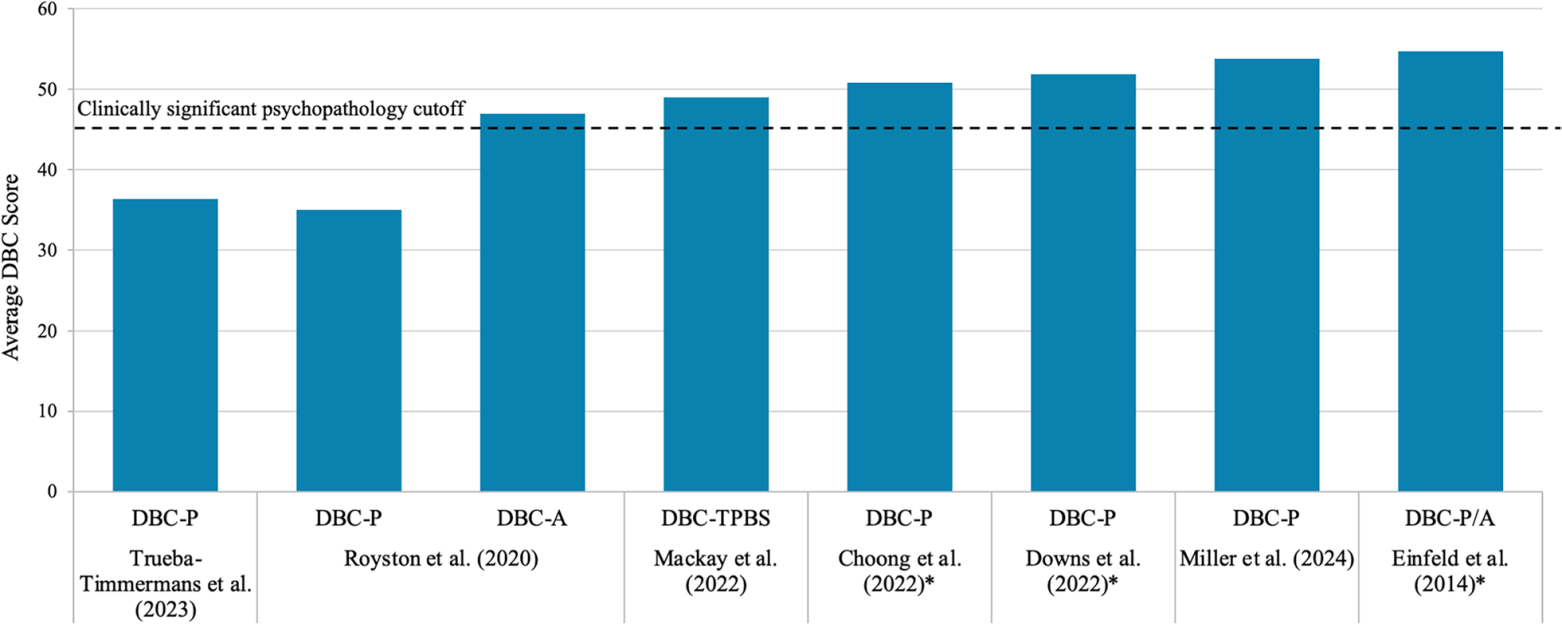
Average DBC scores from PWS cohorts of seven studies. Note: Royston et al. (2020) report median values, otherwise values are reported as means. Horizontal line depicts cutoff value for clinically significant psychopathology [119]. Values marked with * were converted from 0–2 scale (using an average of answers scored 0, 1 or 2) to 0–192 scale (using a sum of answers scored 0, 1 or 2) by the authors of this SLR by multiplying by 96 as per the methods of Taffe et al. [119]. Abbreviations: DBC-(P/A/TPBS), Developmental Behavior Checklist-(Parent/Adult/Total Problem Behavior Score); PWS, Prader-Willi syndrome. Sources: Choong et al. (2022); Downs et al. (2022); Einfeld et al. (2014); Mackay et al. (2022); Miller et al. (2024); Royston et al. (2020); Trueba-Timmermans et al. (2023) [44, 50, 53, 75, 85, 99, 108]

The Developmental Behavior Checklist (DBC) assesses behavioral and emotional problems in young people with developmental and intellectual disabilities. Eight studies assessed patients with PWS before clinical intervention using the DBC-P ([completed by] Parent/Primary Caregiver) for patients aged 4–18 years or DBC-A (Adult) for patients aged ≥ 18 years [44, 50, 53, 72, 75, 85, 99, 108], with seven reporting quantitative scores, as summarized in Fig. 3. Most of these studies found that the DBC scores for PWS cohorts were ≥ 46, the cutoff for clinically significant psychopathology [119]. No studies were identified comparing DBC scores in PWS and control populations.

The Prader-Willi Syndrome Profile (PWSP) is a PWS-specific scale that assesses behavioral and emotional issues associated with the syndrome, including aggression, anxiety, compulsivity, depression, disordered thinking, and rigidity/irritability [51, 85, 106]. Although PWSP scores were reported across three studies, none compared PWSP scores in PWS and control populations, and pathological thresholds were not identified in the literature [51, 85, 106]. Consequently, although used to measure treatment effects in clinical studies, estimating the patient burden of PWS using this instrument is not yet feasible based on the current literature.

#### Intellectual impairment and social isolation

PWS is associated with intellectual disability and patients face notable educational and professional challenges. Three studies assessed educational facility requirements, reporting that a significant proportion of their PWS cohorts (37.5% to 78.8%) required specialized educational facilities [17, 57, 103]. Furthermore, Paepegaey et al. (2018) also found that of 33 adults with PWS, most reported either no professional activity (55%) or worked in sheltered workspaces (40%) [17].

Caregivers reported that for children with PWS, daytime sleepiness resulted in missed educational opportunities [90]. Daytime sleepiness was also reported to limit social opportunities and ability to maintain peer relationships [90]. Furthermore, social events such as parties were often avoided due to hyperphagic behaviors [78]. As such, themes of struggling to make and keep friends were reported by caregivers [93, 103].

#### Health-related quality of life scales

In total, 25 different instruments were used to assess QoL across the included studies. Descriptions of these tools and a summary of their use across studies are provided in Additional File 2: Table S6.

The Pediatric Quality of Life Inventory (PedsQL), EuroQol 5 Dimension (EQ-5D) and the 36-item short form health survey (SF-36) were most often used across the identified studies to measure patient QoL. PedsQL, a QoL scale designed for healthy as well as acute and chronically ill children [120], was reported in nine publications encompassing seven studies [16, 59, 60, 81, 82, 100–102, 115]. PedsQL total scores were reported using a 0–100 scale in five of these studies and ranged from 49.0 to 70.9 for child-reported scores and 55.1 to 65.8 for parent proxy-reported scores (baseline scores taken for interventional studies) [16, 100–102, 115]. These values almost all fall below 70, which is the lowest cutoff value for identifying children with special healthcare needs [121].

EQ-5D is a QoL scale focused on five dimensions—mobility, self-care, usual activities, pain/discomfort and anxiety/depression [122], for which scores were reported in four studies. Results are reported on a 0–100 scale with 100 being full health and zero being a state as bad as death. EQ-5D scores for PWS patients in the four studies ranged from 36.6 to 81.5 [41, 42, 71, 73].

SF-36 is a QoL scale covering eight domains including physical/social/role limitations, pain, mental health, vitality, and general health [123]. Two studies reported SF-36 scores in patients with PWS. Mastey Ben-Yehuda et al., (2024) reported mean SF-36 domain scores for PWS patients living in either specialized hostels versus living either at home or in non-specialized hostels as physical functioning: 90.0 vs. 72.2, role-physical: 70.5 vs. 67.0, bodily pain: 76.7 vs. 80.2, general health: 64.4 vs. 71.0, mental health: 68.7 vs. 73.3, social functioning: 74.2 vs. 78.5, role-emotional: 66.7 vs. 82.7 and vitality: 64.5 vs. 57.8 [77]. Shriki-Tal et al., (2017) reported no numerical values but found that SF-36 scores negatively correlate with the patients’ number of psychiatric diagnoses, including anxiety, depression, and skin picking [105].

#### Family and caregiver burden

**Fig. 4:**
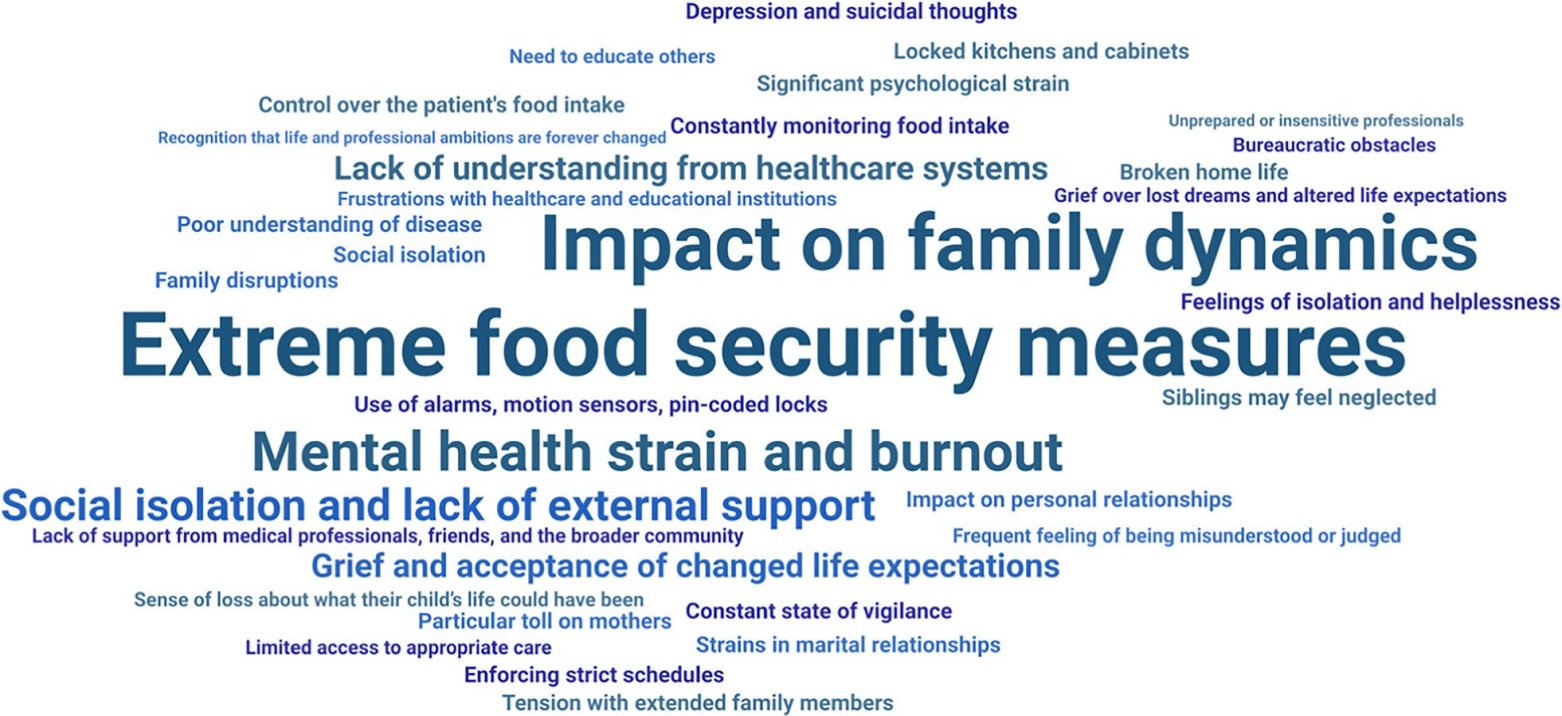
Thematic word cloud with the most common themes reported by caregivers of patients with PWS. Note: Font size denotes the frequency of key terms. Abbreviations: PWS, Prader-Willi syndrome. Sources: Created on WordArt.com using caregiver quotes from Dykens et al. (2024); Currie et al. (2024); Matesevac et al. (2023); Kowal et al. (2022); Patel et al. (2022); Schofield et al. (2021); Ragusa et al. (2020); Cagalj et al. (2018); Vitale et al. (2016); Thomson et al. (2017) [40, 48, 52, 68, 78, 90, 93, 103, 107, 110]

Living with PWS profoundly impacts the entire household, with caregivers (predominantly parents of those with PWS) as well as all family members facing a continuous, multifaceted burden that shapes daily life, relationships, and long-term wellbeing. Ten studies reported quotes from caregivers of PWS patients, highlighting the severe burden on families and caregivers, with themes encompassing family dynamics, lifestyle changes (including food security measures and strict routines), and social isolation (Fig. 4) [40, 48, 52, 68, 78, 90, 93, 103, 107, 110].

One study compared the burden between primary and secondary caregivers [60]. The primary caring parent had a significantly higher burden than other family members including the other parent, as measured by the Zarit burden interview (P = 0.0003). O’Neill et al. (2016) found that siblings of PWS patients had significantly higher anxiety than control siblings (P = 0.04) [88]. Additionally, Bos-Roubos et al. (2022) reported that 92.9% of PWS family members reported lifetime prevalence of trauma, compared to 80.7% in the Dutch general public, with 12% of these PWS family members reporting trauma severity indicative of post-traumatic stress disorder [36].

Caring for an individual with PWS is often detrimental to caregivers' careers. At a minimum, caregivers have reported the need to reduce their working hours by 8% to 16% across two studies, with 33% to 75% of caregivers required to abandon employment altogether [56, 93]. Kayadjanian et al. (2018) [66] found that 88% of working caregivers made changes to their work situation at least once a month due to PWS, and Ragusa et al. (2020) also reported that the impact on career prospects disproportionately affected female caregivers, with almost twice as many changing their jobs to accommodate caring responsibilities compared to their male counterparts (63% vs. 33%) [93].

A total of 17 studies were identified which reported on parent and caregiver burden in PWS as measured by QoL or burden scales [15, 16, 29, 31, 41, 42, 60, 66, 73, 74, 81, 82, 100, 107, 109, 116, 124]. Fifteen of these studies assessed either exclusively parents or reported that the majority of questioned caregivers were parents of the individual with PWS, of which six compared burden scores against healthy controls or other genetic disorders (Table 3). Quality of life was lower in PWS parents and caregivers than for those of matched normal populations [31, 124], and caregiver burden was higher in PWS caregivers than caregivers of patients with hemophilia, Duchenne muscular dystrophy, cystic fibrosis, and juvenile idiopathic arthritis [42].

**Table 3:** Studies of family and caregiver burden using quantitative scales

Study	Study design	Country, year	Study population	Scale	Mean (SD) PWS caregiver score	Mean (SD) Non-PWS caregiver score	*P*-value
Family and caregiver burden compared with healthy control cohorts							
Amaro et al. [31]	Observational, cross-sectional study of patients from Brazilian databases, medical centers and social networks	Brazil, NR	Parents of Brazilian PWS patients (N = 40) vs Brazilian general population (N = NR)	WHOQOL-BREF	Physical: 60.91 (10.64) Psychological: 60.33 (8.12) Social relation: 60.53 (11.56) Environment: 56.75 (9.64) Self-assessment: 58.8 (10.95) Total: 59.29 (8.18)	Brazilian General Population: Physical: 58.9 (10.5) Psychological: 65.9 (10.8)*** Social relation: 76.2 (18.8)*** Environment: 59.9 (14.9)* Self-assessment: NR Total: NR	*P < 0.05, ***P < 0.001 vs PWS
Mao et al. [124]	Observational, cross-sectional study of patients from hospital educational and support meetings	China, NR	Caregivers of PWS patients (N = 32) vs Chinese normal population (N = NR)	WHOQOL-BREF (Chinese version)	Physical: 12.8 (2.9) Psychological: 12.8 (2.9) Social: 12.4 (3.9) Environmental: 11.4 (3.6)	Physical: 15.1 (2.3)*** Psychological: 13.9 (1.9)* Social: 13.9 (2.1)* Environmental: 12.1 (2.1)	*P < 0.05, ***P < 0.001 vs PWS
Tvrdik et al. [109]	Observational, cross-sectional study of patients from a US PWS database	US, NR	Parents of PWS patients and US normal cohort	PSS-14	26.5	Value not reported, but lower than PWS cohort “by almost one standard deviation”	NR
Family and caregiver burden compared with other diseases							
Chiarotti et al. [42]	Observational, cross-sectional study performed by a rare disease center, rare disease federations and patient organizations	Italy, 2012	Family caregivers of patients with PWS Hem (N = 14), DMD (N = 50), Scl (N = 16), CF (N = 40), FXS (N = 12), His (N = 6), Muc (N = 16), JIA (N = 8) and EB (N = 14)	Zarit burden interview	36.7 (12.9)	Hem: 16.4 (5.7)* DMD: 25.4 (10.3)* Scl: 26.1 (10.8) CF: 24.6 (9.8)* FXS: 34.6 (12.7) His: 33.0 (20.9) Muc: 32.1 (12.3) JIA: 16.9 (7.0)* EB: 32.4 (14.7) Overall: 27.4 (12.5)	*Significantly lower burden than PWS (P-value not reported)
			Family caregivers of patients with PWS (N = 22), Hem (N = 14), DMD (N = 49), Scl (N = 14), CF (N = 39), FXS (N = 12), His (N = 6), Muc (N = 16), JIA (N = 8) and EB (N = 13)	EQ-5D-VAS	76.8 (16.0)	Hem 82.1 (15.3) MD: 79.4 (14.2) Scl: 71.4 (20.6) CF: 82.4 (12.1) FXS: 76.7 (11.3) His: 77.5 (17.3) Muc: 76.1 (19.5) JIA: 79.4 (18.0) EB: 78.1 (16.7) Overall: 78.7 (15.3)	NR
Thompson, et al. [107]	Observational, cross-sectional study of patients from Western Australia Disability and Genetic services databases	Australia, 2008	Parents of patients with PWS (N = 6) and AS (N = 13)	Family stress and coping interview	40.3 (NR)	32.6 (NR)	NR
Adams et al. [29]	Observational, cross-sectional study of patients from UK support groups	UK, NR	Parents of patients with PWS (N = 101), AS (N = 28), ASD (N = 66), CdLS (N = 44), DS (N = 29), FXS (N = 102), PMS (N = 31), RTT (N = 87), RTS (N = 47), SMS (N = 20) Soto (N = 38), TSC (N = 71), 1p36 (N = 26) and 8p23 (N = 22)	Positive gain score	21.06 (NR)	ASD: 21.66 (NR) AS: 21.55 (NR) CdLS: 21.66 (NR) DS: 22.42 (NR) FXS: 21.66 (NR) PMS: 22.98 (NR) RTT: 22.71 (NR) RTS: 22.70 (NR) SMS: 21.70 (NR) Soto: 21.78 (NR) TSC: 21.64 (NR) 1p36: 21.64 (NR) 8p23: 21.89 (NR)	NR
				Positive affect scale-5	15.65 (NR)	ASD: 15.25 (NR) AS: 16.60 (NR) CdLS: 15.51 (NR) DS: 15.93 (NR) FXS: 15.77 (NR) PMS: 16.03 (NR) RTT: 17.23 (NR) RTS: 15.07 (NR) SMS: 15.62 (NR) Soto: 15.53 (NR) TSC: 15.32 (NR) 1p36: 15.63 (NR) 8p23: 16.86 (NR)	NR
				Hospital anxiety and depression scale	6.12 (NR)	ASD: 7.12 (NR) AS: 6.03 (NR) CdLS: 6.92 (NR) DS: 4.79 (NR) FXS: 5.61 (NR) PMS: 6.13 (NR) RTT: 4.70 (NR) RTS: 5.36 (NR) SMS: 7.66 (NR) Soto: 5.42 (NR) TSC: 5.88 (NR) 1p36: 6.96 (NR) 8p23: 4.82 (NR)	NR
				QRSF-parent and family problems subscale	4 (NR)	ASD: 6 (NR) AS: 5 (NR) CdLS: 5 (NR) DS: 3 (NR) FXS: 4 (NR) PMS: 5 (NR) RTT: 4 (NR) RTS: 5 (NR) SMS: 6 (NR) Soto: 4 (NR) TSC: 5 (NR) 1p36: 5 (NR) 8p23: 4 (NR)	NR
Family and caregiver burden, no comparator							
Lopez-Bastida et al. [73]	Observational, cross-sectional study of outpatients in European countries	UK, Spain, France, Bulgaria, Germany, Sweden and Italy, 2011–2013 (Hungary also included but reported no caregiver values)	Caregivers from the UK (N = 17), Spain (N = 41), France (N = 21), Bulgaria (N = 4), Germany (N = 33), Sweden (N = 6) and Italy (N = 30)	EQ-5D utilities	UK: 0.728 (0.240) Spain: 0.742 (0.241) France: 0.740 (0.246) Bulgaria: 0.764 (0.317) Germany: 0.820 (0.195) Sweden: 0.773 (0.155) Italy: 0.824 (0.227)	NR	NR
				EQ-5D-VAS	UK: 70.3 (16.6) Spain: 72.5 (19.9) France: 71.3 (18.6) Bulgaria: 80.0 (8.2) Germany: 81.5 (11.9) Sweden: 81.0 (12.4) Italy: 77.8 (16.8)	NR	NR
				Zarit burden interview	UK: 44.23 (14.96) Spain: 44.13 (14.98) France: 41.74 (19.44) Bulgaria: NR Germany: 28.21 (10.15) Sweden: 27.00 (18.85) Italy: 36.96 (14.94)	NR	NR
Maccarone et al. [74]	Observational, cross-sectional study of patients from an Italian pediatric endocrinology outpatient clinic	Italy, 2020–2021	Parent caregivers of PWS patients (N = 12)	Zarit burden interview	41.81 (NR)	NR	NR
Rozensztrauch et al. [100]	Observational, cross-sectional study of PWS patients from a Polish rehabilitation and education clinic in	Poland, NR	Parents of PWS patients (N = 46)	PedsQL family impact module	Physical functioning: 27.7 (15.7) Emotional Functioning: 33.4 (15.7) Social functioning: 29.1 (15.5) Cognitive functioning: 28.9 (18.8) Communication: 29.9 (14.7) Worry: 24.2 (21.7) Daily activities: 27.6 (16.7) Family relations: 33.6 (18.2) Parent HRQoL: 29.4 (13.4) Family functioning: 28.9 (18.8) Total impact score: 29.6 (13.8)	NR	NR
Wong et al. [116]	PSI/SF no comparator		Parents of PWS patients (N = 67)	Parenting stress index/ short form	Raw total score: 99.2 (24.4)	NR	NR

*AS* Angelman syndrome, *ASD* autism spectrum disorder, *CdLS* Cornella de Lange syndrome, *CF* cystic fibrosis, *DMD* Duchenne muscular dystrophy, *DS* Down syndrome, *EB* epidermolysis bullosa, *EQ-5D-VAS* EQ-5D visual analogue scale, *FXS* fragile X syndrome, *Hem* hemophilia, *His* Histiocytosis, *JIA* juvenile idiopathic arthritis, *Muc* mucopolysaccharidosis, *NR* not reported, *PMS* Phelan McDermid syndrome, *PWS* Prader-Willi syndrome, *RTS* Rubenstein Taybi syndrome, *RTT* Rett syndrome, *Scl* scleroderma, *SD* standard deviation, *SMS* Smith-Magenis syndrome, *TSC* tuberous sclerosis complex, *WHOQOL-BREF* The World Health Organization quality of life: brief version, *1p36* 1p36 deletion syndrome, *8p23* 8p23 deletion syndrome

Statistical significance is denoted by: * P < 0.05, ** P < 0.01, *** P < 0.001

Seven studies reported on family and caregiver burden across different age groups, which demonstrated that the burden of looking after a child with PWS increased with age, peaking at adolescence and early adulthood (Table 4) [15, 16, 52, 65, 66]. This change in burden with increased age has been attributed to a shift from concerns over the time requirements for care and the financial impact, to managing the individual’s anxiety and challenging behaviors [15]. Additionally, an increase in caregiver burden was shown to positively correlate with hyperphagia, which worsens as infants progress to later stages of childhood through to adolescence and adulthood [3, 15]. It should be considered that a limited sample of adults with PWS reported a diminished sensation of insatiability in a prospective nutritional study [3, 125]. However, further research involving a larger cohort of adult PWS patients is necessary to accurately characterize hyperphagia within this sub-population [3, 125].

**Table 4:** Caregiver burden by age

Study	Study design	Country, year	Study population	Scale	Mean (SD) (unless otherwise stated) Results	*P*-value
Chevereul et al. [41]	Retrospective, cross-sectional study of individuals from the French PWS patient association	France, 2012–2013	Carers of adult (N = 26) and child (N = 12) PWS patients	Zarit burden interview	All: 41.7 (19.4) Children: 44.7 (16.7) Adults: 39.1 (22.2)	NR
				EQ-5D-5L utility	All: 0.74 (0.25) Children: 0.69 (0.27) Adults: 0.76 (0.25)	NR
				EQ-5D-5L-VAS	All: 71.3 (18.6) Children: 69.4 (22.1) Adults: 73.1 (15.6)	NR
Mackay et al. [75]	Observational, cross-sectional survey of Australian PWS registry patients	Australia, 2019–2020	Caregivers of PWS patients aged < 3 years (N = 6), 3–12 years (N = 25) and 13–25 years (N = 21)	SF-12v2	Median (IQR) Mental Component Summary: All: 42.0 (33.4–51.5) < 3 years: 41.0 (32.0–46.4) 3–12 years: 46.4 (35.3–52.6) 13–25 years: 40.0 (30.6–51.3) Median (IQR) Physical Component Summary: All: 52.5 (44.8–55.5) < 3 years: 56.5 (54.8–61.4) 3–12 years: 52.7 (47.3–54.3) 13–25 years: 50.5 (42.8–53.8) Median (IQR) Mental Raw Sum Score: All: 21.5 (17.5–25.5) < 3 years: 21.5 (18.0–23.0) 3–12 years: 23.0 (19.0–26.0) 13–25 years: 19.0 (16.0–24.0) Median (IQR) Physical Raw Sum Score: All: 21.0 (18.0–23.5) < 3 years: 22.0 (21.0–24.0) 3–12 years: 21.0 (19.0–23.0) 13–25 years: 20.0 (15.0–24.0)	NS
Ihara et al. [65]	Observational, cross-sectional study of patients from a single Japanese hospital	Japan, NR	Family caregivers of PWS children aged 6–12 years (N = 22) and adolescents aged 13–19 years (N = 23)	WHOQOL-BREF (Japanese version)	Total QoL 6–12 years: 3.50 (0.46) 13–19 years: 3.42 (0.43) Physical 6–12 years: 3.60 (0.63) 13–19 years: 3.57 (0.48) Psychological 6–12 years: 3.48 (0.52)* 13–19 years: 3.21 (0.53)* Social 6–12 years: 3.56 (0.53)* 13–19 years: 3.45 (0.66)* Environmental 6–12 years: 3.40 (0.45) 13–19 years: 3.45 (0.51) QoL Impression 6-12 years: 3.48 (0.68)* 13–19 years: 3.33 (0.73)*	*P < 0.05 between pairs, for mUPD genotype only
Kayadjanian, et al. [66]	Observational, cross-sectional study of patients recruited from two leading US PWS advocacy Facebook groups	US, 2016	Caregivers of PWS patients aged 0–4 years (N = 25), 5–11 years (N = 33), 12–18 years (N = 38), 19–30 years (N = 34) and 31 + years (N = 12)	Zarit burden interview	0–4 years: 34.8 (12.5) 5–11 years: 43.1 (17.5) 12–18 years: 49.2 (14.6)* 19–30 years: 49.2 (14.1)* 31 + years: 38.6 (10.5)	*P < 0.05 vs 0–4 year group
Kayadjanian, et al. [15]	Observational, cross-sectional study of patients recruited from two leading US PWS advocacy Facebook groups	US, NR	Caregivers of PWS patients aged 0–4 years (N = 53), 5–11 years (N = 70), 12–18 years (N = 44), 19–30 years (N = 33) and 31 + years (N = 4)	Zarit burden interview	0–4 years: 33.76 (12.67) 5–11 years: 45.07 (14.99)* 12–18 years: 48.96 (14.51)* 19–30 years: 51.15 (16.76)* 31 + years: 40.25 (16.46)	*P < 0.05 vs 0–4 year group
Meade et al. [16]	Observational, cross-sectional study of patients from an Irish tertiary referral center	Ireland, NR	Parents of PWS patients aged 0–5 years (N = 9), 5–12 years (N = 3) and 12 + years (N = 5)	PedsQL family impact module	Physical: All: 67.8 (18.85) 0–5 years: 74.53 (14.04)* 5–12 years: 80.55 (16.84)* 12 + years: 48.33 (13.69) Emotional: All: 60.29 (20.95) 0–5 years: 65.00 (24.23) 5–12 years: 65.00 (17.32) 12 + years: 49.00 (14.31) Social: All: 64.7 (23.27) 0–5 years: 79.16 (14.65)*** 5–12 years: 66.66 (25.25)* 12 + years: 37.5 (4.41) Cognitive: All: 67.64 (16.21) 0–5 years: 0.55 (15.29) 5–12 years: 71.66 (25.65) 12 + years: 60.00 (12.24) Communication: All: 54.89 (24.66) 0–5 years: 58.32 (18.63) 5–12 years: 80.55 (19.24)* 12 + years: 33.32 (21.24) Worry: All: 38.82 (15.66) 0–5 years: 37.77 (19.38) 5–12 years: 46.66 (2.88) 12 + years: 36.00 (12.94) Daily Activities: All: 54.4 (23.02) 0–5 years: 66.65 (15.59)** 5–12 years: 63.88 (9.62)* 12 + years: 26.66 (14.9) Family Relationships: All: 62.64 (23.45) 0–5 years: 76.11 (11.6)*** 5–12 years: 75.00 (15.0)*** 12 + years: 31.00 (6.51) Parent HRQoL: All: 65.29 (16.8) 0–5 years: 72.08 (13.5)* 5–12 years: 71.66 (20.2) 12 + years: 49.25 (10.4) Family Functioning: All: 58.09 (21.93) 0–5 years: 72.57 (8.52)*** 5–12 years: 63.54 (19.19)** 12 + years: 28.75 (5.13) Total Impact Score: All: 58.94 (16.05) 0–5 years: 66.27 (11.1)** 5–12 years: 65.97 (17.5)* 12 + years: 41.52 (9.4)	*P < 0.05, **P < 0.01, ***P < 0.001 vs 12 + year group
Dykens et al. [52]	Scale validation study conducted on PWS patients from the Global PWS Patient Registry	Global, NR	Parents of PWS patients aged 5–12 years (N = 268), 13–19 years (N = 247) and 20–59 years (N = 228)	Food safe zone	Alerting others, community supervision: 5–12 years: 15.12 (3.14)*** 13–19 years: 14.21 (3.27)*** 20–59 years: 13.95 (3.00)*** Lock, restrict food sources: 5–12 years: 11.62 (4.71)*** 13–19 years: 13.58 (5.43)*** 20–59 years: 16.22 (5.00)*** Check for food: 5–12 years: 4.42 (2.30)*** 13–19 years: 5.53 (2.74)*** 20–59 years: 5.91 (2.71)*** At home supervision, meals: 5–12 years: 12.67 (3.00) 13–19 years: 12.73 (3.05) 20–59 years: 12.80 (2.64) Avoid food settings: 5–12 years: 3.77 (1.76)*** 13–19 years: 4.37 (1.91)*** 20–59 years: 4.43(1.89)*** Total score: 5–12 years: 47.60 (10.40)*** 13–19 years: 50.42 (12.03)*** 20–59 years: 53.36 (10.94)***	***P < 0.001 between age groups

Statistical significance is denoted by: * P < 0.05, ** P < 0.01, *** P < 0.001

*EQ-5D-VAS* EuroQol-5D visual analogue scale, *IQR* interquartile range, *mUPD* maternal uniparental disomy, *NR* not reported, *NS* not significant, *PedsQL* Pediatric Quality of Life Inventory, *PWS* Prader-Willi syndrome, *QoL* quality of life, *SD* standard deviation, *SF-12v2* short form 12 item health survey version 2, *WHOQOL-BREF* The World Health Organization quality of life: brief version

### Economic burden of PWS

In this review, 33 publications reporting on 31 studies were identified that reported on cost and/or healthcare resource use (HCRU) in PWS [15, 18, 19, 38, 41, 56, 66, 71, 73, 92, 93, 126–147], including three economic evaluations [38, 71, 140], 4 publications with both cost and HCRU [18, 56, 66, 144], 10 publications with cost data only [19, 41, 73, 93, 126, 128, 135, 141, 142, 145], and 16 publications with HCRU only [15, 92, 127, 129–134, 136–139, 143, 146, 147].

#### Total, direct, and indirect costs of PWS

**Fig. 5:**
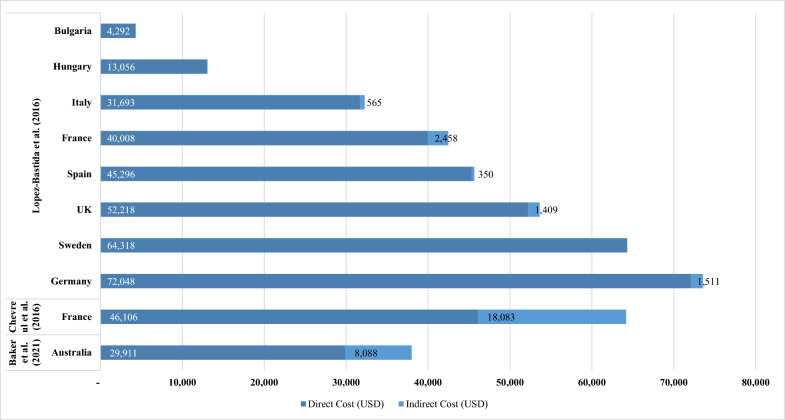
Direct and indirect annual costs associated with PWS by country and study. Note: All presented costs have been converted to USD using exchange rates as of 1 November 2024: 1 AUD = 0.66 USD, and 1 EUR = 1.09 USD. Abbreviations: AUD, Australian Dollar; EUR, Euro; PWS, Prader-Willi syndrome; USD, United States Dollar. Sources: Lopez-Bastida et al. [73]; Chevreul et al. [41]; Baker et al. [126]

Of the articles identified that reported on the cost of PWS, three studies provided a breakdown of total costs into direct (e.g. medication costs, inpatient, and outpatient care) and indirect (e.g. work productivity, specialist adaptations, and schooling) components [41, 73, 126]. Across the three studies, total costs per year ranged from 4,292 USD in a Hungarian PWS cohort to 73,559 USD in a German PWS cohort (Fig. 5).

Two large US claims database analyses demonstrated significantly higher direct medical costs in PWS patients compared to matched controls [128, 144]. Butler et al. (2020) analyzed costs for 1,621 PWS patients and 8,105 non-PWS matched controls, finding that mean direct medical costs for PWS patients were approximately ten times higher (35,197 USD vs 3,529 USD per patient annually). These differences were most pronounced in patients under 18 years, where mean costs were 12.1–14.7 times higher in PWS patients compared to controls [128]. Similarly, Shoffstall et al. (2016) found that commercially insured PWS patients had an 8.8-fold higher total annual direct medical costs compared to matched controls (28,712 USD vs 3,246 USD), with significant differences in outpatient care (11,032 USD vs 1,804 USD), inpatient costs (10,879 USD vs 1,015 USD), and medication costs (6,801 USD vs 428 USD) [144]. For Medicaid-insured patients, the disparity was also substantial, with PWS patients incurring 7.7-fold higher total annual costs compared to matched controls (40,868 USD vs 5,306 USD) [144]. In the BUROQoL European study conducted across eight countries, cost components in 2012 were reported as direct non-healthcare costs (ranging from 311 EUR [Bulgaria] to 18,760 EUR [France]) and healthcare costs (ranging from 1,387 EUR [Bulgaria] to 49,610 EUR [Germany]) [41, 73]. With this study specifying informal care (by caregivers) as a direct non-healthcare cost, the data highlights the major cost burden faced by caregivers in looking after individuals with PWS [41, 73]. Additionally, caregivers were more likely to take sick leave (on account of anxiety/stress), reduce working hours or give up work entirely (early retirement) to facilitate supervision and care of family members with PWS, with associated costs ranging from 321 EUR (Spain) to 2,205 EUR (France) [73]. A similar trend was reported in Baker et al. (2021), an Australian retrospective cohort study which reported 12,256 AUD lost income per PWS individual per year indicating the detrimental impact on the careers of caregivers by the need for full-time care and supervision [126].

#### Resource use

A total of 20 publications, reporting on 19 distinct studies, were identified that reported on patient resource use including hospitalization rate, outpatient service use, length of hospital stays, residential status, and, drug/procedure use [15, 18, 56, 66, 92, 127, 129–134, 136–139, 143, 144, 146, 147]. Across two US studies reporting on the residential status of individuals with PWS, 88–96% of people with PWS lived in their family home, with > 68% of individuals being children or adolescents [15, 66]. In adults with PWS, living in the family home was less common (25% to 50%), with the remaining individuals predominantly residing in institutional residential facilities, either specialized PWS group homes or if unavailable, in adapted non-specialized facilities, as reported in two studies in France and the Netherlands [134, 138].

#### Hospitalization rates and drivers of admission

Hospitalization rates and drivers of admission in PWS patients were reported in eight studies [18, 92, 127, 130, 135, 139, 144, 147] (Table 5). Data from an identified retrospective study of hospitalized PWS patients in the US reported a mean of 1.46 admissions per patient over the one-year study period [total 703 in 480 patients] across a variety of complications including respiratory complications (22%), infections (15%), neuropsychiatric diagnoses (13%), and gastrointestinal conditions (12%) [139]. Two studies of PWS neonates in France and China reported hospitalization figures of 93% and 95% (respectively) shortly following birth due to feeding problems, hypotonia, and respiratory problems indicating the potential severity of PWS in early life [127, 147]. For psychiatric-related hospitalizations, two studies of PWS cohorts, one in France, the other global, showed frequent admissions of adults and children ranging from 44.7% to 48.3% [92, 130]. Finally, two US retrospective claims database studies compared PWS patients with matched non-PWS controls revealing a trend of elevated hospitalization rates with PWS from a payer perspective [18, 144].

**Table 5:** Hospitalization rates and drivers in PWS patients

Study references	Study design	PWS study population (n)	Hospitalization rate	Hospital admission driver
Luccarelli et al. [18]	Cross-sectional analysis using National Inpatient Sample (NIS) claims database	540 (hospitalized adults and children)	Elective: 10.2% Non-elective: 89.8%	Various secondary diagnoses in hospitalized PWS patients
Yang et al. [147]	Patient registry analysis	134 (newborns and children)	94.8%*	Feeding problems, hypotonia and respiratory problems
Peleggi et al. [92]	Patient registry analysis	750 (adults and children)	Total hospitalizations: 13.5% (101) SI group: 44.7% (42) No SI group: 9% (9)	Psychiatric problems
Pemmasani et al. [139]	Retrospective analysis using the Healthcare Cost and Utilization Project (HCUP) Nationwide Readmissions Database (NRD) 2014	480 (hospitalized adults and children)	Total hospitalizations: 703 Mean number per patient: 1.46	Respiratory complications (including pneumonia): 22% Infections (including sepsis): 15% Neuropsychiatric diagnoses: 13% GI conditions: 12%
McQuivey et al. [136]	Retrospective cohort study	3684 (hospitalized children)	100% (9.1% for orthopedic surgery)	Orthopedic surgery (scoliosis-related)
Clerc et al. [130]	Multicenter retrospective descriptive study	39 (adults and children)	48.3%	Psychiatric problems
Bar et al. [127]	Retrospective cohort study	61 (newborns)	Neonates hospitalized: 93% (57/61)	Feeding problems, hypotonia and respiratory problems
Shoffstall et al. [144]	Retrospective case-matched control design using longitudinal US administrative claims data (MarketScan) during a 5-year enrollment period (2009–2014)	N = 2030 (adults and children) Commercially insured: 1161/2030 (57.2%) Medicare supplemental: 38/2030 (1.9%) Medicaid: 831/2030 (40.9%)	Mean PPPY (SD) Inpatient Admissions: Commercially & Medicare PWS: 0.28 (NR) Non-PWS: 0.05 (NR) Medicaid-insured patients PWS: 0.33 (NR) Non-PWS: 0.16 (NR)	NR

^*^Patients requiring hospital treatment in the neonatal period

*GI* gastrointestinal, *NR* not reported, *PPPY* per patient per year, *PWS* Prader-Willi syndrome, *SD* standard deviation, *SI* suicidal ideation

#### Hospital length of stay

With regards to hospital length of stay, nine studies were identified with mean durations ranging from 2.73 days to 75.9 days [18, 127, 129, 131, 135–137, 144, 145]. Most studies (n = 7) fell in the range of 2.73 to 7.74 days (mean) with longer stays reported for newborn hospitalizations. The longest hospital stays were reported by an inpatient treatment program for pediatric and adult PWS patients [131]. The greater length of stay in this study can be explained by the fact patients were admitted in medical or behavioral crisis, having likely failed outpatient and conventional treatment approaches, and therefore required longer lengths of stay to achieve treatment responses [131]. 

#### Medication use

From 13 publications encompassing 12 studies identified in this review, the complexity of PWS was reflected by the polypharmacy reported [18, 92, 127, 130–132, 136–138, 143, 144, 146, 147]. Typical medications prescribed included anti-diabetics, hormones (such growth hormone and sex hormones) and psychotropics, with a median of 6 (IQR 4.5–7) medications per patient reported by a French retrospective cohort study [130]. In a US claims analysis comparison with non-PWS matched controls, PWS patients reported higher usage of cardiovascular, central nervous system, and hormone replacement medications according to the difference in all-age (0 to 65 years) drug exposure frequency (P < 0.0001) [144]. Two studies in Ireland and Netherlands reported that 35% to 74% of children with PWS were receiving growth hormone at the time of analysis (2015 to 2020 and 2021, respectively) [137, 138]. Additionally, antipsychotic medications were reported in three studies which may be due to the inherent behavioral and psychological issues associated with PWS [92, 130, 131].

## Discussion

We conducted an SLR to provide a descriptive summary of the burden of PWS on patients, caregivers, and healthcare systems. PWS was reported to reduce life expectancy, severely impact the quality of life of patients and their caregivers, and pose a substantial economic burden for healthcare systems and affected families. Our findings demonstrate the clear unmet need patients and caregivers experience for comprehensive support and innovative treatment options that directly manage the symptoms of PWS, particularly hyperphagia.

PWS patients face a significantly elevated mortality risk, with rates up to 27 times higher than the general population in some age groups [9]. Respiratory failure was identified as the primary cause of death, with hyperphagia-related causes second, followed by cardiovascular complications [22, 23, 25, 27]. Uncontrolled hyperphagia in PWS is associated with high rates of choking, accidents, and GI-perforation which increases mortality risk markedly in childhood [22]. Life expectancy was notably reduced, averaging 23–32 years across the available literature [22, 24, 25, 27]. However, it should be noted that in the literature captured by this search procedure, data on the age at death is only available from cohorts monitored up to as recently as 2015.

Hyperphagia is a characteristic feature of PWS, which is accompanied with relentless hunger, food-seeking behaviors, and emotional distress [8, 148]. Additionally, behavioral and emotional issues like anxiety, compulsivity, and irritability frequently surpass clinically meaningful thresholds, impacting patients’ daily and social functioning [44, 50, 53, 72, 75, 85, 99, 106, 108, 119].

Caregivers are often the parents of those with PWS and report significant emotional strain, career disruptions, and social isolation as they manage the demands of PWS, often at the cost of their own wellbeing [40, 48, 52, 56, 66, 68, 78, 90, 93, 103, 107, 110]. Food control measures (which can include locking mechanisms for the fridge, home alarms, and strict routines with limited social events) were highlighted as essential to manage hyperphagia [52], yet these measures result in extreme lifestyle changes, leading to social isolation [48] and reduced work hours [56, 93]. Social misunderstandings further compound their struggles, with caregivers often feeling misunderstood by others, including extended family and educational or medical professionals [40, 103, 110]. As such, caregiver quality of life was lower in PWS parents and caregivers than for those of matched normal populations [31, 124] or caregivers of patients with other chronic conditions [42].

The burden of PWS and associated food control measures also affects the wider family dynamic, with higher rates of anxiety and trauma observed in siblings of those with PWS compared with controls or the general population [36, 88].

PWS incurs high economic costs, driven by healthcare provision and informal caregiving [41, 73, 126]. US data indicated that PWS-related direct medical costs can be 9–10 times higher than for non-PWS individuals, with even greater disparity seen in younger patients [128, 144].

### Strengths and limitations

To our knowledge this is the first SLR on the burden of disease in PWS. The strengths of this SLR lie in its comprehensive nature, utilizing MEDLINE, Embase, The Cochrane Library, DARE, NHS EED, HTA database and EconLIT. Additionally, SLR references were searched and grey literature from nine key conferences was assessed. This was combined with a well-designed search strategy developed by a team of experienced systematic reviewers.

The comprehensiveness of this SLR, along with the volume of available literature, meant that searches had to be limited to English language papers the last ten years (2014–2024). Thus, some information pertaining to the burden of disease in PWS may have been missed. However, a focus on more recent data may yield results that are potentially more reflective of the burden of PWS in light of improvements in disease understanding and management over the last decade.

Despite numerous studies focusing on multiple age groups, many, particularly those assessing humanistic burden, were cross-sectional, potentially failing to capture the longitudinal impact of PWS throughout a patient’s lifetime. Additionally, sample sizes were variable and drawing conclusions from some of the smaller studies may be difficult. When assessing QoL or clinical grading scores, most studies did not include suitable non-PWS controls. As a result, the values in these studies are hard to interpret. Furthermore, the vast use of numerous different QoL tools made comparisons between study cohorts difficult to standardize.

Cost and resource use data studies were primarily from developed countries, mostly US and Europe. There may therefore be an associated bias towards the healthcare preferences in these more developed countries. This may have been influenced by the English-language-only search criteria used in this review.

Additionally, it is worth considering the reliance on ICD coding to identify patients with PWS which may lead to underrepresentation in retrospective cohort studies. Specifically, ICD-10 coding does not necessarily capture the hyperphagia-related direct and indirect burden of PWS. Despite being widely accepted as the hallmark feature of PWS, there is no specific ICD code for hyperphagia which makes contribution towards mortality and economic burden difficult to capture in database analyses. Furthermore, while it is accepted that hyperphagia is a hallmark of PWS in most individuals, the heterogenous nature of the disease is reflected by a small minority of cases not exhibiting typical hyperphagia food-seeking behaviors (independent of environmental restrictions and food control measures) [3, 149].

### Future research directions

To fully understand the multifaceted impact of PWS on patients, their families, and the healthcare system, it is crucial to prioritize research that explores its economic, clinical, and social dimensions, as well as more recent estimates of mortality. A further focus on the economic impact of PWS in less-developed countries would help build a more global picture of the burden of disease. Larger cohort studies, comparing QoL and clinical measures in PWS and healthy patients or those with similar diseases, especially with a longitudinal design, would drastically improve the quality of future conclusions on the burden of PWS for patients. In addition, a consensus among PWS researchers on using a more focused number of QoL tools would allow for enhanced future comparisons of study cohorts. Finally, additional work linking the symptoms of PWS, particularly hyperphagia, to patient QoL, mortality and family/caregiver burden may shine further light on the unmet treatment needs of these patients.

## Conclusions

PWS is associated with reduced life expectancy, severe quality of life impairment, and high economic costs, imposing a significant burden on patients, caregivers, and healthcare systems. Hyperphagia, a hallmark of the syndrome, is a leading cause of mortality and is associated with increased caregiver burden. These findings underscore the need for comprehensive resources to support both the psychosocial and practical aspects of PWS care. Further research is essential to explore the long-term impacts of PWS and the associated caregiver burden, particularly in underrepresented populations and regions, to inform future interventions and support mechanisms.

## Supplementary Information

Additional file 1: Search strategy.

Additional file 2: Table of included studies, table of QoL scales.

## Data Availability

The datasets used and/or analyzed during the current study are available from the corresponding author on reasonable request.

## Abbreviations

1p36: 1P36 Deletion Syndrome
8p23: 8P23 Deletion Syndrome
AS: Angelman syndrome
ASD: Autism spectrum disorder
AUD: Australian dollar
CdLS: Cornelia de Lange syndrome
CF: Cystic fibrosis
CI: Confidence interval
CVD: Cardiovascular disease
DARE: Database of Abstracts and Reviews of Effects
DBC: Developmental Behavior Checklist
DMD: Duchenne muscular dystrophy
DS: Down syndrome
EB: Epidermolysis bullosa
EQ-5D: EuroQol 5 Dimension
EQ-5D-VAS: EuroQol-5 Dimension visual analogue scale
FXS: Fragile X syndrome
HCRU: Health care resource use
Hem: Hemophilia
His: Histiocytosis
HTA: Health technology assessment
IQR: Interquartile range
JIA: Juvenile idiopathic arthritis
Muc: Mucopolysaccharidosis
mUPD: Maternal uniparental disomy
NHS EED: NHS Economic Evaluation Database
NR: Not reported
NS: Not significant
PedsQL: Pediatric Quality of Life Inventory
PICOS: Population, intervention, comparator, outcomes and study design
PMS: Phelan McDermid syndrome
PWS: Prader-Willi syndrome
PWSP: Prader-Willi Syndrome Profile
QALY: Quality-adjusted life year
QoL: Quality of life
RR: Relative risk
RTS: Rubinstein-Taybi syndrome
RTT: Rett Syndrome
Scl: Scleroderma
SD: Standard deviation
SF-12v2: Short form 12 item health survey version 2
SLR: Systematic literature review
SMS: Smith-Magenis Syndrome
T2DM: Type 2 diabetes mellitus
TSC: Tuberous sclerosis complex
TTO: Time-trade off
US: United States
USD: United States dollar
WHOQOL-BREF: The World Health Organization quality of life: brief version

## References

[1] Angulo MA, Butler MG, Cataletto ME. Prader-Willi syndrome: a review of clinical, genetic, and endocrine findings. J Endocrinol Invest. 2015;38(12):1249–63. 26062517 10.1007/s40618-015-0312-9 PMC4630255

[2] Cassidy SB, Schwartz S, Miller JL, Driscoll DJ. Prader-Willi syndrome. Genet Med. 2012;14(1):10–26. 22237428 10.1038/gim.0b013e31822bead0

[3] Driscoll DJ, Miller J. L., Cassidy S. B. Prader-Willi Syndrome. Adam M. P. FJ, Mirzaa G. M., Pagon R. A., Wallace S. E., Bean L. J. H., et al, editor. GeneReviews2023.

[4] Manzardo AM, Weisensel N, Ayala S, Hossain W, Butler MG. Prader-Willi syndrome genetic subtypes and clinical neuropsychiatric diagnoses in residential care adults. Clin Genet. 2018;93(3):622–31. 28984907 10.1111/cge.13142 PMC5828945

[5] Bohonowych J, Miller J, McCandless SE, Strong TV. The global Prader-Willi syndrome registry: development, launch, and early demographics. Genes (Basel). 2019;10(9):14. 10.3390/genes10090713 PMC6770999 31540108

[6] Disorders NOfR. Prader-Willi Syndrome. 2023.

[7] Miller JL, Tan M. Dietary Management for Adolescents with Prader-Willi Syndrome. Adolesc Health Med Ther. 2020;11:113–8. 32922110 10.2147/AHMT.S214893 PMC7457755

[8] Schwartz L, Caixàs A, Dimitropoulos A, Dykens E, Duis J, Einfeld S, Gallagher L, Holland A, Rice L, Roof E, Salehi P, Strong T, Taylor B, Woodcock K. Behavioral features in Prader-Willi syndrome (PWS): consensus paper from the International PWS Clinical Trial Consortium. J Neurodev Disord. 2021;13(1):25. 34148559 10.1186/s11689-021-09373-2 PMC8215770

[9] Hedgeman E, Ulrichsen SP, Carter S, Kreher NC, Malobisky KP, Braun MM, et al. Long-term health outcomes in patients with Prader-Willi Syndrome: a nationwide cohort study in Denmark. Int J Obes (Lond). 2017;41(10):1531–8. 28634363 10.1038/ijo.2017.139

[10] van Abswoude DH, Pellikaan K, Rosenberg AGW, Davidse K, Coupaye M, Hoybye C, et al. Bone health in adults with Prader-Willi syndrome: clinical recommendations based on a multicenter cohort study. J Clin Endocrinol Metab. 2022;108(1):59–84. 36149817 10.1210/clinem/dgac556 PMC9759176

[11] Muscogiuri G, Barrea L, Faggiano F, Maiorino MI, Parrillo M, Pugliese G, et al. Obesity in Prader-Willi syndrome: physiopathological mechanisms, nutritional and pharmacological approaches. J Endocrinol Invest. 2021;44(10):2057–70. 33891302 10.1007/s40618-021-01574-9 PMC8421305

[12] Bellicha A, Coupaye M, Hocquaux L, Speter F, Oppert J, Poitou CM. Increasing physical activity in adult women with Prader-Willi syndrome: a transferability study. J Appl Res Intellect Disabil. 2020;33(2):258–67. 31578803 10.1111/jar.12669

[13] Reus L, Zwarts M, van Vlimmeren LA, Willemsen MA, Otten BJ, Sanden MW. Motor problems in Prader-Willi syndrome: a systematic review on body composition and neuromuscular functioning. Neurosci Biobehav Rev. 2011;35(3):956–69. 21056055 10.1016/j.neubiorev.2010.10.015

[14] Morales JS, Valenzuela PL, Pareja-Galeano H, Rincon-Castanedo C, Rubin DA, Lucia A. Physical exercise and Prader-Willi syndrome: a systematic review. Clin Endocrinol (Oxf). 2019;90(5):649–61. 30788853 10.1111/cen.13953

[15] Kayadjanian N, Vrana-Diaz C, Bohonowych J, Strong TV, Morin J, Potvin D, Schwartz L. Characteristics and relationship between hyperphagia, anxiety, behavioral challenges and caregiver burden in Prader-Willi syndrome. PLoS ONE. 2021;16(3):e0248739. 33765021 10.1371/journal.pone.0248739 PMC7993772

[16] Meade C, Martin R, McCrann A, Lyons J, Meehan J, Hoey H, Roche E. Prader-Willi syndrome in children: quality of life and caregiver burden. Acta Paediatr. 2021;110(5):1665–70. 33378107 10.1111/apa.15738

[17] Paepegaey AC, Coupaye M, Jaziri A, Menesguen F, Dubern B, Polak M, Oppert JM, Tauber M, Pinto G, Poitou C. Impact of transitional care on endocrine and anthropometric parameters in Prader-Willi syndrome. Endocr Connect. 2018;7(5):663–72. 29666169 10.1530/EC-18-0089 PMC5952243

[18] Luccarelli J. Demographics and medical comorbidities among hospitalized patients with Prader-Willi Syndrome: a national inpatient sample analysis. Am J Med Genet A. 2022;188(10):2899–907. 35838073 10.1002/ajmg.a.62901 PMC9474715

[19] Avram CM, Allen AJ, Shaffer BL, Caughey AB. The impact of Prader Willi Syndrome on perinatal outcomes. Am J Obst Gynecol. 2018;218(1):S288–9.

[20] Bourke J, Wong K, Leonard H. Validation of intellectual disability coding through hospital morbidity records using an intellectual disability population-based database in Western Australia. BMJ Open. 2018;8(1):963. 10.1136/bmjopen-2017-019113 PMC5786126 29362262

[21] Butler MG, Kimonis V, Dykens E, Gold JA, Miller J, Tamura R, et al. Prader-Willi syndrome and early-onset morbid obesity NIH rare disease consortium: a review of natural history study. Am J Med Genet A. 2018;176(2):368–75. 29271568 10.1002/ajmg.a.38582 PMC6065257

[22] Butler MG, Manzardo AM, Heinemann J, Loker C, Loker J. Causes of death in Prader-Willi syndrome: Prader-Willi syndrome association (USA) 40-year mortality survey. Genet Med. 2017;19(6):635–42. 27854358 10.1038/gim.2016.178 PMC5435554

[23] Manzardo AM, Loker J, Heinemann J, Loker C, Butler MG. Survival trends from the Prader-Willi Syndrome Association (USA) 40-year mortality survey. Genet Med. 2018;20(1):24–30. 28682308 10.1038/gim.2017.92 PMC5756527

[24] McCandless SE, Suh M, Yin D, Yeh M, Czado S, Aghsaei S, et al. U.S. prevalence & mortality of Prader-Willi syndrome: a population-based study of medical claims. J Endocr Soc. 2020;4:A504–5.

[25] Pacoricona Alfaro DL, Lemoine P, Ehlinger V, Molinas C, Diene G, Valette M, et al. Causes of death in Prader-Willi syndrome: lessons from 11 years’ experience of a national reference center. Orphanet J Rare Dis. 2019;14(1):238. 31684997 10.1186/s13023-019-1214-2 PMC6829836

[26] Proffit JN, Osann K, MacManus B, Butler MG, Kimonis VE, Heinemann J, Stevenson D, Gold JA. A lower BMI and growth hormone use results in decreased mortality in Prader-Willi syndrome. Eur J Hum Genet. 2019;27(Supplement 2):1520–1. 10.1002/ajmg.a.60688 PMC6349475 30569567

[27] Proffitt J, Osann K, McManus B, Kimonis VE, Heinemann J, Butler MG, et al. Contributing factors of mortality in Prader-Willi syndrome. Am J Med Genet A. 2019;179(2):196–205. 30569567 10.1002/ajmg.a.60688 PMC6349475

[28] Whittington JE, Holland AJ, Webb T. Ageing in people with Prader-Willi syndrome: mortality in the UK population cohort and morbidity in an older sample of adults. Psychol Med. 2015;45(3):615–21. 25088280 10.1017/S0033291714001755

[29] Adams D, Hastings RP, Alston-Knox C, Cianfaglione R, Eden K, Felce D, Griffith G, Moss J, Stinton C, Oliver C. Using Bayesian methodology to explore the profile of mental health and well-being in 646 mothers of children with 13 rare genetic syndromes in relation to mothers of children with autism. Orphanet J Rare Dis. 2018;13(1):185. 30359268 10.1186/s13023-018-0924-1 PMC6203267

[30] Allas S, Caixàs A, Poitou C, Coupaye M, Thuilleaux D, Lorenzini F, Diene G, Crinò A, Illouz F, Grugni G, et al. AZP-531, an unacylated ghrelin analog, improves food-related behavior in patients with Prader-Willi syndrome: a randomized placebo-controlled trial. PLoS ONE. 2018;13(1):e0190849. 29320575 10.1371/journal.pone.0190849 PMC5761957

[31] Amaro AS, Rubin DA, Teixeira MFAJ, Rodrigues GM, Carreiro LRR. Health problems in individuals with PWS are associated with lower quality of life for their parents: a snapshot in the brazilian population. Front. 2022;10:746311. 10.3389/fped.2022.746311 PMC8885721 35242723

[32] Andrews SM, Panjwani AA, Potter SN, Hamrick LR, Wheeler AC, Kelleher BL. Specificity of early childhood hyperphagia profiles in neurogenetic conditions. Am J Intellect Dev Disabil. 2024;129(3):175–90. 38657964 10.1352/1944-7558-129.3.175

[33] Avrahamy H, Pollak Y, Shriki-Tal L, Genstil L, Hirsch HJ, Gross-Tsur V, Benarroch F. A disease specific questionnaire for assessing behavior in individuals with Prader-Willi syndrome. Compr Psychiatry. 2015;58:189–97. 25677112 10.1016/j.comppsych.2014.12.005

[34] Baietto C, Bechis D, Caldarera AM, Marcotulli D, Natali SM. Children with Prader-Willi Syndrome and COVID-19: a longitudinal study of the effect of social re-opening after the lockdown. Minerva Pediatr (Torino). 2023;26:26. 10.23736/S2724-5276.22.07036-7 36700944

[35] Bakker NE, Siemensma EP, van Rijn M, Festen DA, Hokken-Koelega AC. Beneficial effect of growth hormone treatment on health-related quality of life in children with Prader-Willi syndrome: a randomized controlled trial and longitudinal study. Hormone Res Paediat. 2015;84(4):231–9. 10.1159/000437141 26279206

[36] Bos-Roubos A, Wingbermuhle E, Biert A, de Graaff L, Egger J. Family matters: trauma and quality of life in family members of individuals with Prader-Willi syndrome. Front Psychiatr. 2022;13:897138. 10.3389/fpsyt.2022.897138 PMC9273751 35836666

[37] Bravo GL, Poje AB, Perissinotti I, Marcondes BF, Villamar MF, Manzardo AM, Luque L, LePage JF, Stafford D, Fregni F, Butler MG. Transcranial direct current stimulation reduces food-craving and measures of hyperphagia behavior in participants with Prader-Willi syndrome. Am J Med Genet B Neuropsychiatr Genet. 2016;171B(2):266–75. 26590516 10.1002/ajmg.b.32401 PMC6668339

[38] Bridges JF, Lavelle T, Tsai J, Kayadjanian N, Strong T. Assessing the potential impact of treating hyperphagia among people with prader-willi syndrome using disease-specific qalys. Value in Health. 2018;21(Supplement 1):S256.

[39] Butler MG, McCandless S, Roof E, Dykens EM, Fu C, Stafford DEJ, et al. Weight loss and improvement in hyperphagia-related behavior: results from bestpws, a phase 3, randomized, placebo-controlled, clinical trial of beloranib, a methionine aminopeptidase 2 (MetAP2) inhibitor, in patients with prader-willi syndrome. Endocrine Reviews Conference: 98th Annual Meeting and Expo of the Endocrine Society, ENDO. 2016;37(2 Supplement 1).

[40] Cagalj D, Buljevac M, Leutar Z. Being a mother of a child with Prader-Willi syndrome: experiences of accessing and using formal support in Croatia. Scand J Disabil Res. 2018;20(1):228–37.

[41] Chevreul K, Berg BK, Clement MC, Poitou C, Tauber M. Economic burden and health-related quality of life associated with Prader-Willi syndrome in France. J Intellect Disabil Res. 2016;60(9):879–90. 27174598 10.1111/jir.12288

[42] Chiarotti F, Kodra Y, De Santis M, Bellenghi M, Taruscio D, Care A, Petrini M. Gender and burden differences in family caregivers of patients affected by ten rare diseases. Ann Ist Super Sanita. 2023;59(2):122–31. 37337987 10.4415/ANN_23_02_05

[43] Chiu VJ, Tsai LP, Wei JT, Tzeng IS, Wu HC. Motor performance in Prader-Willi syndrome patients and its potential influence on caregiver’s quality of life. PeerJ. 2017;5:e4097. 29255649 10.7717/peerj.4097 PMC5732539

[44] Choong CS, Nixon GM, Blackmore AM, Chen W, Jacoby P, Leonard H, Lafferty AR, Ambler G, Kapur N, Bergman PB, Schofield C, Seton C, Tai A, Tham E, Vora K, Crock P, Verge C, Musthaffa Y, Blecher G, Wilson A, Downs J. Daytime sleepiness and emotional and behavioral disturbances in Prader-Willi syndrome. Eur J Pediatr. 2022;181(6):2491–500. 35316366 10.1007/s00431-022-04439-2 PMC9110445

[45] Cotter SP, Schwartz L, Strong TV, Bender RH, Fehnel SE. The Prader-Willi syndrome anxiousness and distress behaviors questionnaire: development and psychometric validation. Value in Health. 2023;26(2):243–50. 36202701 10.1016/j.jval.2022.08.004

[46] Coupaye M, Tauber M, Cuisset L, Laurier V, Bieth E, Lacorte JM, Oppert JM, Clement K, Poitou C. Effect of genotype and previous GH treatment on adiposity in adults with Prader-Willi syndrome. J Clin Endocrinol Metab. 2016;101(12):4895–903. 27662437 10.1210/jc.2016-2163

[47] Coutant R, Tauber M, Demaret B, Henocque R, Brault Y, Montestruc F, Chassany O, Polak M. Treatment burden, adherence, and quality of life in children with daily GH treatment in France. End Connect. 2023;12(4):52. 10.1530/EC-22-0464 PMC10083659 36866786

[48] Currie G, Estefan A, Caine V. Mothering a child with complexity and rarity: a narrative inquiry exploring Prader-Willi syndrome. Qual Health Res. 2024;52:5104. 10.1177/10497323231225412 PMC11323427 38282344

[49] Dobrescu A, Chirita-Emandi A, Andreescu N, Farcas S, Puiu M. Hyperphagia questionnaire to evaluate the Prader Willi patients behavior related to food. Eur J Hum Genet. 2019;26(Supplement 1):865–6.

[50] Downs J, Blackmore AM, Chen W, Nixon GM, Choong CS. Strengths and challenging behaviors in children and adolescents with Prader-Willi syndrome: two sides to the coin. Am J Med Genet A. 2022;188(5):1488–96. 35092339 10.1002/ajmg.a.62671

[51] Dykens EM, Roof E, Hunt-Hawkins H. The Prader-Willi syndrome Profile: validation of a new measure of behavioral and emotional problems in Prader-Willi syndrome. Orphanet J Rare Dis. 2024;19(1):83. 38395848 10.1186/s13023-024-03045-9 PMC10885615

[52] Dykens EM, Roof E, Hunt-Hawkins H. Validation of the food safe zone questionnaire for families of individuals with Prader-Willi syndrome. medRxiv. 2024;28:56. 10.1186/s11689-024-09589-y PMC11806870 39923017

[53] Einfeld SL, Smith E, McGregor IS, Steinbeck K, Taffe J, Rice LJ, Horstead SK, Rogers N, Hodge MA, Guastella AJ. A double-blind randomized controlled trial of oxytocin nasal spray in Prader Willi syndrome. Am J Med Genet A. 2014;164(9):2232–9. 10.1002/ajmg.a.36653 24980612

[54] EUCTR. A Clinical Study in patients with Prader-Willi-Syndrome (PWS) to test if a study drug named livoletide can reduce food related behaviour and be safe and well tolerated [Trial registry record]. 2019 [Available from: https://trialsearch.who.int/Trial2.aspx?TrialID=EUCTR2018-003062-13-ES]. Last accessed Last accessed: 31 January 2025.

[55] Faye S, Molinas, C., Brochado, C., Valette, M., Desprez, C., Diene, G., Arnaud, C., Tauber, M. The evolution of diagnosis and care over time in children with Prader-Willi syndrome, born between 2005 and 2021, included in the French database. European Societ for Paediatric Endocrinology meeting 2023. 2023.

[56] Feighan SM, Hughes M, Maunder K, Roche E, Gallagher L. A profile of mental health and behaviour in Prader-Willi syndrome. J Intellect Disabil Res. 2020;64(2):158–69. 31849130 10.1111/jir.12707

[57] Foerste T, Sabin M, Reid S, Reddihough D. Understanding the causes of obesity in children with trisomy 21: hyperphagia vs physical inactivity. J Intellect Disabil Res. 2016;60(9):856–64. 26936540 10.1111/jir.12259

[58] Friedman C. The personal outcome measures. Disabil Health J. 2018;11(3):351–8. 29274792 10.1016/j.dhjo.2017.12.003

[59] Giordano L, Toma S, Palonta F, Teggi R, Zucconi M, Di Candia S, Bussi M. Obstructive sleep apnea in Prader-Willi syndrome: risks and advantages of adenotonsillectomy. Pediatr Med Chir. 2015;37(2):8–11. 10.4081/pmc.2015.107 26429118

[60] Gonzalez Ruiz Y, Gerk A, Stegmann J. Mental health impact on primary and secondary Prader-Willi syndrome caregivers. Child Care Health Dev. 2024;50(1):e13162. 37614065 10.1111/cch.13162

[61] Grolleau S, Delagrange M, Souquiere M, Molinas C, Diene G, Valette M, Tauber M. Impact of deprivation on obesity in children with PWS. Journal. 2022;11(8):635. 10.3390/jcm11082255 PMC9031951 35456348

[62] Harisseh R, Delale T, Yeh M, Allas S. Livoletide (AZP-531), an unacylated ghrelin analogue, improves hyperphagia and food-related behaviors both in obese and non-obese people with Prader-Willi syndrome. J Endocrine Soc. 2020;4(Supplement 1):A510–1.

[63] Hollander E, Levine KG, Ferretti CJ, Freeman K, Doernberg E, Desilva N, Taylor BP. Intranasal oxytocin versus placebo for hyperphagia and repetitive behaviors in children with Prader-Willi syndrome: a randomized controlled pilot trial. J Psychiatr Res. 2021;137:643–51. 33190843 10.1016/j.jpsychires.2020.11.006

[64] Honea KE, Wilson KS, Fisher KL, Rubin DA. Parental and familial factors related to participation in a home-based physical activity intervention in children with obesity or Prader-Willi syndrome. Obes Pillars. 2023;8:100084. 38125663 10.1016/j.obpill.2023.100084 PMC10728700

[65] Ihara H, Ogata H, Sayama M, Kato A, Gito M, Murakami N, Kido Y, Nagai T. QOL in caregivers of Japanese patients with Prader-Willi syndrome with reference to age and genotype. Am J Med Genet A. 2014;164(9):2226–31. 10.1002/ajmg.a.36634 PMC4278419 24953026

[66] Kayadjanian N, Schwartz L, Farrar E, Comtois KA, Strong TV. High levels of caregiver burden in Prader-Willi syndrome. PLoS ONE. 2018;13(3):e0194655. 29579119 10.1371/journal.pone.0194655 PMC5868812

[67] Kendall KM, Rees E, Bracher-Smith M, Legge S, Riglin L, Zammit S, O’Donovan MC, Owen MJ, Jones I, Kirov G, Walters JTR. Association of rare copy number variants with risk of depression. JAMA Psychiat. 2019;76(8):818–25. 10.1001/jamapsychiatry.2019.0566 PMC6583866 30994872

[68] Kowal K, Skrzypek M, Kocki J. Experiencing illness as a crisis by the caregivers of individuals with Prader-Willi syndrome. PLoS ONE. 2022;17(9):e0273295. 36048794 10.1371/journal.pone.0273295 PMC9436047

[69] Krefft M, Frydecka D, Zalsman G, Krzystek-Korpacka M, Smigiel R, Gebura K, Bogunia-Kubik K, Misiak B. A pro-inflammatory phenotype is associated with behavioural traits in children with Prader-Willi syndrome. Eur Child Adolesc Psychiatry. 2021;30(6):899–908. 32495042 10.1007/s00787-020-01568-7 PMC8140962

[70] Lavelle TA, Crossnohere NL, Bridges JFP. Eliciting quality adjusted life years using the time trade off method for Prader-Willi syndrome. Patient. 2020;13(1):140.

[71] Lavelle TA, Crossnohere NL, Bridges JFP. Quantifying the burden of hyperphagia in Prader-Willi syndrome using quality-adjusted life-years. Clin Ther. 2021;43(7):1164-78.e4. 34193348 10.1016/j.clinthera.2021.05.013

[72] Lo ST, Siemensma EP, Festen DA, Collin PJ, Hokken-Koelega AC. Behavior in children with Prader-Willi syndrome before and during growth hormone treatment: a randomized controlled trial and 8-year longitudinal study. Eur Child Adolesc Psychiatry. 2015;24(9):1091–101. 25522840 10.1007/s00787-014-0662-4

[73] Lopez-Bastida J, Linertova R, Oliva-Moreno J, Posada-de-la-Paz M, Serrano-Aguilar P, Kanavos P, Taruscio D, Schieppati A, Iskrov G, Baji P, Delgado C, von der Schulenburg JMG, Persson U, Chevreul K, Fattore G. Social/economic costs and health-related quality of life in patients with Prader-Willi syndrome in Europe. Eur J Health Econ. 2016;17(Supplement 1):99–108. 27038627 10.1007/s10198-016-0788-z

[74] Maccarone MC, Avenia M, Masiero S. Postural-motor development, spinal range of movement and caregiver burden in Prader-Willi syndrome-associated scoliosis: an observational study. Eur J. 2024;34(2):22. 10.4081/ejtm.2024.12533 PMC11264224 38651535

[75] Mackay J, Nixon GM, Lafferty AR, Ambler G, Kapur N, Bergman PB, Schofield C, Seton C, Tai A, Tham E, Vora K, Crock P, Verge C, Musthaffa Y, Blecher G, Caudri D, Leonard H, Jacoby P, Wilson A, Choong CS, Downs J. Associations between hyperphagia, symptoms of sleep breathing disorder, behaviour difficulties and caregiver well-being in Prader-Willi syndrome: a preliminary study. J Autism Dev Disord. 2022;52(9):3877–89. 34498151 10.1007/s10803-021-05265-5

[76] Mao SJ, Shen J, Xu F, Zou CC. Quality of life in caregivers of young children with Prader-Willi syndrome. World J Pediatr. 2019;15(5):506–10. 31520366 10.1007/s12519-019-00311-w

[77] Mastey B-YH, Gross-Tsur V, Hirsch HJ, Genstil L, Derei D, Forer D, Benarroch F. Quality of life for adults with Prader-Willi syndrome in residential group homes. Journal. 2024;13(11):504. 10.3390/jcm13113323 PMC11173323 38893034

[78] Matesevac L, Vrana-Diaz CJ, Bohonowych JE, Schwartz L, Strong TV. Analysis of hyperphagia questionnaire for clinical trials (HQ-CT) scores in typically developing individuals and those with Prader-Willi syndrome. Sci. 2023;13(1):20573. 10.1038/s41598-023-48024-5 PMC10667498 37996659

[79] McCandless SE, Yanovski JA, Miller J, Fu C, Bird LM, Salehi P, et al. Effects of MetAP2 inhibition on hyperphagia and body weight in Prader-Willi syndrome: a randomized, double-blind, placebo-controlled trial. Diabetes Obes Metab. 2017;19(12):1751–61. 28556449 10.1111/dom.13021 PMC5673540

[80] McNulty BS, P. Kim, S. Kim, L. Howard, W. J. Aberrant Behavior Checklist Scores in Youth with Prader-Willi Syndrome. Pediatric Academic Societies Meeting 2023. 2023.

[81] Meade C, Martin R, Crowe C, Lyons J, McCrann A, Roche E. The impact of caring for a child with Prader Willi syndrome. Arch Dis Child. 2019;104(Supplement 2):A227.

[82] Meade C, Martin R, Lyons J, McCrann A, Roche E. Quality of life and the impact of caring for a child with Prader Willi syndrome. Arch Dis Child. 2019;104(Supplement 3):A88–9.

[83] Miller JL, Lacroix A, Bird LM, Shoemaker AH, Haqq A, Deal CL, Clark KA, Ames MH, Suico JG, de la Peña A, et al. The efficacy, safety, and pharmacology of a Ghrelin O-acyltransferase inhibitor for the treatment of Prader-Willi syndrome. J Clin Endocrinol Metab. 2022;107(6):e2373–80. 35213714 10.1210/clinem/dgac105 PMC10061054

[84] Miller JL, Gevers E, Bridges N, Yanovski JA, Salehi P, Obrynba KS, Felner EI, Bird LM, Shoemaker AH, Angulo M, Butler MG, Stevenson D, Abuzzahab J, Barrett T, Lah M, Littlejohn E, Mathew V, Cowen NM, Bhatnagar A. Diazoxide choline extended-release tablet in people with Prader-Willi syndrome: a double-blind, Placebo-Controlled trial. J Clin Endocrinol Metab. 2023;108(7):1676–85. 36639249 10.1210/clinem/dgad014 PMC10271219

[85] Miller JL, Gevers E, Bridges N, Yanovski JA, Salehi P, Obrynba KS, Felner EI, Bird LM, Shoemaker AH, Angulo M, Butler MG, Stevenson D, Goldstone AP, Wilding J, Lah M, Shaikh MG, Littlejohn E, Abuzzahab MJ, Fleischman A, Hirano P, Yen K, Cowen NM, Bhatnagar A. Diazoxide choline extended-release tablet in people with Prader-Willi syndrome: results from long-term open-label study. Obesity. 2024;32(2):252–61. 37919617 10.1002/oby.23928 PMC12181816

[86] Montes AS, Osann KE, Gold JA, Tamura RN, Driscoll DJ, Butler MG, Kimonis VE. Genetic subtype-phenotype analysis of growth hormone treatment on psychiatric behavior in Prader-Willi syndrome. Genes (Basel). 2020;11(11):23. 10.3390/genes11111250 PMC7690822 33114160

[87] ClinicalTrials.gov. Cannabidiol Oral Solution for the Treatment of Patients With Prader-Willi Syndrome NCT02844933 [Trial registry record]. 2016 [Available from: https://www.cochranelibrary.com/central/doi/10.1002/central/CN-01507046/full]. Last accessed Last accessed: 31 January 2025.

[88] O’Neill LP, Murray LE. Anxiety and depression symptomatology in adult siblings of individuals with different developmental disability diagnoses. Res Dev Disabil. 2016;51–52:116–25. 26820453 10.1016/j.ridd.2015.12.017

[89] Patel V, Davis K, Merikle E, McClure E, Patroneva A. PRO60 the epworth sleepiness scale for children and adolescents is a fit-for-purpose measure of daytime sleepiness in Prader-Willi syndrome: a qualitative interview study. Value in Health. 2021;24(Supplement 1):S208.

[90] Patel VP, Patroneva A, Glaze DG, Davis K, Merikle E, Revana A. Establishing the content validity of the epworth sleepiness scale for children and adolescents in Prader-Willi syndrome. J Clin Sleep Med. 2022;18(2):485–96. 34437052 10.5664/jcsm.9632 PMC8804999

[91] Pedemonti B, Ceccomancini R, D’Acunti A, Stegmann J. Effectiveness of a transdisciplinary approach on hyperphagia management among patients with Prader Willi syndrome. Endocrinol Diabetes Nutr (Engl Ed). 2023;70(5):347–51. 37263734 10.1016/j.endien.2022.11.021

[92] Peleggi A, Bohonowych J, Strong TV, Schwartz L, Kim SJ. Suicidality in individuals with Prader-Willi syndrome: a review of registry survey data. BMC Psychiatry. 2021;21(1):438. 34488710 10.1186/s12888-021-03436-3 PMC8422732

[93] Ragusa L, Crino A, Grugni G, Reale L, Fiorencis A, Licenziati MR, Faienza MF, Wasniewska M, Delvecchio M, Franzese A, Rutigliano I, Fusilli P, Corica D, Campana G, Greco D, Chiarito M, Sacco M, Toscano S, Marini MG. Caring and living with Prader-Willi syndrome in Italy: integrating children, adults and parents’ experiences through a multicentre narrative medicine research. BMJ Open. 2020;10(8):e036502. 32764084 10.1136/bmjopen-2019-036502 PMC7412587

[94] Reilly C, Murtagh L, Senior J. The impact on the family of four neurogenetic syndromes: a comparative study of parental views. J Genet Couns. 2015;24(5):851–61. 25597928 10.1007/s10897-015-9820-1

[95] Rice LJ, Gray KM, Howlin P, Taffe J, Tonge BJ, Einfeld SL. The developmental trajectory of disruptive behavior in Down syndrome, fragile X syndrome, Prader-Willi syndrome and Williams syndrome. Am J Med Genet C Semin Med Genet. 2015;169(2):182–7. 25983069 10.1002/ajmg.c.31442

[96] Rice LJ, Gray KM, Howlin P, Taffe J, Tonge BJ, Einfeld SL. The developmental trajectory of self-injurious behaviours in individuals with Prader Willi syndrome, autism spectrum disorder and intellectual disability. Diseases. 2016;4(1):06. 10.3390/diseases4010009 PMC5456304 28933389

[97] Roof E, Deal CL, McCandless SE, Cowan RL, Miller JL, Hamilton JK, Roeder ER, McCormack SE, Roshan LT, et al. Intranasal carbetocin reduces hyperphagia, anxiousness, and distress in Prader-Willi syndrome: CARE-PWS phase 3 trial. J Clin Endocrinol Metab. 2023;108(7):1696–708. 36633570 10.1210/clinem/dgad015 PMC10271225

[98] Royston R, Waite J, Howlin P, Dosse A, Armitage P, Moss J, Oliver C. Cross-syndrome comparison of psychopathological risk factors in williams syndrome, fragile x syndrome and prader-willi syndrome. J Intellect Disabil Res. 2018;62(8):670.

[99] Royston R, Oliver C, Howlin P, Dosse A, Armitage P, Moss J, Waite J. The profiles and correlates of psychopathology in adolescents and adults with Williams, Fragile X and Prader-Willi syndromes. J Autism Dev Disord. 2020;50(3):893–903. 31802317 10.1007/s10803-019-04317-1 PMC7010621

[100] Rozensztrauch A, Smigiel R. Quality of life in children with Prader-Willi syndrome and the impact of the disease on the functioning of families. Int J Environ Res Public Health. 2022;19(23):589. 36498413 10.3390/ijerph192316330 PMC9740001

[101] Rubin DA, Wilson KS, Castner DM, Dumont-Driscoll MC. Changes in health-related outcomes in youth with obesity in response to a home-based parent-led physical activity program. J Adolesc Health. 2019;65(3):323–30. 30833118 10.1016/j.jadohealth.2018.11.014

[102] Rubin DA, Wilson KS, Tucker JM, Castner DM, Dumont-Driscoll MC, Rose DJ. Improved motor proficiency and quality of life in youth with Prader-Willi syndrome and obesity 6 months after completing a parent-led, game-based intervention. Pediatr Exerc Sci. 2021;33(4):177–85. 34375948 10.1123/pes.2020-0160

[103] Schofield C, Martin KS, Choong C, Gibson D, Skoss R, Downs J. Using a trauma informed practice framework to enhance understanding of and identify support strategies for behavioural difficulties in young people with Prader-Willi syndrome. Res Dev Disabil. 2021;110:528. 10.1016/j.ridd.2020.103839 33482559

[104] Shivers CM, Leonczyk CL, Dykens EM. Life satisfaction among mothers of individuals with Prader-Willi syndrome. J Autism Dev Disord. 2016;46(6):2126–37. 26883647 10.1007/s10803-016-2741-5

[105] Shriki-Tal L, Avrahamy H, Pollak Y, Gross-Tsur V, Genstil L, Hirsch HJ, Benarroch F. Psychiatric disorders in a cohort of individuals with Prader-Willi syndrome. Eur Psychiatry. 2017;44:47–52. 28545008 10.1016/j.eurpsy.2017.03.007

[106] Strong TV, Miller JL, McCandless SE, Gevers E, Yanovski JA, Matesevac L, Bohonowych J, Ballal S, Yen K, Hirano P, Cowen NM, Bhatnagar A. Behavioral changes in patients with Prader-Willi syndrome receiving diazoxide choline extended-release tablets compared to the PATH for PWS natural history study. J Neurodev Disord. 2024;16(1):22. 38671361 10.1186/s11689-024-09536-x PMC11046911

[107] Thomson A, Glasson E, Roberts P, Bittles A. “Over time it just becomes easier…”: parents of people with Angelman syndrome and Prader-Willi syndrome speak about their carer role. Disabil Rehabil. 2017;39(8):763–70. 27015406 10.3109/09638288.2016.1161838

[108] Trueba-Timmermans DJ, Grootjen LN, Juriaans AF, Mahabier EF, Kerkhof GF, Rings EHHM, Hokken-Koelega ACS. Cognitive function during 3 years of growth hormone in previously growth hormone-treated young adults with Prader-Willi syndrome. Eur. 2023;189(1):132–9. 10.1093/ejendo/lvad084 37440711

[109] Tvrdik T, Mason D, Dent KM, Thornton L, Hornton SN, Viskochil DH, Stevenson DA. Stress and coping in parents of children with Prader-Willi syndrome: assessment of the impact of a structured plan of care. Am J Med Genet A. 2015;167A(5):974–82. 25755074 10.1002/ajmg.a.36971

[110] Vitale SA. Parent recommendations for family functioning with Prader-Willi syndrome: a rare genetic cause of childhood obesity. J Pediatr Nurs. 2016;31(1):47–54. 26684080 10.1016/j.pedn.2015.11.001

[111] Wieting J, Eberlein C, Bleich S, Frieling H, Deest M. Behavioural change in Prader-Willi syndrome during COVID-19 pandemic. J Intellect Disabil Res. 2021;65(7):609–16. 33754414 10.1111/jir.12831 PMC8251312

[112] Wieting J, Jahn K, Buchholz V, Lichtinghagen R, Bleich S, Eberlein CK, Deest M, Frieling H. Alteration of serum leptin and LEP/LEPR promoter methylation in Prader-Willi syndrome. medRxiv. 2021;15:852. 10.1016/j.psyneuen.2022.105857 35803048

[113] Wieting J, Jahn K, Buchholz V, Lichtinghagen R, Deest-Gaubatz S, Bleich S, Eberlein CK, Deest M, Frieling H. Alteration of serum leptin and LEP/LEPR promoter methylation in Prader-Willi syndrome. Psychoneuroendocrinology. 2022;143:105857. 35803048 10.1016/j.psyneuen.2022.105857

[114] Wieting J, Jahn K, Eberlein CK, Bleich S, Frieling H, Deest M. Hypomethylation of the dopamine transporter (DAT) gene promoter is associated with hyperphagia-related behavior in Prader-Willi syndrome: a case-control study. Behav Brain Res. 2023;450:114494. 37182741 10.1016/j.bbr.2023.114494

[115] Wilson KS, Wiersma LD, Rubin DA. Quality of life in children with Prader Willi Syndrome: parent and child reports. Res Dev Disabil. 2016;57:149–57. 27433979 10.1016/j.ridd.2016.06.016

[116] Wong SB, Wang TS, Tsai WH, Tzeng IS, Tsai LP. Parenting stress in families of children with Prader-Willi syndrome. Am J Med Genet A. 2021;185(1):83–9. 33043996 10.1002/ajmg.a.61915

[117] Yamada K, Watanabe M, Suzuki K. Differential volume reductions in the subcortical, limbic, and brainstem structures associated with behavior in Prader-Willi syndrome. Sciences. 2022;12(1):4978. 10.1038/s41598-022-08898-3 PMC8943009 35322075

[118] ClinicalTrials.gov. Oxytocin vs. Placebo for the Treatment Hyperphagia in Children and Adolescents With Prader-Willi Syndrome NCT02629991 [Trial registry record]. 2015 [Available from: https://www.cochranelibrary.com/central/doi/10.1002/central/CN-01554390/full]. Last accessed Last accessed: 31 January 2025.

[119] Taffe JR, Gray KM, Einfeld SL, Dekker MC, Koot HM, Emerson E, et al. Short form of the developmental behaviour checklist. Am J Ment Retard. 2007;112(1):31–9. 17181389 10.1352/0895-8017(2007)112[31:SFOTDB]2.0.CO;2 PMC2424022

[120] Varni JW. The PedsQL Measurement Model for the Pediatric Quality of Life Inventory: PedsMetrics; [Available from: https://www.pedsql.org/about_pedsql.html]. Last accessed Last accessed: 10 January 2025.

[121] Huang IC, Thompson LA, Chi YY, Knapp CA, Revicki DA, Seid M, et al. The linkage between pediatric quality of life and health conditions: establishing clinically meaningful cutoff scores for the PedsQL. Value Health. 2009;12(5):773–81. 19508660 10.1111/j.1524-4733.2008.00487.x PMC4299816

[122] Devlin N, Parkin D, Janssen B. Methods for Analysing and Reporting EQ-5D Data. 1st ed. Springer; 2020. 33347096

[123] RAND. 36-Item Short Form Survey (SF-36): RAND; [Available from: https://www.rand.org/health-care/surveys_tools/mos/36-item-short-form.html]. Last accessed Last accessed: 10 January 2025.

[124] Mao S, Shen J, Yang L, Yang R, Zou C, Zhao Z. Quality of life in caregivers of young children with Prader-Willi syndrome. Hormone Res Paediatr. 2019;91(Supplement 1):172–3.

[125] Miller JL, Lynn CH, Driscoll DC, Goldstone AP, Gold JA, Kimonis V, et al. Nutritional phases in Prader-Willi syndrome. Am J Med Genet A. 2011;155A(5):1040–9. 21465655 10.1002/ajmg.a.33951 PMC3285445

[126] Baker EK, Arora S, Amor DJ, Date P, Cross M, O’Brien J, Simons C, Rogers C, Goodall S, Slee J, Cahir C, Godler DE. The cost of raising individuals with Fragile X or chromosome 15 imprinting disorders in Australia. J Autism Dev Disord. 2023;53(4):1682–92. 34292487 10.1007/s10803-021-05193-4

[127] Bar C, Diene G, Molinas C, Bieth E, Casper C, Tauber M. Early diagnosis and care is achieved but should be improved in infants with Prader-Willi syndrome. Orphanet J Rare Dis. 2017;12(1):118. 28659150 10.1186/s13023-017-0673-6 PMC5490212

[128] Butler MG, Manzardo A, Strong TV, Li JW, Yin D, Meng Q, Silber A, Francis K, Hadker N, Czado S, Yeh M, Miller JL. Pro22 cost of care analysis for U.S. patients with Prader-Willi syndrome (Pws). Value in Health. 2020;23:5332.

[129] Chung AS, Renfree S, Lockwood DB, Karlen J, Belthur M. Syndromic scoliosis: national trends in surgical management and inpatient hospital outcomes: a 12-year analysis. Spine. 2019;44(22):1564–70. 31689252 10.1097/BRS.0000000000003134

[130] Clerc A, Coupaye M, Mosbah H, Pinto G, Laurier V, Mourre F, Merrien C, Diene G, Poitou C, Tauber M. Diabetes mellitus in prader-willi syndrome: natural history during the transition from childhood to adulthood in a cohort of 39 patients. Journal. 2021;10(22):15. 10.3390/jcm10225310 PMC8625265 34830599

[131] Elliott JP, Cherpes G, Kamal K, Chopra I, Harrison C, Riedy M, Herk B, McCrossin M, Kalarchian M. Relationship between antipsychotics and weight in patients with Prader-Willi syndrome. Pharmacotherapy. 2015;35(3):260–8. 25809177 10.1002/phar.1558

[132] Gul Y, Kapakli H, Aytekin SE, Guner SN, Keles S, Zamani AG, Yildirim MS, Reisli I. Evaluation of immunological abnormalities in patients with rare syndromes. Central Eur J Urol. 2022;47(4):299–307. 10.5114/ceji.2022.124080 PMC9901257 36817395

[133] Hughes BM, Holland A, Hodebeck-Stuntebeck N, Garrick L, Goldstone AP, Lister M, Moore C, Hughes M. Body weight, behaviours of concern, and social contact in adults and adolescents with Prader-Willi syndrome in full-time care services: findings from pooled international archival data. Orphanet J Rare Dis. 2024;19(1):48. 38326873 10.1186/s13023-024-03035-x PMC10848374

[134] Laurier V, Lapeyrade A, Copet P, Demeer G, Silvie M, Bieth E, Coupaye M, Poitou C, Lorenzini F, Labrousse F, Molinas C, Tauber M, Thuilleaux D, Jauregi J. Medical, psychological and social features in a large cohort of adults with Prader-Willi syndrome: experience from a dedicated centre in France. J Intellect Disabil Res. 2015;59(5):411–21. 24947991 10.1111/jir.12140

[135] McQuivey KS, Sheridan JR, Chung A, Mayfield C, Gulbrandsen M, Brinkman JC, Belthur MV. Hospital outcomes of scoliosis surgery in children with Prader-Willi Syndrome: comparison with adolescent idiopathic scoliosis. Spine Deformity. 2021;9(6):1641–7. 33950464 10.1007/s43390-021-00359-7

[136] McQuivey KS, Chung AS, Jones MR, Makovicka JL, Christopher ZK, Brinkman JC, Belthur M. Hospital outcomes in pediatric patients with Prader-Willi syndrome (PWS) undergoing orthopedic surgery: a 12-year analysis of national trends in surgical management and inpatient hospital outcomes. J Orthop Sci. 2022;27(6):1304–8. 34531085 10.1016/j.jos.2021.08.005

[137] Meade C, Martin R, McCrann A, Lyons J, Roche E. Growth and nutritional status in children with Prader Willi syndrome. Arch Dis Child. 2019;104(Supplement 3):A117–8.

[138] Pellikaan K, Snijders F, Rosenberg AGW, Davidse K, van Berg SAA, Visser WE, et al. Thyroid function in adults with prader-willi syndrome; a cohort study and literature review. Journal. 2021;10(17):25. 10.3390/jcm10173804 PMC8432005 34501256

[139] Pemmasani G, Yandrapalli S. Age-stratified prevalence of relevant comorbidities and etiologies for hospitalizations in Prader-Willi syndrome patients. Am J Med Genet A. 2021;185(2):600–1. 33179418 10.1002/ajmg.a.61968

[140] Potluri R, Ranjan S, Khurana R, Lele AM, Prabhakar V, Bhandari H. Evaluation of average cost-effectiveness ratios of standards of care across different indications. Value Health. 2015;18(7):A575.

[141] Prapasrat C, Onsod P, Korkiatsakul V, Rerkamnuaychoke B, Wattanasirichaigoon D, Chareonsirisuthigul T. The utilization of MS-MLPA as the first-line test for the diagnosis of prader-willi syndrome in thai patients. J Pediatr Genet. 2021;12(4):273–9. 10.1055/s-0041-1741008 PMC10756717 38162164

[142] Sequeira AR, Mentzakis E, Archangelidi O, Paolucci F. The economic and health impact of rare diseases: a meta-analysis. Health Policy Technol. 2021;10(1):32–44.

[143] Shepherd S, Saraff V, Shaw N, Banerjee I, Patel L. Growth hormone prescribing patterns in the UK, 2013–2016. Arch Dis Child. 2019;104(6):583–7. 30567827 10.1136/archdischild-2018-316262

[144] Shoffstall AJ, Gaebler JA, Kreher NC, Niecko T, Douglas D, Strong TV, Miller JL, Stafford DE, Butler MG. The high direct medical costs of prader-willi syndrome. J Pediatr. 2016;175:137–43. 27283463 10.1016/j.jpeds.2016.05.018 PMC7464637

[145] Van Bosse HJP, Gantz MG, Ong KL, Cox JB. Comparison of Hip and Knee arthroplasty rates of individuals with and without prader-willi syndrome. J Pediatric Orthop. 2020;40(5):E362–6. 10.1097/BPO.0000000000001490 31834241

[146] Yang L, Zou C. Perinatal features of prader-willi syndrome: a Chinese cohort. Hormone Res Paediatr. 2019;91(Supplement 1):300.

[147] Yang L, Zhou Q, Ma B, Mao S, Dai Y, Zhu M, Zou C. Perinatal features of Prader-Willi syndrome: a Chinese cohort of 134 patients. Orphanet J Rare Dis. 2020;15(1):24. 31964399 10.1186/s13023-020-1306-z PMC6975078

[148] Heymsfield SB, Clement K, Dubern B, Goldstone AP, Haqq AM, Kuhnen P, et al. Defining hyperphagia for improved diagnosis and management of MC4R pathway-associated disease: a roundtable summary. Curr Obes Rep. 2025;14(1):13. 39856371 10.1007/s13679-024-00601-z PMC11762201

[149] Bellis SA, Kuhn I, Adams S, Mullarkey L, Holland A. The consequences of hyperphagia in people with Prader-Willi Syndrome: a systematic review of studies of morbidity and mortality. Eur J Med Genet. 2022;65(1):104379. 34748997 10.1016/j.ejmg.2021.104379

