# Supplementary material for: The burden of illness in Prader-Willi syndrome: a systematic literature review
Source: Orphanet J Rare Dis. 2025 Jul 24;20:374. doi: 10.1186/s13023-025-03787-0 (PMC12291511; doi:10.1186/s13023-025-03787-0)
Supplement: Supplementary file 1 — Additional file 1: Search strategy. [file 13023_2025_3787_MOESM1_ESM.docx]

Supplemental Table 1 mortality search strategy

| **Database** | **Number** | **Search terms** | **Hits 13Aug24** |
| --- | --- | --- | --- |
| MEDLINE | 1 | exp Prader-Willi Syndrome/ or ((Prader adj2 Willi*) or (Labhart adj2 Willi*) or (Prader adj2 Labhart)).ti,ab. | 4702 |
|  | 2 | Exp mortality/ or (mortal* or death or die or died or fatal*).ab,ti. | 2335820 |
|  | 3 | 1 and 2 | 246 |
|  | 4 | limit 3 to dt=20140801-20240831 | 108 |
| Embase | 1 | exp Prader-Willi Syndrome/ or ((Prader adj2 Willi*) or (Labhart adj2 Willi*) or (Prader adj2 Labhart)).ti,ab. | 7673 |
|  | 2 | Exp mortality/ or (mortal* or death or die or died or fatal*).ab,ti. | 3436772 |
|  | 3 | 1 and 2 | 464 |
|  | 4 | limit 3 to dc=20140801-20240831 | 254 |
| Cochrane Library | 1 | [mh "Prader-Willi Syndrome"] or ((Prader NEAR/2 Willi*) or (Labhart NEAR/2 Willi*) or (Prader NEAR/2 Labhart)):ti,ab | 274 |
|  | 2 | [mh "mortality"] or (mortal* or death or die or died or fatal*):ab,ti | 171456 |
|  | 3 | #1 and #2 | 15 |
|  | 4 | with Cochrane Library publication date from Aug 2014 to Aug 2024 | 12 |

Supplemental Table 2 humanistic burden search strategy

| **Database** | **Number** | **Search terms** | **Hits 13Aug2024** |
| --- | --- | --- | --- |
| MEDLINE | 1 | exp Prader-Willi Syndrome/ or ((Prader adj2 Willi*) or (Labhart adj2 Willi*) or (Prader adj2 Labhart)).ti,ab. | 4702 |
|  | 2 | exp quality of life/ or exp quality adjusted life years/ or exp health surveys/ or Value of Life/ or exp Disability Evaluation/ or exp models, economic/ or exp questionnaire/ or exp visual analog scale/ | 1514852 |
|  | 3 | (quality of life or utilit* or quality adjusted or adjusted life or qaly* or qald* or qale* or qtime* or life year or life years or disability adjusted life or daly* or short form* or shortform* or sf* or hql or qol or hrql or hqol or h qol or hrqol or hr qol or hye or hyes or (health* adj2 year* adj2 equivalent*) or pqol or qls or quality of wellbeing or quality of well being or index of wellbeing or index of well being or qwb or nottingham health profile* or sickness impact profile or ((health or illness) adj3 stat*) or (preference* adj3 (score* or scoring or valu* or measur* or evaluat* or scale* or instrument* or weight or weights or weighting or information or data or unit or units or health* or life or estimat* or elicit* or disease* or mean or cost* or expenditure* or gain or gains or loss or losses or lost or analysis or index* or indices or overall or reported or calculat* or range* or increment* or state or states or status)) or disutilit* or HSUV or HSUVs or rosser or willingness to pay or standard gamble* or sg or time trade off or time tradeoff or timetradeoff or tto or hui or hui1 or hui2 or hui3 or eq or euroqol* or euro qol* or eq5d or eq-5d or eq5-d or euroqual* or euro qual* or eq-sdq or eqsdq or duke health profile or functional status questionnaire or dartmouth coop functional health assessment* or multiattribute* or multi attribute* or 15D or 15-D or 15 dimension or medical outcome study or RAND36 or RAND12 or (health adj3 (status or index)) or PedsQL or Visual analog* scale or VAS or Fatigue Assessment Scale or FAS or caregiver* or carer or burden or Administrat* or ((injection or treat*) adj3 (daily or schedule or burden)) or patient-reported or life satisfaction or family function* or psychological health or mental health or depress* or suicid* or post-traumatic stress disorder or PTSD or Zarit Burden Interview or ZBI or Prader-Willi Syndrome Profile or PWSP or Hyperphagia Questionnaire for Clinical trials or HQCT or (PWS Anxiousness and Distress Behaviors Questionnaire) or PADQ or CSSRS or C-SSRS or Columbia* suicide severity rating scale or food safe* zone or FSZ or (Dykens adj3 hyperphag*) or DHQ or Developmental Behaviour Checklist* or DBC or DBC-2 or (risk adj2 tolerance)).ti,ab. | 3264360 |
|  | 4 | 2 or 3 | 4259753 |
|  | 5 | 1 and 4 | 666 |
|  | 6 | limit 5 to dt=20140801-20240831 | 336 |
| Embase | 1 | exp Prader-Willi Syndrome/ or ((Prader adj2 Willi*) or (Labhart adj2 Willi*) or (Prader adj2 Labhart)).ti,ab. | 7673 |
|  | 2 | socioeconomics/ or exp Quality of Life/ or exp Quality-Adjusted Life Year/ or nottingham health profile/ or sickness impact profile/ or exp health survey/ or exp Disability Evaluation/ or exp models, economic/ or exp questionnaire/ or exp visual analog scale/ or Columbia suicide severity rating scale/ | 2069807 |
|  | 3 | (quality of life or utilit* or quality adjusted or adjusted life or qaly* or qald* or qale* or qtime* or life year or life years or disability adjusted life or daly* or short form* or shortform* or sf* or hql or qol or hrql or hqol or h qol or hrqol or hr qol or hye or hyes or (health* adj2 year* adj2 equivalent*) or pqol or qls or quality of wellbeing or quality of well being or index of wellbeing or index of well being or qwb or nottingham health profile* or sickness impact profile or ((health or illness) adj3 stat*) or (preference* adj3 (score* or scoring or valu* or measur* or evaluat* or scale* or instrument* or weight or weights or weighting or information or data or unit or units or health* or life or estimat* or elicit* or disease* or mean or cost* or expenditure* or gain or gains or loss or losses or lost or analysis or index* or indices or overall or reported or calculat* or range* or increment* or state or states or status)) or disutilit* or HSUV or HSUVs or rosser or willingness to pay or standard gamble* or sg or time trade off or time tradeoff or timetradeoff or tto or hui or hui1 or hui2 or hui3 or eq or euroqol* or euro qol* or eq5d or eq-5d or eq5-d or euroqual* or euro qual* or eq-sdq or eqsdq or duke health profile or functional status questionnaire or dartmouth coop functional health assessment* or multiattribute* or multi attribute* or 15D or 15-D or 15 dimension or medical outcome study or RAND36 or RAND12 or (health adj3 (status or index)) or PedsQL or Visual analog* scale or VAS or Fatigue Assessment Scale or FAS or caregiver* or carer or burden or Administrat* or ((injection or treat*) adj3 (daily or schedule or burden)) or patient-reported or life satisfaction or family function* or psychological health or mental health or depress* or suicid* or post-traumatic stress disorder or PTSD or Zarit Burden Interview or ZBI or Prader-Willi Syndrome Profile or PWSP or Hyperphagia Questionnaire for Clinical trials or HQCT or (PWS Anxiousness and Distress Behaviors Questionnaire) or PADQ or CSSRS or C-SSRS or Columbia* suicide severity rating scale or food safe* zone or FSZ or (Dykens adj3 hyperphag*) or DHQ or Developmental Behaviour Checklist* or DBC or DBC-2 or (risk adj2 tolerance)).ti,ab. | 4479589 |
|  | 4 | 2 or 3 | 5620004 |
|  | 5 | 1 and 4 | 1133 |
|  | 6 | limit 5 to dc=20140801-20240831 | 689 |
| Cochrane Library | 1 | [mh "Prader-Willi Syndrome"] or ((Prader NEAR/2 Willi*) or (Labhart NEAR/2 Willi*) or (Prader NEAR/2 Labhart)):ti,ab | 274 |
|  | 2 | [mh "quality of life"] or [mh "quality adjusted life years"] or [mh "health surveys"] or [mh "Value of Life"] or [mh "Disability Evaluation"] or [mh "models, economic"] or [mh "questionnaire"] or [mh "visual analog scale"] | 116102 |
|  | 3 | (quality of life or utilit* or quality adjusted or adjusted life or qaly* or qald* or qale* or qtime* or life year or life years or disability adjusted life or daly* or short form* or shortform* or sf* or hql or qol or hrql or hqol or h qol or hrqol or hr qol or hye or hyes or (health* NEAR/2 year* NEAR/2 equivalent*) or pqol or qls or quality of wellbeing or quality of well being or index of wellbeing or index of well being or qwb or nottingham health profile* or sickness impact profile or ((health or illness) NEAR/3 stat*) or (preference* NEAR/3 (score* or scoring or valu* or measur* or evaluat* or scale* or instrument* or weight or weights or weighting or information or data or unit or units or health* or life or estimat* or elicit* or disease* or mean or cost* or expenditure* or gain or gains or loss or losses or lost or analysis or index* or indices or overall or reported or calculat* or range* or increment* or state or states or status)) or disutilit* or HSUV or HSUVs or rosser or willingness to pay or standard gamble* or sg or time trade off or time tradeoff or timetradeoff or tto or hui or "hui1" or "hui2" or "hui3" or eq or euroqol* or euro qol* or "eq5d" or "eq-5d" or "eq5-d" or euroqual* or euro qual* or eq-sdq or eqsdq or duke health profile or functional status questionnaire or dartmouth coop functional health assessment* or multiattribute* or multi attribute* or "15D" or "15-D" or "15 dimension" or medical outcome study or "RAND36" or "RAND12" or (health NEAR/3 (status or index)) or PedsQL or Visual analog* scale or VAS or Fatigue Assessment Scale or FAS or caregiver* or carer or burden or Administrat* or ((injection or treat*) NEAR/3 (daily or schedule or burden)) or patient-reported or life satisfaction or family function* or psychological health or mental health or depress* or suicid* or post-traumatic stress disorder or PTSD or Zarit Burden Interview or ZBI or Prader-Willi Syndrome Profile or PWSP or Hyperphagia Questionnaire for Clinical trials or HQCT or "PWS Anxiousness and Distress Behaviors Questionnaire" or PADQ or CSSRS or C-SSRS or "Columbia suicide severity rating scale" or "food safe zone" or "food safety zone" or FSZ or (Dykens adj3 hyperphagia) or DHQ or "Developmental Behaviour Checklist" or DBC or DBC-2 or (risk adj2 tolerance)):ti,ab | 631989 |
|  | 4 | #2 OR #3 | 681902 |
|  | 5 | #1 AND #4 | 144 |
|  | 6 | with Cochrane Library publication date from Aug 2014 to Aug 2024 | 122 |
| Centre for Reviews and Dissemination Database | 1 | Prader-Willi Syndrome | 15 |
| EconLit | 1 | ((Prader adj2 Willi*) or (Labhart adj2 Willi*) or (Prader adj2 Labhart)).mp. | 1 |

Supplemental Table 3 economic burden search strategy

| **Database** | **Number** | **Search terms** | **Hits 13Aug2024** |
| --- | --- | --- | --- |
| MEDLINE | 1 | exp Prader-Willi Syndrome/ or ((Prader adj2 Willi*) or (Labhart adj2 Willi*) or (Prader adj2 Labhart)).ti,ab. | 4702 |
|  | 2 | Economics/ or exp "costs and cost analysis"/ or Value of life/ or exp economics, hospital/ or exp economics, medical/ or Economics, nursing/ or Economics, pharmaceutical/ or exp "fees and charges"/ or exp budgets/ or exp models, economic/ or Income/ or Remuneration/ or "Salaries and Fringe Benefits"/ or health resources/ or health planning/ or insurance, disability/ or insurance, health, reimbursement/ or drug utilization/ or technology assessment, biomedical/ or employment/ or work/ or absenteeism/ or efficiency/ or presenteeism/ | 537882 |
|  | 3 | (Economic* or pharmacoeconomic* or cost* or resource* or fiscal or funding or financial or finance* or price* or pricing or hospitalisation* or hospitalization* or stay* or office visits or (A&E adj2 visit) or ((A adj2 E) and visit) or ("accident and emergency" and visit) or mental health service* or "medication use" or expenditure* or budget* or expens* or earning* or salar* or wage* or pay or pays or paid or paying or payment* or income* or remunerat* or money or monetary or fee or fees or charg* or productiv* or (burden adj2 (illness or disease* or health or global)) or "societal impact" or "social impact" or employment or employed or employee* or unemploy* or "sick leave" or (productivity adj2 (cost* or loss*)) or ((carer or caregiver) adj2 burden)).ab,ti. | 3516668 |
|  | 4 | 2 OR 3 | 3726868 |
|  | 5 | 1 AND 4 | 334 |
|  | 6 | limit 5 to dt=20140801-20240831 | 162 |
| Embase | 1 | exp Prader-Willi Syndrome/ or ((Prader adj2 Willi*) or (Labhart adj2 Willi*) or (Prader adj2 Labhart)).ti,ab. | 7673 |
|  | 2 | Socioeconomics/ or Cost benefit analysis/ or Cost effectiveness analysis/ or Cost of illness/ or Cost control/ or Economic aspect/ or Financial management/ or Health care cost/ or Health care financing/ or Health economics/ or Hospital cost/ or Cost minimization analysis/ or exp Economics/ or cost/ or drug cost/ or pharmacoeconomics/ or exp fee/ or budget/ or Economic Evaluation/ or cost utility analysis/ or hospitalization cost/ or nursing cost/ or health care planning/ or drug utilization/ or health insurance/ or biomedical technology assessment/ or employment/ or absenteeism/ or productivity/ or presenteeism/ or return to work/ | 1525770 |
|  | 3 | (Economic* or pharmacoeconomic* or cost* or resource* or fiscal or funding or financial or finance* or price* or pricing or hospitalisation* or hospitalization* or stay* or office visits or (A&E adj2 visit) or ((A adj2 E) and visit) or ("accident and emergency" and visit) or mental health service* or "medication use" or expenditure* or budget* or expens* or earning* or salar* or wage* or pay or pays or paid or paying or payment* or income* or remunerat* or money or monetary or fee or fees or charg* or productiv* or (burden adj2 (illness or disease* or health or global)) or "societal impact" or "social impact" or employment or employed or employee* or unemploy* or "sick leave" or (productivity adj2 (cost* or loss*)) or ((carer or caregiver) adj2 burden)).ab,ti. | 4537484 |
|  | 4 | 2 OR 3 | 5235182 |
|  | 5 | 1 AND 4 | 671 |
|  | 6 | limit 5 to dc=20140801-20240831 | 362 |
| Cochrane Library | 1 | [mh "Prader-Willi Syndrome"] or ((Prader NEAR/2 Willi*) or (Labhart NEAR/2 Willi*) or (Prader NEAR/2 Labhart)):ti,ab | 274 |
|  | 2 | [mh "Economics"] or [mh "costs and cost analysis"] or [mh "Value of life"] or [mh "economics, hospital"] or [mh "economics, medical"] or [mh "Economics, nursing"] or [mh "Economics, pharmaceutical"] or [mh "fees and charges"] or [mh "budgets"] or [mh "models, economic"] or [mh "Income"] or [mh "Remuneration"] or [mh "Salaries and Fringe Benefits"] or [mh "health resources"] or [mh "health planning"] or [mh "insurance, disability"] or [mh "insurance, health, reimbursement"] or [mh "drug utilization"] or [mh "technology assessment, biomedical"] or [mh "employment"] or [mh "work"] or [mh "absenteeism"] or [mh "efficiency"] or [mh "presenteeism"] | 31198 |
|  | 3 | (Economic* or pharmacoeconomic* or cost* or resource* or fiscal or funding or financial or finance* or price* or pricing or hospitalisation* or hospitalization* or stay* or office visits or (A&E NEAR/2 visit) or ((A NEAR/2 E) and visit) or ("accident and emergency" and visit) or mental health service* or "medication use" or expenditure* or budget* or expens* or earning* or salar* or wage* or pay or pays or paid or paying or payment* or income* or remunerat* or money or monetary or fee or fees or charg* or productiv* or (burden NEAR/2 (illness or disease* or health or global)) or "societal impact" or "social impact" or employment or employed or employee* or unemploy* or "sick leave" or (productivity NEAR/2 (cost* or loss*)) or ((carer or caregiver) NEAR/2 burden)):ti,ab | 284963 |
|  | 4 | #2 OR #3 | 293907 |
|  | 5 | #1 AND #4 | 35 |
|  | 6 | with Cochrane Library publication date from Aug 2014 to Aug 2024 | 23 |
| Centre for Reviews and Dissemination Database | 1 | Prader-Willi Syndrome | 15 |
| EconLit | 1 | ((Prader adj2 Willi*) or (Labhart adj2 Willi*) or (Prader adj2 Labhart)).mp. | 1 |

Supplemental Table 4 grey literature search strategy

| Number | Access | Search strategy | Included grey literature publications |
| --- | --- | --- | --- |
| ISPOR (all meetings) | <https://www.ispor.org/heor-resources/presentations-database/search> | Searched for:   - Prader-Willi: 13 - Prader Willi: 13 | Economic: 1  Bridges et al. Assessing the potential impact of treating hyperphagia among people with prader-willi syndrome using disease-specific qalys. Value in Health. 2018;21(Supplement 1):S256. |
| Children’s Hospital of Orange County (CHOC) and University of California, Irvine (UCI) | CHOC and UCI: Rare Disease Symposium & Family Conference <https://web.cvent.com/event/9747c55b-177c-49f1-be2a-253e111962a6/summary> | Unable to find abstract book: | Unable to find abstract book |
| Pediatric Endocrine Society (PES) Annual Meeting | 2024: Not yet available as of Jul16 (Annual meeting date: May 2-5 2024)  2023: <https://karger.com/hrp/article-pdf/96/Suppl.%203/1/4008437/000531602.pdf>  2022: <https://karger.com/hrp/article-pdf/95/Suppl.%201/1/4008149/000525242.pdf>  2021: <https://karger.com/hrp/article-pdf/94/Suppl.%202/1/4003812/000519117.pdf> | Searched for:  2023   - Prader-Willi: 4 - Prader Willi: 1   2022   - Prader-Willi: 3 - Prader Willi: 2   2021   - Prader-Willi: 1 - Prader Willi: 1 | 0 included |
| Pediatric Academic Societies (PAS) Meeting | 2024: <https://2024.pas-meeting.org/searchGlobal.asp>  2023: <https://2023.pas-meeting.org/searchGlobal.asp>  2022: <https://2022.pas-meeting.org/searchGlobal.asp>  2021: <https://virtual2021.pas-meeting.org/searchGlobal.asp> | Searched for:  2024:   - Prader-Willi: 0 - Prader Willi: 0   2023:   - Prader-Willi: 1 - Prader Willi: 0   2022:   - Prader-Willi: 1 - Prader Willi: 1   2021:   - Prader-Willi: 4 - Prader Willi: 0 | Humanistic: 1  McNulty BS, P. Kim, S. Kim, L. Howard, W. J. Aberrant Behavior Checklist Scores in Youth with Prader-Willi Syndrome. Pediatric Academic Societies Meeting. 2023. Available from: https://2023.pas-meeting.org/fsPopup.asp?PosterID=562067&mode=posterinfo |
| American Association of Clinical Endocrinology (AACE) | 2024: <https://www.sciencedirect.com/journal/endocrine-practice/vol/30/issue/5/suppl/S>  2023: <https://www.sciencedirect.com/journal/endocrine-practice/vol/29/issue/5/suppl/S>  2022: <https://www.sciencedirect.com/journal/endocrine-practice/vol/28/issue/5/suppl/S>  2021: <https://www.sciencedirect.com/journal/endocrine-practice/vol/27/issue/6/suppl/S> | Searched for:  2024:   - Prader-Willi: 1 - Prader Willi: 0   2023:   - Prader-Willi: 0 - Prader Willi: 0   2022:   - Prader-Willi: 0 - Prader Willi: 0   2021:   - Prader-Willi: 0 - Prader Willi: 0 | 0 included |
| Endocrine Society | 2024: not yet available as of Jul16 (Annual meeting date: June 1-4)  2023: <https://academic.oup.com/jes/issue/7/Supplement_1>  2022: <https://academic.oup.com/jes/issue/6/Supplement_1?login=false>  2021: <https://academic.oup.com/jes/issue/5/Supplement_1?login=false> | Searched for:  2023:   - Prader-Willi: 0 - Prader Willi: 0   2022:   - Prader-Willi: 5 - Prader Willi: 0   2021:   - Prader-Willi: 4 - Prader Willi: 1 | 0 included |
| Endocrine Nurses Society (ENS) | Conference: <https://www.endo-nurses.org/> | Unable to find abstract book | Unable to find abstract book |
| European Society for Paediatric Endocrinology (ESPE) | 2024: Not available at time of search (Annual meeting date: Nov 16-18)  2023: <https://karger.com/hrp/article/96/Suppl.%204/1/863132/Abstracts-of-the-ESPE-61-Annual-Society-Meeting?searchresult=1>  2022: <https://karger.com/hrp/article/95/Suppl.%202/1/823997/60th-Annual-Meeting-of-the-European-Society-for>  2021: <https://karger.com/hrp/article/94/Suppl.%201/1/823910/Abstracts?searchresult=1> | Searched for:  2023:   - Prader-Willi: 26 - Prader Willi: 4   2022:   - Prader-Willi: 15 - Prader Willi: 6   2021:   - Prader-Willi: 15 - Prader Willi: 0 | Humanistic: 1  Faye et al. The evolution of diagnosis and care over time in children with Prader-Willi syndrome, born between 2005 and 2021, included in the French database. European Society for Paediatric Endocrinology meeting 2023. Available from: https://abstracts.eurospe.org/hrp/0097/hrp0097p1-446 |
| European Society of Endocrinology (ECE) | 2024: <https://www.endocrine-abstracts.org/ea/0099/abstract-book/>  2023: <https://www.endocrine-abstracts.org/ea/0090/abstract-book/>  2022: <https://www.endocrine-abstracts.org/ea/0081>  2021: <https://www.endocrine-abstracts.org/ea/0073/abstracts/> | Searched for:  2024:   - Prader-Willi: 1 - Prader Willi: 2   2023:   - Prader-Willi: 3 - Prader Willi: 0   2022:   - Prader-Willi: 5 - Prader Willi: 2   2021:   - Prader-Willi: 4 - Prader Willi: 1 | 0 included |
| SLR handsearch | Database SLR publications | All excluded either for the date limit or against other eligibility criteria, being a duplicate against database results or each other. | 0 included |
