# Supplementary material for: The burden of illness in Prader-Willi syndrome: a systematic literature review
Source: Orphanet J Rare Dis. 2025 Jul 24;20:374. doi: 10.1186/s13023-025-03787-0 (PMC12291511; doi:10.1186/s13023-025-03787-0)
Supplement: Supplementary file 2 — Additional file 2: Table of included studies, table of QoL scales. [file 13023_2025_3787_MOESM2_ESM.docx]

Supplemental table 5 included studies table

| **Study reference** | **Study design** | **Country** | **Number of patients, N** | **Population** | **Patient characteristics: Age, years, mean (SD; median [range]); Female %** | **Time period** | **Outcome** |
| --- | --- | --- | --- | --- | --- | --- | --- |
| **Mortality** | | | | | | | |
| Avram et al. (2018) (1) | Retrospective cohort study of pregnancies | USA | 2,029,004 (PWS: 39) | PWS patients  Non-PWS patients | NR; NR | NR | Mortality rate  Cause of death |
| Bourke et al. (2018) (2) | Validation study using database data | Australia | 488,905 (PWS: 23) | Intellectual disability patients | NR; NR | 1983 to 2010 (27 years) | Mortality rate |
| Butler et al. (2017) (3) | Descriptive analysis of mortality data | USA | 486 | PWS patients | NR; 45% | 1973 to 2015 (42 years) | Life expectancy  Cause of death |
| Butler et al. (2018) (4) | Multicenter, longitudinal, observational, natural history study | USA | 391 (PWS: 355) | PWS patients  Early-onset morbid obesity | PWS patients: 13.4 (12.0; NR [NR]); 55.5%  Early-onset morbid obesity: 10.0 (4.8; NR [NR]); 50% | 07 September 2006 to 31 July 2014 (7 years 10 months) | Mortality rate  Cause of death |
| Hedgeman et al. (2017) (5) | Population-based cohort study | Denmark | 15,655 (PWS: 155) | PWS patients  General population | PWS patients: 18 (17; NR [NR]); 54.2%  General population: 18 (17; NR [NR]); 54.2% | 01 January 1977 to 31 December 2012 (36 years) | Mortality rate  Risk of death |
| Manzardo et al. (2018) (6) | Retrospective cohort study | USA | 486 | PWS patients | NR; 45% | 1975 to 2000 (25 years) and 2000-2015 (15 years) | Cause of death  Risk of death |
| McCandless et al. (2020) (7) | Medical claims data study | USA | 8,870 | PWS patients | NR (NR; 21 [NR]); NR | 2012 to 2014 (2 years) | Mortality rate  Life expectancy |
| Pacoricona et al. (2019) (8) | Retrospective observational study | France | 104 | Pediatric PWS patients  Adult PWS patients | Pediatric PWS patients: NR; 59%  Adult PWS patients: NR; 44% | 2004 to 2014 (10 years) | Life expectancy  Cause of death |
| Proffit et al. (2019) (9) | Questionnaire based study | USA | 2,029 | PWS patients | 22.37 (12.96; NR [NR]); 51.6% | NR | Life expectancy |
| Proffit et al. (2019) (10) | Questionnaire based study | USA | 2,029 | PWS patients | NR; NR | NR | Life expectancy |
| Whittington et al. (2015) (11) | Population-based cohort study | UK | 62 | PWS patients | NR; NR | 1998 to 2009 (11 years) | Mortality rate  Cause of death |
| **Humanistic burden** | | | | | | | |
| Adams et al. (2018) (12) | Observational cross-sectional study with Bayesian methodology | UK | 712 (PWS: 101) | PWS mothers  Other rare disease mothers | PWS mothers: 44.5 (8.5; NR [NR]); 100%  PWS child: 12.2 (8.1; NR [2-45]); 47.7% | NR | QoL scale  Caregiver/family burden |
| Allas et al. (2018) (13) | Multicenter, randomized, placebo-controlled trial | France  Italy  Spain | 47 | PWS patients | Treatment: 26.8 (6.7; NR [NR]); 35%  Placebo: 25.7 (5.6; NR [NR]); 67% | 14 days | Clinical scale |
| Amaro et al. (2022) (14) | Observational cross-sectional study | Brazil | 41 | PWS patients | 11.62 (7.9; NR [NR]); 41.5% | NR | QoL scale  Caregiver/family burden |
| Andrews et al. (2024) (15) | Observational cross-sectional study | USA | 99 (PWS; 17) | PWS children  Children with other neurogenetic condition  Age matched controls | PWS: 66.2 (9.4; NR [NR]) (months); 59%  Controls: 65.7 (11.0; NR [NR]) (months); 37% | NR | Clinical scale |
| Avrahamy et al. (2015) (16) | Questionnaire study | Israel | 53 | PWS patients | 23.6 (8.2; NR [NR]); 47% | NR | QoL scale  Clinical scale  Caregiver/family burden |
| Baietto et al. (2023) (17) | Prospective observational study | Italy | 71 (PWS: 34) | PWS parents  Community control parents | All parents: 45.56 (5.50; NR [32-59]); 88.7% (PWS: 82.4%)  All children: 10.56 (3.08; NR [6-17]); (PWS: 52.9%, Controls: 51.4%) | April 2020 to June 2020 (2 months) | QoL scale |
| Bakker et al. (2015) (18) | Multicenter randomized controlled trial and longitudinal study | Netherlands | 102 | PWS children | 8.1 (NR; NR [6.8-11.4]); 65.4% | April 2002 to June 2014 (12 years 2 months) | QoL scale  Caregiver/family burden |
| Bellicha et al. (2020) (19) | Observational cross-sectional study with additional interventional component | NR | 30 (PWS: 10) | PWS adults  Obese adults | PWS: 28.8 (24.2, 33.0) (median (IQR))  Obese: 30 (26.0, 36.0) (median (IQR)) | June 2016 to January 2018 (1 year 7 months) | QoL scale  Clinical scale |
| Bos-Roubos et al. (2022) (20) | Observational cross-sectional study | Netherlands | 98 | PWS family members | NR; 70.4% | September 2020 to July 2021 (10 months) | Caregiver/family burden |
| Bravo et al. (2016) (21) | Pilot double-blind, sham controlled, multicenter study | USA | 32 (PWS: 10) | PWS patients  Obese patients  Lean patients | PWS: 32.5 (11; NR [NR]); 50%  Obese: 46.9 (11; NR [NR]); NR  Lean: 27.8 (11; NR [NR]); NR | NR | Clinical scale |
| Bridges et al. (2018) (22) | Observational cross-sectional online survey | USA | 332 | PWS caregivers | Patients: 15.7 (NR; NR [4-54]); 82% | NR | QoL scale |
| Butler et al. (2016) (23) | Phase 3 randomized placebo-controlled trial | USA | 107 | PWS patients | 20 (6; NR [NR]); 48% | 26 weeks | Clinical scale |
| Cagalj et al. (2018) (24) | Qualitative interview study | Croatia | 5 | PWS mothers | 44 (NR; NR [28-62]) | 2013 | Caregiver/family burden |
| Chevreul et al. (2016) (25) | Retrospective cross-sectional study | France | 51 | PWS patients | 17.4 (12.2; NR [NR]); 55% | September 2012 to May 2013 (8 months) | QoL scale  Caregiver/family burden |
| Chiarotti et al. (2023) (26) | Observational cross-sectional study | Italy | 24 | PWS family caregivers | Caregivers: 44.3 (9.7; 43 [28-66]); 85.7%  Patients: 11.4 (8.0; 10 [1-29]); 54.2% | 2012 | QoL scale  Caregiver/family burden |
| Chiu et al. (2017) (27) | Observational cross-sectional study | Taiwan | 7 | PWS patients  PWS caregivers | Patients: 25.28 (5.10; NR [18-31]); 28.6% | NR | QoL scale |
| Choong et al. (2022) (28) | Observational cross-sectional study and registry analysis | Australia | 54 | PWS patients | 12.5 (5.4; NR [4.4-24.0]); 60% | May 2018 to March 2021 (2 years 10 months) | Clinical scale |
| Cotter et al. (2023) (29) | Qualitative interview, cross-sectional and longitudinal study | Australia  Canada  USA | Interview: 12  Cross-sectional: 416  Longitudinal: 130 | PWS patients  PWS caregivers | Caregivers: 49.4 (NR; NR [37-60]); 91.7%  Patients: 12.0 (3.36; 12.0 [7.0-19.0]); 55.4% | NR | Clinical scale |
| Coupaye et al. (2016) (30) | Observational cross-sectional study | France | 73 | PWS patients | 25 (NR; NR [16-58]); 51% | July 2007 to September 2015 (8 years 2 months) | Clinical scale |
| Coutant et al. (2023) (31) | Observational cross-sectional study | France | 275 (PWS: 11) | GH treated children | NR | NR | QoL scale  Treatment burden |
| Currie et al. (2024) (32) | Narrative inquiry | Canada | 4 | PWS mothers | NR | July 2021 to June 2022 (11 months) | Caregiver/family burden |
| Dobrescu et al. (2019) (33) | Observational cross-sectional study | Romania | 11 | PWS patients | Female: 25.37 (6.36; NR [NR])  Male: 10.37 (6.77; NR [NR]); 36.36% | NR | Clinical scale |
| Downs et al. (2022) (34) | Observational cross-sectional study | Australia | 52 | PWS patients | NR (NR; 12.1 [4.4-24.0]); 58% | May 2018 to March 2021 (2 years 10 months) | Clinical scale |
| Dykens et al. (2024) (35) | Multi-phase study | Australasia  Canada  Europe  USA | Pilot study: 112  Large-scale: 761 | PWS patients  PWS parents | All patients: 18.00 (10.81; NR [NR]); 52.2% | NR | Clinical scale |
| Dykens et al. (2024) (36) | Multi-phase study | USA | Pilot study: 133  Large-scale: 624  Follow-up: 119 | PWS patients  PWS parents | Pilot study: 18.86 (7.89; NR [5-43]); 57.9%  Lare-scale: 18.60 (10.65; NR [5-59]); 54.0%  Follow-up: 14.25 (7.75; NR [5-54]) 52.0% | NR | Clinical scale  Caregiver/family burden |
| Einfeld et al. (2014) (37) | Randomized, double-blind, controlled crossover trial | Australia | 30 | PWS patients | 17.8 (4.77; NR [12-29]); 33% | NR | Clinical scale |
| EUCTR2018-003062-13-ES (38) | Randomized, double-blind, placebo-controlled Phase 2b/3 study | Australia  Belgium  France  Italy  Netherlands  Spain  UK  USA | 158 | PWS patients | NR (NR; NR [2-64]); 55.7% | 25 March 2019 to 25 May 2020 (1 year 2 months) | Clinical scale |
| Faye et al. (2023) (39) | Retrospective database analysis | France | 403 | PWS children | NR (NR; 9.85 [NR]); 49.4% | 2005 to 2021 (16 years) | Treatment burden |
| Feighan et al. (2020) (40) | Observational cross-sectional study | Ireland | 61 | PWS caregivers | Patients: 16.3 (11.3; NR [0-52]); 58% | NR | Clinical scale  Caregiver/family burden  Treatment burden |
| Foerste et al. (2016) (41) | Observational cross-sectional study | Australia | 52 (PWS: 16) | PWS children and adolescents  T21 children and adolescents  LRO children and adolescents | PWS: 12.1 (4.1; NR [NR]); 56.3%  T21: 13.8 (2.6; NR [NR]); 64.7%  LRO: 11.7 (2.9; NR [NR]); 47.4% | NR | Clinical scale |
| Friedman et al. (2018) (42) | Observational interview study | USA | 1,473 (PWS: 5) | Various disability patients | NR | Recruitment: January 2015 to January 2017 (2 years) | QoL scale |
| Giordano et al. (2015) (43) | Prospective, observational, single-center study | Italy | 5 | PWS children | 5.8 (NR; NR [5-7]); 40% | January 2007 to June 2010 (3 years 5 months) | QoL scale |
| Gonzalez-Ruiz et al. (2024) (44) | Observational cross-sectional study | Argentina | 69 | PWS caregivers | Patients: 22.8 (10.2; NR [NR]); 64%  Caregivers: 49.9 (14.8; NR | NR | QoL scale  Caregiver/family burden |
| Grolleau et al. (2022) (45) | Monocentric descriptive study | France | 147 | PWS families  PWS patients | Patients: 8.1 (5.2; 7.1 [0-19]); 49.0% | October 2016 to June 2018 (1 year 8 months) | Clinical scale |
| Harisseh et al. (2020) (46) | Randomized, double-blind, placebo-controlled, Phase 2a trial | NR | 47 | NR | NR | NR | Clinical scale |
| Hedgeman et al. (2017) (5) | Nationwide cohort study | Denmark | 15,655 (PWS: 155) | PWS patients  Age and sex matched controls | PWS patients: 18 (17; NR [NR]); 54.2%  Controls: 18 (17; NR [NR]); 54.2% | Data searched from 1995 to December 2012 (17 years), births traced back to January 1977 (35 years) | Patient burden |
| Hollander et al. (2021) (47) | Randomized, double-blind, placebo-controlled trial | USA | 23 | PWS children | 8.87 (3.68; NR [NR]); 22% | NR | Clinical scale |
| Honea et al. (2023) (48) | Secondary analysis of interventional study | USA | 105 (PWS: 41) | PWS parents  Obese patient parents | Parents: 42.8 (7.5; NR [NR]); 88.5% | 24 weeks | Caregiver/family burden |
| Ihara et al. (2014) (49) | Observational cross-sectional study | Japan | 45 | PWS children and adolescents | 12.42 (3.90; NR [NR]); 35.6% | NR | QoL scale  Caregiver/family burden |
| Kayadjanian et al. (2018) (50) | Observational cross-sectional study | USA | 142 | PWS caregivers | NR | 10 May 2016 to 10 June 2016 (1 month) | QoL scale  Caregiver/family burden |
| Kayadjanian et al. (2021) (51) | Observational cross-sectional study | USA | 204 | PWS caregivers | NR | 10 May 2016 to December 2016 (7 months) | QoL scale  Clinical scale  Caregiver/family burden |
| Kendall et al. (2019) (52) | Case-control study | UK | 407,074 (PWS: NR) | Rare copy number variant disorder patients | NR | January 2017 to September 2018 (1 year 8 months) | QoL measure |
| Kowal et al. (2022) (53) | Qualitative sociological study | Poland | 20 | PWS caregivers | Caregivers: 41.95 (6.85; NR [30-56]); 85% | 01 July 2019 to 30 September 2019 (3 months) | Caregiver/family  burden  Patient burden |
| Krefft et al. (2021) (54) | Observational cross-sectional study | Poland | 40 (PWS: 20) | PWS patients  Typically developed controls | PWS: 11.5 (4.1; NR [NR]); 55%  Controls: 10.25 (2.9; NR [NR]); 45% | NR | QoL measure |
| Lavelle et al. (2020) (55) | Observational cross-sectional study | USA | 458 | PWS patients  PWS caregivers | NR | NR | QoL scale |
| Lavelle et al. (2021) (56) | Observational cross-sectional study | Canada  Europe  USA  “others” | 458 | PWS patients  PWS caregivers | Patients: 15.6 (NR; NR [4-54]); NR  Caregivers: 49.2 (NR; NR [22-83]); 83.4% | NR | QoL scale |
| Lo et al. (2015) (57) | Multicenter, randomized, controlled trial and longitudinal study | Netherlands | 42 | PWS children | 6.4 (4.9, 7.6) (median (IQR)) | April 2002 to 2010 (8 years) | Clinical scale |
| Lopez-Bastida et al. (2016) (58) | Observational cross-sectional study | Bulgaria  France  Germany  Hungary  Italy  Spain  Sweden  UK | 261 | PWS patients  PWS caregivers | Patients: 14 (NR; NR [6-22]); NR  Caregivers: 41 (NR; NR [34-45]); NR | September 2011 to April 2013 (1 year 7 months) | QoL scale  Caregiver/family burden |
| Maccarone et al. (2024) (59) | Observational cross-sectional study | Italy | 13 | PWS children | 11.61 (2.63; NR [8-15]); 76.9% | September 2020 to June 2021 (9 months) | QoL scale  Caregiver/family burden |
| Mackay et al. (2022) (60) | Observational cross-sectional and registry study | Australia | 52 | PWS patients  PWS caregivers | Patients: 11.8 (6.0, 16.3 (median (IQR))  55.8% | 2019 to 30 November 2020 (1 year) | QoL scale  Clinical scale  Caregiver/family burden |
| Mao et al. (2019) (61) | Observational cross-sectional study | China | 32 | PWS caregivers | 29.2 (21.2; NR [NR]) (months); 34.4% | NR | QoL scale |
| Mastey Ben-Yahuda et al. (2024) (62) | Observational and comparative study with follow-up | Israel | 58 | PWS adults | Specialized hostels: 32.8 (7.41; NR [19.60-38.45]); 51.5%)  Home/non-specialized hostels: 27.6 (7.41; NR [19.19-32.35]); 52.0% | 2013 to 2021 (8 years) | QoL scale |
| Matesevac et al. (2023) (63) | Observational cross-sectional study with qualitative interviews | USA | 1,073 (PWS: 459) | PWS patients  Typically developed controls | NR | NR | Clinical scale  Caregiver/family burden |
| McCandless et al. (2017) (64) | Randomized, placebo-controlled, double-blind Phase 3 trial | USA | 107 | PWS patients | Placebo: 20.9 (7.8; NR [NR]); 56%  1.8mg Beloranib: 19.2 (5.2; NR [NR]); 48%  2.4mg Beloranib: 19.5 (5.8; NR [NR]); 40% | 08 September 2014 to 11 December 2015 (1 year 3 months) | Clinical scale |
| McNulty et al. (2022) (65) | Retrospective patient review | NR | 69 | PWS patients | NR (NR; NR [5-22]); 72.5% | 2014 to 2022 (8 years) | Clinical scale |
| Meade et al. (2019) (66) | Observational cross-sectional study | Ireland | 19 | PWS patients  PWS parents | Patients: NR (NR; 7.9 [0-18]); 74% | NR | QoL scale  Caregiver/family burden |
| Meade et al. (2019) (67) | Observational cross-sectional study | Ireland | 19 | PWS patients  PWS parents | Patients: NR (NR; 7.9 [0-18]); 74% | NR | QoL scale  Caregiver/family burden  Patient burden |
| Meade et al. (2021) (68) | Observational cross-sectional study | Ireland | 19 | PWS patients  PWS parents | Patients: NR (NR; 7.9 [0-18]); 74% | NR | QoL scale  Caregiver/family burden  Patient burden |
| Miller et al. (2022) (69) | Randomized, double-blind, placebo-controlled, Phase 2, crossover trial | UK  USA | 19 | PWS patients | 22.1 (NR; NR [16-33]); 47% | 20 February 2018 to 12 June 2019 (1 year 4 months) | QoL scale  Clinical scale |
| Miller et al. (2023) (70) | Randomized, double-blind, placebo-controlled, parallel-group, Phase 3 trial | UK  USA | 124 | PWS patients | 13.5 (6.98; NR [NR]); 55.6% | May 2018 to January 2020 (1 year 8 months) | Clinical scale |
| Miller et al. (2024) (71) | Long-term open-label study | USA | 124 | PWS patients | 13.4 (6.98; NR [4-44]) | May 2018 to January 2020 (1 year 8 months) | Clinical scale |
| Montes et al. (2020) (72) | Observational longitudinal study | USA | 172 | PWS patients | NR; 55% | 07 September 2006 to 31 July 2014 (7 years 10 months) | QoL measure |
| NCT02629991 (2015) (73) | Randomized, placebo-controlled, interventional Phase 2 study with parallel assignment | USA | 23 | PWS children and adolescents | Treatment: 9.72 (3.50; NR [NR]); 18.2%  Placebo: 8.08 (3.82; NR [NR]); 25.0% | NR | Clinical scale |
| NCT02844933 (2016) (74) | Randomized, double-blind, placebo-controlled, interventional Phase 2 trial with parallel assignment | USA | 7 | PWS children | Treatment: 13.50 (NR; NR [10-15]); 50.0%  Placebo: 11.67 (NR; NR [8-15]); 33.3% | 09 May 2018 to 31 July 2019 (1 year 2 months) | Clinical scale |
| O’Neill et al. (2016) (75) | Observational cross-sectional survey | UK | 264 (PWS: 26) | PWS siblings  Other disabled patient siblings  Control siblings | PWS siblings: 30.65 (9.70; NR [NR]); 69.2%  Control siblings: 37.03 (12.34; NR [NR]); 74.2% | NR | QoL scale  Caregiver/family burden |
| Paepegaey et al. (2018) (76) | Retrospective reference center study | France | 95 | PWS patients | 24.7 (8.2; NR [16.1-58.7]); 53.7% | January 2007 to June 2017 (10 years 5 months) | Caregiver/family burden  Patient burden |
| Patel et al. (2021) (77) | Cross-sectional interview study | USA | 18 | PWS patients  PWS caregivers | Patients: 14 (NR; NR [6-36]); NR  Caregivers: 49 (NR; NR [30-61]); 100% | April 2020 to June 2020 (2 months) | Patient burden |
| Patel et al. (2022) (78) | Cross-sectional interview study | USA | 18 | PWS patients  PWS caregivers | Patients: NR  Caregivers: 49 (NR; NR [30-61]); 100% | 27 April 2020 to 01 June 2020 (1 month) | Caregiver/family burden  Patient burden |
| Pedemonti et al. (2023) (79) | Observational cross-sectional study | Argentina | 43 | PWS patients | Treatment: 18.4 (8.3; NR [NR]); 33%  Control: 19.1 (6.9; NR [NR]); 36% | NR | Clinical scale |
| Peleggi et al. (2021) (80) | Observational cross-sectional registry study | USA | 750 | PWS patients | NR; 51.3% | April 2015 to 17 December 2020 (5 years 8 months) | Patient burden |
| Ragusa et al. (2020) (81) | Observational, multicenter narrative study | Italy | 193 | PWS children  PWS adults  PWS caregivers | PWS children: 14 (3.09; NR [NR]); 29%  PWS adults: 29 (9.72; NR [NR]); 56%  PWS caregivers: 48 (9.04; NR [NR]); 72% | October 2018 to July 2019 (9 months) | Caregiver/family burden  Patient burden |
| Reilly et al. (2015) (82) | Observational cross-sectional study | UK  Ireland | 381 (PWS: 110) | PWS parents  Other neurogenetic syndrome parents | All patients: 11.2 (3.71; NR [NR]); 38% | 2011 | Caregiver/family burden |
| Rice et al. (2015) (83) | Observational longitudinal study | Australia | 248 (PWS: 51) | PWS patients  Other syndrome patients | NR | 1991 to 2008 (17 years) | Clinical scale |
| Rice et al. (2016) (84) | Observational longitudinal study | Australia | 524 (PWS: NR) | PWS patients  Autism patients | PWS: 8.14 (4.01; NR [NR]); 33%  Autism: 14.84 (6.12; NR [NR]); 16% | 1991 to 2008 (17 years) | Caregiver/family burden |
| Roof et al. (2023) (85) | Randomized, double-blind, placebo-controlled, Phase 3 trial with long-term follow-up | Australia Canada  USA | 130 | PWS patients | 3.2mg: 12.3 (3.12; NR [NR]); 61.5%)  9.6mg: 11.7 (3.45; NR [NR]); 47.5%  Placebo: 1.8 (3.52; NR [NR]); 57.5% | 06 December 2018 to 12 March 2020 (1 year 3 months) | Clinical scale |
| Royston et al. (2018) (86) | Observational cross-sectional study | NR | 111 (PWS: 26) | PWS patients  Other syndrome patients | 26.41 (10.38; NR [NR]); 33.3% | NR | Patient burden |
| Royston et al. (2020) (87) | Observational cross-sectional study | Canada  UK  USA | 111 (PWS: 26) | PWS patients  Other syndrome patients | 26.53 (10.36; NR [NR]); 33% | NR | Clinical scale |
| Rozensztrauch et al. (2022) (88) | Observational cross-sectional study | Poland | 46 | PWS children  PWS caregivers | Children: NR; 65.5%  Caregivers: 38.7 (7.8; NR [NR]); NR | NR | QoL scale  Caregiver/family burden |
| Rubin et al. (2019) (89) | Prospective quasi-randomized, controlled trial | USA | 111 (PWS: 45) | PWS children  Obese children | PWS: 10.9 (0.4; NR [NR]); 44.4%  Obese: 9.7 (1.3; NR [NR]); 47.0% | March 2011 to March 2014 (3 years) | QoL scale |
| Rubin et al. (2021) (90) | Prospective quasi-randomized, controlled trial | USA | 107 (PWS: 42) | PWS children  Obese children | PWS: 10.9 (0.4; NR [NR]); 44.4%  Obese: 9.8 (0.1; NR [NR]); 47.0% | March 2011 to April 2015 (4 years 1 month) | QoL scale |
| Schofield et al, (2021) (91) | Qualitative interview study | Australia | 14 (PWS: 8) | Medical Professionals  Teachers  PWS parents | PWS children: NR; 62.5% | NR | Caregiver/family burden  Patient burden |
| Shivers et al. (2016) (92) | Observational cross-sectional and longitudinal study | USA | 196 | PWS mothers | Children: 13.47 (9.41; NR [NR]); 50.5%  Mothers: 45.26 (9.99; NR [NR]); 100% | NR | QoL scale  Clinical scale  Caregiver/family burden |
| Shriki-Tal et al. (2017) (93) | Observational cross-sectional study | Israel | 53 | PWS patients | 23.6 (8.1; NR [NR]); 40% | NR | QoL scale  Clinical scale |
| Strong et al. (2024) (94) | Comparative study of long-term interventional and natural history studies | UK  USA | 343 | PWS patients (from C601/C602 and PATH studies) | C601/C602: 13.1 (6.17; NR [NR]); 57.9%  PATH: 17.9 (9.49; NR [NR]); 52.0 | May 2018 to January 2020 (1 year 8 months) | Clinical scale |
| Thomson et al. (2017) (95) | Quantitative survey and qualitative interview study | Australia | 19 (PWS: 6) | PWS parents  AS parents | PWS: NR; 33.3%  AS: NR; 61.5% | 2008 | QoL scale  Caregiver/family burden |
| Trueba-Timmermans et al. (2023) (96) | Open-label prospective study | Netherlands | 46 | PWS adolescents and adults | 18.9 (17.4, 20.5) (median (IQR)); 59% | 2011 (3 years) | Clinical scale |
| Tvrdik et al. (2015) (97) | Observational cross-sectional and interventional cohort study | USA | 57 | PWS parents | Parents: 46.3 (12.1; NR [28-82]); 54.4%  Children: 18.0 (13.6; NR [1-46]); NR | NR | QoL scale  Caregiver/family burden |
| Vitale et al. (2016) (98) | Qualitative interview study | USA | 20 | PWS families | Parents: NR (NR; NR [21-56]); NR  Children: NR (NR; NR [2-17]); 60% | NR | Caregiver/family burden |
| Wieting et al. (2021) (99) | Observational cross-sectional study | Germany | 89 | PWS patients | 21.3 (NR; NR [6-56]); 48.3% | 03 August 2020 to 25 August 2020 (22 days) | Caregiver/family burden |
| Wieting et al. (2021) (100) | Observational cross-sectional study | Germany | 37 (PWS: 24) | PWS patients  Obese controls | PWS: 27 (12.04; NR [NR]); 41.7%  Control: 32 (8.14; NR [NR]); 38.5% | NR | Clinical scale |
| Wieting et al. (2022) (101) | Observational cross-sectional study | Germany | 37 (PWS: 24) | PWS patients  Obese controls | PWS: 27 (12.04; NR [NR]); 41.7%  Control: 32 (8.14; NR [NR]); 38.5% | NR | Clinical scale |
| Wieting et al. (2023) (102) | Case-control study | Germany | 46 (PWS: 32) | PWS patients  Obese controls | PWS: 27.19 (11.45; NR [NR]); 40.6%  Control: 31.21 (8.65; NR [NR]); 35.7% | NR | Clinical scale |
| Wilson et al. (2016) (103) | Observational cross-sectional study | USA | 110 (PWS: 44) | PWS children  PWS parents  Obese children  Obese parents | PWS children: 11.0 (2.4; NR [NR]); 45.5%  PWS parents: NR; 88.6%  Obese children: 9.7 (1.1; NR [NR]); 47.0%  Obese parents: NR; 87.9% | NR | QoL scale |
| Wong et al. (2021) (104) | Observational cross-sectional study | Taiwan | 67 | PWS patients  PWS families | Children: 14.9 (8.3; NR [NR]); 49.3% | March 2018 to August 2019 (1 year 5 months) | QoL scale  Caregiver/family burden |
| Yamada et al. (2022) (105) | Observational cross-sectional study | Japan | 53 (PWS: 21) | PWS patients  Typically developed controls | PWS: NR (NR; 26.0 [15-50]); 42.9%  Control: NR (NR; 22.0 [15-48]); 37.5% | NR | Clinical scale |
| **Economic burden** | | | | | | | |
| Avram et al. (2018) (1) | Retrospective cohort study | NR | 2,029,004 (PWS: 39) | PWS pregnant mothers | NR | NR | Direct costs |
| Baker et al. (2023) (106) | Retrospective cross-sectional study | Australia | 108 | PWS patients  FXS patients  AS patients  Dup15q patients  PWS caregivers  FXS caregivers  AS caregivers  Dup15q caregivers | PWS patients: 10.32 (10.40; NR [NR]); 53.1%  FXS patients: 9.44 (9.24; NR [NR]); 37.1%  AS patients: 11.63 (9.75; NR [NR]); 40.7%  Dup15q patients: 9.84 (7.18; NR [NR]); 42.9% | November 2016 to March 2019 (2 years 4 months) | Direct costs  Indirect costs |
| Bar et al. (2017) (107) | Retrospective cohort study | France | 61 | PWS infants | NR; 54% | 01 January 2012 to 31 December 2013 (2 years) | Hospitalization  Drug/procedure use  Outpatient service use |
| Bridges et al. (2018) (22) | Observational cross-sectional study | NR | 332 | PWS caregivers | Patients: 15.7 (NR; NR [4-54]); 82% | NR | QALY/TTO |
| Butler et al. (2020) (108) | Retrospective claims data analysis | NR | 9,726 (PWS: 1,621) | PWS patients  Control patients | NR;NR | NR | Direct costs |
| Chevreul et al. (2016) (25) | Retrospective cross-sectional study | France | 51 | PWS patients | 17.4 (12.2; NR [NR]); 55% | September 2012 to May 2013 (enrolment) (8 months) | Direct costs  Indirect costs |
| Chung et al. (2019) (109) | Retrospective cohort study | USA | 26,060 (PWS: 160) | Scoliosis patients | NR;NR | 2001 to 2012 (11 years) | Hospitalization |
| Clerc et al. (2021) (110) | Multicenter retrospective descriptive study | France | 39 | PWS patients | 25.6 (23.1, 31.7) (median (IQR)); 56.4% | July 2020 to April 2021 (9 months) | Hospitalization  Drug/procedure use |
| Elliot et al. (2015) (111) | Retrospective case-control study | USA | 320 | PWS patients | 13.1 (2.9; NR [NR]); 42.3% | March 2002 to May 2012 (10 years 2 months) | Drug/procedure use |
| Feighan et al. (2020) (40) | Cross-sectional study using national survey data | Ireland | 61 | PWS caregivers | Patients: 16.3 (11.3; NR [0-52]); 57.4% | NR | Indirect costs  Outpatient service use |
| Gul et al. (2022) (112) | Retrospective analysis | Turkey | 14 (PWS: 1) | Recurrent infection patients | NR | January 2006 to August 2020 (14 years 7 months) | Hospitalization  Drug/procedure use |
| Hughes et al. (2024) (113) | Retrospective observational study using archival data | Denmark  Germany  Ireland  Switzerland  UK  USA | 193 | PWS patients | 37.29 (13.30; NR [14-87]); NR | NR | Outpatient service use |
| Kayadjanian et al. (2018) (50) | Observational cross-sectional study | USA | 142 | PWS caregivers | NR | 10 May 2016 to 10 June 2016 (1 month) | Indirect costs  Living situation |
| Kayadjanian et al. (2021) (51) | Observational cross-sectional study | USA | 204 | PWS caregivers | NR | 10 May 2016 to December 2016 (7 months) | Living situation |
| Laurier et al. (2015) (114) | Retrospective cohort study | France | 154 | PWS patients | 28.7 (7.7; NR [NR]); 55.8% | 4 years | Living situation |
| Lavelle et al. (2021) (56) | Observational cross-sectional study | Canada  Europe  USA  “others” | 458 | PWS patients  PWS caregivers | Patients: 15.6 (NR; NR [4-54]); NR  Caregivers: 49.2 (NR; NR [22-83]); 83.4% | NR | QALY/TTO |
| Lopez-Bastida et al. (2016) (58) | Observational cross-sectional study | Bulgaria  France  Germany  Hungary  Italy  Spain  Sweden  UK | 261 | PWS patients  PWS caregivers | Patients: 14 (NR; NR [6-22]); NR  Caregivers: 41 (NR; NR [34-45]); NR | September 2011 to April 2013 (1 year 7 months) | Direct costs |
| Luccarrelli (2022) (115) | Cross-sectional database analysis | USA | 540 | PWS patients | 22 (6.3, 37.8) (median (IQR)); 48.1% | 2019 | Direct costs  Hospitalization  Drug/procedure use |
| McQuivey et al. (2021) (116) | Retrospective comparative study | USA | 16,698 (PWS: 100) | Scoliosis patients | 11.6 (NR; NR [NR]); 52% | 2000 to 2012 (12 years) | Direct costs |
| McQuivey et al. (2022) (117)  D | Retrospective cohort study | NR | 334 | PWS patients | 10.33 (4.5; NR [NR]); 47% | 2001 to 2012 (11 years) | Hospitalization  Drug/procedure use |
| Meade et al. (2019) (118) | Observational cohort study | NR | 19 | PWS children | NR (NR; 7.9 [0-18]); 74% | NR | Drug/procedure use |
| Peleggi et al. (2021) (80) | Registry analysis | Canada  USA  “others” | 750 | PWS patients | NR; 51.3% | April 2015 to December 2020 (5 years 8 months) | Hospitalization  Drug/procedure use |
| Pellikaan et al. (2021) (119) | Retrospective cohort study and literature review | Netherlands | 122 | PWS adults | 29 (21, 39) (median (IQR)); 52.5% | January 2015 to December 2020 (5 years 11 months) | Drug/procedure use  Living situation |
| Pemmasani et al. (2021) (120) | Retrospective database analysis | NR | 480 | PWS patients | 27 (19; NR [NR]); 49.6% | 2014 | Hospitalization |
| Potluri et al. (2015) (121) | ACER review | UK | NA | PWS patients | NR | NR | Economic evaluation |
| Prapasrat et al. (2021) (122) | Comparative diagnostic study | Thailand | 40 | PWS patients | NR | NR | Diagnostic costs |
| Ragusa et al. (2020) (81) | Multicenter narrative study | Italy | 193 | PWS patients  PWS caregivers | Child patients: 14 (3.09; NR [7-18]); 29%  Adult patients: 29 (9.72; NR [19-48]); 56%  Caregivers: 48 (9.04; NR [20-61]); 72% | October 2018 to July 2019 (9 months) | Indirect costs |
| Sequeira et al. (2021) (123) | Meta-analysis | Bulgaria  France  Germany  Hungary  Italy  Spain  Sweden  UK | NR | Rare disease patients | NR | January 2010 to February 2017 (7 years 1 month) | Direct costs |
| Shepherd et al. (2019) (124) | National audit | UK | 3,757 (PWS: 214) | GH treated patients | PWS: NR (NR; 2.2 [0-15]); 52.3% | January 2013 to December 2016 (3 years 11 months) | Drug/procedure use |
| Shofstall et al. (2016) (125) | Retrospective case-control and longitudinal study using claims data | USA | 2,030 | PWS patients | Commercially insured/Medicare: 15.6 (16.8; 10 [0-92]); 50.1%  Medicaid: 19.4 (16.6; 17 [0-90]);47.2% | 2009 to 2014 (5 years) | Direct costs  Hospitalization  Drug/procedure use  Outpatient service use |
| Van Bosse et al. (2020) (126) | Database analysis | NR | 9,414,850 (weighted) (PWS: 65) | Hip/knee replacement patients | PWS: NR; 47.4% | 2004 to 2014 (10 years) | Direct costs |
| Yang et al. (2019) (127) | Registry analysis | China | 134 | PWS mothers  PWS infants | Mothers: 30.5 (NR; NR [NR]); 100%  Infants: NR; NR | NR | Drug/procedure use |
| Yang et al. (2020) (128) | Registry analysis | China | 134 | PWS mothers  PWS infants | Mothers: 30.5 (5.5; NR [18-46]); 100%  Infants: NR; 45.5% | October 1997 to January 2009 (mothers birth date) (11 years 3 months) | Hospitalization  Drug/procedure use |

Abbreviations: ACER, average cost-effectiveness ratio; AS, Angelman syndrome; Dup15q, Chromosome 15q duplication syndrome; FXS, Fragile X syndrome; GH, growth hormone; IQR, inter-quartile range; LRO, Lifestyle-related obesity; NA, not applicable; NR, not reported; PWS, Prader-Willi syndrome; QoL, quality of life; SD, standard deviation; T21, Trisomy 21;

Clinical scale includes Developmental Behaviour Checklist (DBC), Food Safe Zone (FSZ), Dykens Hyperphagia Questionnaire, Hyperphagia Questionnaire for Clinical Trials (HQ-CT), Prader-Willi syndrome Anxiousness and Distress (PADQ) and Prader-Willi Syndrome Profile (PWSP).

Supplemental table 6 summary of QoL scales reported in publications from the humanistic burden search

| **QoL Scales** | **Number of publications** | **References** |
| --- | --- | --- |
| Barthel index | 2 | (26, 58) |
| CSSRS | 1 | (69) |
| DSM-5 | 1 | (17) |
| DUX25/DUXPW | 1 | (18) |
| EQ-5D | 4 | (25, 26, 56, 58) |
| FSCI | 1 | (95) |
| HADS | 3 | (12, 44, 75) |
| PAS5 | 1 | (12) |
| PedsQL | 9 | (43, 44, 66-68, 88-90, 103) |
| PedsQL FIM | 5 | (44, 66-68, 88) |
| Personal outcome measures | 1 | (42) |
| PGS | 1 | (12) |
| PSI | 3 | (27, 92, 104) |
| PSS-14 | 1 | (97) |
| PWSBQ | 2 | (16, 62) |
| QALY | 3 | (22, 55, 56) |
| QOLISSY | 1 | (31) |
| QRSF | 1 | (12) |
| SF-12 | 2 | (19, 60) |
| SF-36 | 3 | (27, 62, 93) |
| SWLS | 1 | (92) |
| Time trade-off | 2 | (22, 56) |
| W-ADL | 1 | (62) |
| WHOQOL-BREF | 5 | (14, 27, 44, 49, 61) |
| Zarit burden interview | 7 | (25, 26, 44, 50, 51, 58, 59) |
| None | 57 |  |

Abbreviations: CSSRS, Columba Suicide Severity Rating Scale; DSM-5, Diagnostic and Statistical Manual of Mental Disorders, fifth edition; DUX25/PW, Dutch Children AZL/TNO Questionnaire Quality Of Life short form/PWS; EQ-5D, EuroQol-5 Dimension; FSCI, Family Stress and Coping Interview; HADS, Hospital Anxiety and Depression Scale; PAS5, Positive Affect Scale-5; PedsQL,(FIM) Pediatric Quality of Life Inventory (Family Impact Module); PGS, Positive Gain Scale; PSI, Parenting Stress Index; PSS-14, Perceived Stress Scale 14; PWSBQ, Prader-Willi syndrome Behavioral Questionnaire; QALY, quality-adjusted life year; QOLISSY, Quality of Life in Short Stature Youth; QRSF Questionnaire of Resources and Stress; SF-12/36, Short Form 12/36; SWLS, Satisfaction with Life Scale; W-ADL, Waisman Activities of Daily Living Scale; WHOQOL, World Health Organization Quality of Life Brief Version

References

1. Avram CM, Allen AJ, Shaffer BL, Caughey AB. The impact of Prader Willi Syndrome on perinatal outcomes. American Journal of Obstetrics and Gynecology. 2018;218(1 Supplement 1):S288-S9.

2. Bourke J, Wong K, Leonard H. Validation of intellectual disability coding through hospital morbidity records using an intellectual disability population-based database in Western Australia. BMJ Open. 2018;8(1) (no pagination).

3. Butler MG, Manzardo AM, Heinemann J, Loker C, Loker J. Causes of death in Prader-Willi syndrome: Prader-Willi Syndrome Association (USA) 40-year mortality survey. Genet Med. 2017;19(6):635-42.

4. Butler MG, Kimonis V, Dykens E, Gold JA, Miller J, Tamura R, et al. Prader-Willi syndrome and early-onset morbid obesity NIH rare disease consortium: A review of natural history study. Am J Med Genet A. 2018;176(2):368-75.

5. Hedgeman E, Ulrichsen SP, Carter S, Kreher NC, Malobisky KP, Braun MM, et al. Long-term health outcomes in patients with Prader-Willi Syndrome: a nationwide cohort study in Denmark. Int J Obes (Lond). 2017;41(10):1531-8.

6. Manzardo AM, Loker, J., Heinemann, J., Loker, C., Butler, M. G. Survival trends from the Prader-Willi Syndrome Association (USA) 40-year mortality survey. Genet Med. 2018;20(1):24-30.

7. McCandless SE, Suh M, Yin D, Yeh M, Czado S, Aghsaei S, et al. U.S. Prevalence & Mortality of Prader-Willi Syndrome: A Population-Based Study of Medical Claims. Journal of the Endocrine Society. 2020;4(Supplement 1):A504-A5.

8. Pacoricona Alfaro DL, Lemoine P, Ehlinger V, Molinas C, Diene G, Valette M, et al. Causes of death in Prader-Willi syndrome: lessons from 11 years' experience of a national reference center. Orphanet Journal Of Rare Diseases. 2019;14(1):238.

9. Proffitt J, Osann K, McManus B, Kimonis VE, Heinemann J, Butler MG, et al. Contributing factors of mortality in Prader-Willi syndrome. Am J Med Genet A. 2019;179(2):196-205.

10. Proffit JN, Osann K., MacManus B., Butler M. G., Kimonis V. E., Heinemann J., Stevenson D., Gold J. A. A lower BMI and growth hormone use results in decreased mortality in Prader-Willi syndrome. European Journal of Human Genetics. 2019;27(Supplement 2):1520-1.

11. Whittington JE, Holland AJ, Webb T. Ageing in people with Prader-Willi syndrome: mortality in the UK population cohort and morbidity in an older sample of adults. Psychol Med. 2015;45(3):615-21.

12. Adams D, Hastings R. P., Alston-Knox C., Cianfaglione R., Eden K., Felce D., Griffith G., Moss J., Stinton C., Oliver C. Using Bayesian methodology to explore the profile of mental health and well-being in 646 mothers of children with 13 rare genetic syndromes in relation to mothers of children with autism. Orphanet Journal Of Rare Diseases. 2018;13(1):185.

13. Allas S, Caixàs A., Poitou C., Coupaye M., Thuilleaux D., Lorenzini F., Diene G., Crinò A., Illouz F., Grugni G., et al. AZP-531, an unacylated ghrelin analog, improves food-related behavior in patients with Prader-Willi syndrome: a randomized placebo-controlled trial. PLoS ONE. 2018;13(1):e0190849.

14. Amaro AS, Rubin D. A., Teixeira Mctv Ferreira A. J. Jr, Rodrigues G. M., Carreiro L. R. R. Health Problems in Individuals With PWS Are Associated With Lower Quality of Life for Their Parents: A Snapshot in the Brazilian Population. Front. 2022;10:746311.

15. Andrews SM, Panjwani A. A., Potter S. N., Hamrick L. R., Wheeler A. C., Kelleher B. L. Specificity of Early Childhood Hyperphagia Profiles in Neurogenetic Conditions. Am J Intellect Dev Disabil. 2024;129(3):175-90.

16. Avrahamy H, Pollak Y., Shriki-Tal L., Genstil L., Hirsch H. J., Gross-Tsur V., Benarroch F. A disease specific questionnaire for assessing behavior in individuals with Prader-Willi syndrome. Compr Psychiatry. 2015;58:189-97.

17. Baietto C, Bechis D., Caldarera A. M., Marcotulli D., Natali Sora M. G., Vitiello B. Children with Prader-Willi Syndrome and COVID-19: a longitudinal study of the effect of social re-opening after the lockdown. Minerva Pediatr (Torino). 2023;26:26.

18. Bakker NE, Siemensma E. P., van Rijn M., Festen D. A., Hokken-Koelega A. C. Beneficial Effect of Growth Hormone Treatment on Health-Related Quality of Life in Children with Prader-Willi Syndrome: A Randomized Controlled Trial and Longitudinal Study. Hormone research in paediatrics. 2015;84(4):231-9.

19. Bellicha A, Coupaye M., Hocquaux L., Speter F., Oppert J., M. Poitou C. Increasing physical activity in adult women with Prader-Willi syndrome: A transferability study. J Appl Res Intellect Disabil. 2020;33(2):258-67.

20. Bos-Roubos A, Wingbermuhle E., Biert A., de Graaff L., Egger J. Family Matters: Trauma and Quality of Life in Family Members of Individuals With Prader-Willi Syndrome. Front Psychiatr. 2022;13:897138.

21. Bravo GL, Poje A. B., Perissinotti I., Marcondes B. F., Villamar M. F., Manzardo A. M., Luque L., LePage J. F., Stafford D., Fregni F., Butler M. G. Transcranial direct current stimulation reduces food-craving and measures of hyperphagia behavior in participants with Prader-Willi syndrome. Am J Med Genet B Neuropsychiatr Genet. 2016;171B(2):266-75.

22. Bridges JF, Lavelle T., Tsai J., Kayadjanian N., Strong T. Assessing the potential impact of treating hyperphagia among people with prader-willi syndrome using disease-specific qalys. Value in Health. 2018;21(Supplement 1):S256.

23. Butler MG, McCandless S, Roof E, Dykens EM, Fu C, Stafford DEJ, et al. Weight loss and improvement in hyperphagia-related behavior: Results from bestpws, a phase 3, randomized, placebo-controlled, clinical trial of beloranib, a methionine aminopeptidase 2 (MetAP2) inhibitor, in patients with prader-willi syndrome. Endocrine Reviews Conference: 98th Annual Meeting and Expo of the Endocrine Society, ENDO. 2016;37(2 Supplement 1).

24. Cagalj D, Buljevac M., Leutar Z. Being a mother of a child with Prader-Willi syndrome: Experiences of accessing and using formal support in Croatia. Scandinavian Journal of Disability Research. 2018;20(1):228-37.

25. Chevreul K, Berg Brigham K., Clement M. C., Poitou C., Tauber M. Economic burden and health-related quality of life associated with Prader-Willi syndrome in France. Journal of Intellectual Disability Research. 2016;60(9):879-90.

26. Chiarotti F, Kodra Y., De Santis M., Bellenghi M., Taruscio D., Care A., Petrini M. Gender and burden differences in family caregivers of patients affected by ten rare diseases. Ann Ist Super Sanita. 2023;59(2):122-31.

27. Chiu VJ, Tsai L. P., Wei J. T., Tzeng I. S., Wu H. C. Motor performance in Prader-Willi syndrome patients and its potential influence on caregiver's quality of life. Peerj. 2017;5:e4097.

28. Choong CS, Nixon G. M., Blackmore A. M., Chen W., Jacoby P., Leonard H., Lafferty A. R., Ambler G., Kapur N., Bergman P. B., Schofield C., Seton C., Tai A., Tham E., Vora K., Crock P., Verge C., Musthaffa Y., Blecher G., Wilson A., Downs J. Daytime sleepiness and emotional and behavioral disturbances in Prader-Willi syndrome. European Journal of Pediatrics. 2022;181(6):2491-500.

29. Cotter SP, Schwartz L., Strong T. V., Bender R. H., Fehnel S. E. The Prader-Willi Syndrome Anxiousness and Distress Behaviors Questionnaire: Development and Psychometric Validation. Value in Health. 2023;26(2):243-50.

30. Coupaye M, Tauber M., Cuisset L., Laurier V., Bieth E., Lacorte J. M., Oppert J. M., Clement K., Poitou C. Effect of Genotype and Previous GH Treatment on Adiposity in Adults With Prader-Willi Syndrome. J Clin Endocrinol Metab. 2016;101(12):4895-903.

31. Coutant R, Tauber M., Demaret B., Henocque R., Brault Y., Montestruc F., Chassany O., Polak M. Treatment burden, adherence, and quality of life in children with daily GH treatment in France. Endocrine Connections. 2023;12(4) (no pagination).

32. Currie G, Estefan A., Caine V. Mothering a Child With Complexity and Rarity: A Narrative Inquiry Exploring Prader-Willi Syndrome. Qual Health Res. 2024:10497323231225412.

33. Dobrescu A, Chirita-Emandi A., Andreescu N., Farcas S., Puiu M. Hyperphagia questionnaire to evaluate the Prader Willi patients behavior related to food. European Journal of Human Genetics. 2019;26(Supplement 1):865-6.

34. Downs J, Blackmore A. M., Chen W., Nixon G. M., Choong C. S. Strengths and challenging behaviors in children and adolescents with Prader-Willi syndrome: Two sides to the coin. Am J Med Genet A. 2022;188(5):1488-96.

35. Dykens EM, Roof E., Hunt-Hawkins H. The Prader-Willi syndrome Profile: validation of a new measure of behavioral and emotional problems in Prader-Willi syndrome. Orphanet Journal Of Rare Diseases. 2024;19(1):83.

36. Dykens EM, Roof E., Hunt-Hawkins H. Validation of the Food Safe Zone Questionnaire for Families of Individuals with Prader-Willi syndrome. medRxiv. 2024;28.

37. Einfeld SL, Smith E., McGregor I. S., Steinbeck K., Taffe J., Rice L. J., Horstead S. K., Rogers N., Hodge M. A., Guastella A. J. A double-blind randomized controlled trial of oxytocin nasal spray in Prader Willi syndrome. American Journal of Medical Genetics, Part A. 2014;164(9):2232-9.

38. EUCTR. A Clinical Study in patients with Prader-Willi-Syndrome (PWS) to test if a study drug named livoletide can reduce food related behaviour and be safe and well tolerated [Trial registry record]. 2019 [Available from: <https://trialsearch.who.int/Trial2.aspx?TrialID=EUCTR2018-003062-13-ES>]. Last accessed: 31 January 2025.

39. Faye S, Molinas, C., Brochado, C., Valette, M., Desprez, C., Diene, G., Arnaud, C., Tauber, M. The evolution of diagnosis and care over time in children with Prader-Willi syndrome, born between 2005 and 2021, included in the French database. European Societ for Paediatric Endocrinology meeting 2023. 2023.

40. Feighan SM, Hughes M., Maunder K., Roche E., Gallagher L. A profile of mental health and behaviour in Prader-Willi syndrome. Journal of Intellectual Disability Research. 2020;64(2):158-69.

41. Foerste T, Sabin M., Reid S., Reddihough D. Understanding the causes of obesity in children with trisomy 21: hyperphagia vs physical inactivity. Journal of Intellectual Disability Research. 2016;60(9):856-64.

42. Friedman C. The personal outcome measures<sup></sup>. Disability and Health Journal. 2018;11(3):351-8.

43. Giordano L, Toma S., Palonta F., Teggi R., Zucconi M., Di Candia S., Bussi M. Obstructive sleep apnea in Prader-Willi syndrome: Risks and advantages of adenotonsillectomy. Pediatria Medica e Chirurgica. 2015;37(2):8-11.

44. Gonzalez Ruiz Y, Gerk A., Stegmann J. Mental health impact on primary and secondary Prader-Willi syndrome caregivers. Child Care Health Dev. 2024;50(1):e13162.

45. Grolleau S, Delagrange M., Souquiere M., Molinas C., Diene G., Valette M., Tauber M. Impact of Deprivation on Obesity in Children with PWS. J. 2022;11(8) (no pagination).

46. Harisseh R, Delale T, Yeh M, Allas S. Livoletide (AZP-531), an Unacylated Ghrelin Analogue, Improves Hyperphagia and Food-Related Behaviors Both in Obese and Non-Obese People with Prader-Willi Syndrome. Journal of the Endocrine Society. 2020;4(Supplement 1):A510-A1.

47. Hollander E, Levine K. G., Ferretti C. J., Freeman K., Doernberg E., Desilva N., Taylor B. P. Intranasal oxytocin versus placebo for hyperphagia and repetitive behaviors in children with Prader-Willi Syndrome: A randomized controlled pilot trial. Journal of Psychiatric Research. 2021;137:643-51.

48. Honea KE, Wilson K. S., Fisher K. L., Rubin D. A. Parental and familial factors related to participation in a home-based physical activity intervention in children with obesity or Prader-Willi syndrome. Obes Pillars. 2023;8:100084.

49. Ihara H, Ogata H., Sayama M., Kato A., Gito M., Murakami N., Kido Y., Nagai T. QOL in caregivers of Japanese patients with Prader-Willi syndrome with reference to age and genotype. American Journal of Medical Genetics, Part A. 2014;164(9):2226-31.

50. Kayadjanian N, Schwartz L., Farrar E., Comtois K. A., Strong T. V. High levels of caregiver burden in Prader-Willi syndrome. PLoS ONE. 2018;13(3):e0194655.

51. Kayadjanian N, Vrana-Diaz C., Bohonowych J., Strong T. V., Morin J., Potvin D., Schwartz L. Characteristics and relationship between hyperphagia, anxiety, behavioral challenges and caregiver burden in Prader-Willi syndrome. PLoS ONE. 2021;16(3):e0248739.

52. Kendall KM, Rees E., Bracher-Smith M., Legge S., Riglin L., Zammit S., O'Donovan M. C., Owen M. J., Jones I., Kirov G., Walters J. T. R. Association of Rare Copy Number Variants With Risk of Depression. JAMA Psychiatry. 2019;76(8):818-25.

53. Kowal K, Skrzypek M., Kocki J. Experiencing illness as a crisis by the caregivers of individuals with Prader-Willi Syndrome. PLoS ONE. 2022;17(9):e0273295.

54. Krefft M, Frydecka D., Zalsman G., Krzystek-Korpacka M., Smigiel R., Gebura K., Bogunia-Kubik K., Misiak B. A pro-inflammatory phenotype is associated with behavioural traits in children with Prader-Willi syndrome. Eur Child Adolesc Psychiatry. 2021;30(6):899-908.

55. Lavelle TA, Crossnohere N. L., Bridges J. F. P. Eliciting quality adjusted life years using the time trade off method for Prader-Willi syndrome. Patient. 2020;13(1):140.

56. Lavelle TA, Crossnohere N. L., Bridges J. F. P. Quantifying the Burden of Hyperphagia in Prader-Willi Syndrome Using Quality-Adjusted Life-years. Clin Ther. 2021;43(7):1164-78.e4.

57. Lo ST, Siemensma E. P., Festen D. A., Collin P. J., Hokken-Koelega A. C. Behavior in children with Prader-Willi syndrome before and during growth hormone treatment: a randomized controlled trial and 8-year longitudinal study. Eur Child Adolesc Psychiatry. 2015;24(9):1091-101.

58. Lopez-Bastida J, Linertova R., Oliva-Moreno J., Posada-de-la-Paz M., Serrano-Aguilar P., Kanavos P., Taruscio D., Schieppati A., Iskrov G., Baji P., Delgado C., von der Schulenburg J. M. G., Persson U., Chevreul K., Fattore G. Social/economic costs and health-related quality of life in patients with Prader-Willi syndrome in Europe. European Journal of Health Economics. 2016;17(Supplement 1):99-108.

59. Maccarone MC, Avenia M., Masiero S. Postural-motor development, spinal range of movement and caregiver burden in Prader-Willi syndrome-associated scoliosis: an observational study. European J. 2024;34(2):22.

60. Mackay J, Nixon G. M., Lafferty A. R., Ambler G., Kapur N., Bergman P. B., Schofield C., Seton C., Tai A., Tham E., Vora K., Crock P., Verge C., Musthaffa Y., Blecher G., Caudri D., Leonard H., Jacoby P., Wilson A., Choong C. S., Downs J. Associations Between Hyperphagia, Symptoms of Sleep Breathing Disorder, Behaviour Difficulties and Caregiver Well-Being in Prader-Willi Syndrome: A Preliminary Study. J Autism Dev Disord. 2022;52(9):3877-89.

61. Mao SJ, Shen J., Xu F., Zou C. C. Quality of life in caregivers of young children with Prader-Willi syndrome. World J Pediatr. 2019;15(5):506-10.

62. Mastey Ben-Yehuda H, Gross-Tsur V., Hirsch H. J., Genstil L., Derei D., Forer D., Benarroch F. Quality of Life for Adults with Prader-Willi Syndrome in Residential Group Homes. J. 2024;13(11):04.

63. Matesevac L, Vrana-Diaz C. J., Bohonowych J. E., Schwartz L., Strong T. V. Analysis of Hyperphagia Questionnaire for Clinical Trials (HQ-CT) scores in typically developing individuals and those with Prader-Willi syndrome. Sci. 2023;13(1):20573.

64. McCandless SE, Yanovski JA, Miller J, Fu C, Bird LM, Salehi P, et al. Effects of MetAP2 inhibition on hyperphagia and body weight in Prader-Willi syndrome: A randomized, double-blind, placebo-controlled trial. Diabetes Obes Metab. 2017;19(12):1751-61.

65. McNulty BS, P. Kim, S. Kim, L. Howard, W. J. Aberrant Behavior Checklist Scores in Youth with Prader-Willi Syndrome. Pediatric Academic Societies Meeting 2023. 2023.

66. Meade C, Martin R., Crowe C., Lyons J., McCrann A., Roche E. The impact of caring for a child with Prader Willi syndrome. Archives of Disease in Childhood. 2019;104(Supplement 2):A227.

67. Meade C, Martin R., Lyons J., McCrann A., Roche E. Quality of life and the impact of caring for a child with Prader Willi Syndrome. Archives of Disease in Childhood. 2019;104(Supplement 3):A88-A9.

68. Meade C, Martin R., McCrann A., Lyons J., Meehan J., Hoey H., Roche E. Prader-Willi Syndrome in children: Quality of life and caregiver burden. Acta Paediatr. 2021;110(5):1665-70.

69. Miller JL, Lacroix A., Bird L. M., Shoemaker A. H., Haqq A., Deal C. L., Clark K. A., Ames M. H., Suico J. G., de la Peña A., et al. The Efficacy, Safety, and Pharmacology of a Ghrelin O-Acyltransferase Inhibitor for the Treatment of Prader-Willi Syndrome. Journal of clinical endocrinology and metabolism. 2022;107(6):e2373‐e80.

70. Miller JL, Gevers E., Bridges N., Yanovski J. A., Salehi P., Obrynba K. S., Felner E. I., Bird L. M., Shoemaker A. H., Angulo M., Butler M. G., Stevenson D., Abuzzahab J., Barrett T., Lah M., Littlejohn E., Mathew V., Cowen N. M., Bhatnagar A. Diazoxide Choline Extended-Release Tablet in People With Prader-Willi Syndrome: A Double-Blind, Placebo-Controlled Trial. J Clin Endocrinol Metab. 2023;108(7):1676-85.

71. Miller JL, Gevers E., Bridges N., Yanovski J. A., Salehi P., Obrynba K. S., Felner E. I., Bird L. M., Shoemaker A. H., Angulo M., Butler M. G., Stevenson D., Goldstone A. P., Wilding J., Lah M., Shaikh M. G., Littlejohn E., Abuzzahab M. J., Fleischman A., Hirano P., Yen K., Cowen N. M., Bhatnagar A. Diazoxide choline extended-release tablet in people with Prader-Willi syndrome: results from long-term open-label study. Obesity. 2024;32(2):252-61.

72. Montes AS, Osann K. E., Gold J. A., Tamura R. N., Driscoll D. J., Butler M. G., Kimonis V. E. Genetic Subtype-Phenotype Analysis of Growth Hormone Treatment on Psychiatric Behavior in Prader-Willi Syndrome. Genes (Basel). 2020;11(11):23.

73. ClinicalTrials.gov. Oxytocin vs. Placebo for the Treatment Hyperphagia in Children and Adolescents With Prader-Willi Syndrome NCT02629991 [Trial registry record]. 2015 [Available from: <https://www.cochranelibrary.com/central/doi/10.1002/central/CN-01554390/full>]. Last accessed: 31 January 2025.

74. ClinicalTrials.gov. Cannabidiol Oral Solution for the Treatment of Patients With Prader-Willi Syndrome NCT02844933 [Trial registry record]. 2016 [Available from: <https://www.cochranelibrary.com/central/doi/10.1002/central/CN-01507046/full>]. Last accessed: 31 January 2025.

75. O'Neill LP, Murray L. E. Anxiety and depression symptomatology in adult siblings of individuals with different developmental disability diagnoses. Res Dev Disabil. 2016;51-52:116-25.

76. Paepegaey AC, Coupaye M., Jaziri A., Menesguen F., Dubern B., Polak M., Oppert J. M., Tauber M., Pinto G., Poitou C. Impact of transitional care on endocrine and anthropometric parameters in Prader-Willi syndrome. Endocrine Connections. 2018;7(5):663-72.

77. Patel V, Davis K., Merikle E., McClure E., Patroneva A. PRO60 The Epworth Sleepiness Scale for Children and Adolescents Is a Fit-for-Purpose Measure of Daytime Sleepiness in Prader-Willi Syndrome: A Qualitative Interview Study. Value in Health. 2021;24(Supplement 1):S208.

78. Patel VP, Patroneva A., Glaze D. G., Davis K., Merikle E., Revana A. Establishing the content validity of the Epworth Sleepiness Scale for Children and Adolescents in Prader-Willi syndrome. J Clin Sleep Med. 2022;18(2):485-96.

79. Pedemonti B, Ceccomancini R., D'Acunti A., Stegmann J. Effectiveness of a transdisciplinary approach on hyperphagia management among patients with Prader Willi syndrome. Endocrinol Diabetes Nutr (Engl Ed). 2023;70(5):347-51.

80. Peleggi A, Bohonowych J., Strong T. V., Schwartz L., Kim S. J. Suicidality in individuals with Prader-Willi syndrome: a review of registry survey data. BMC Psychiatry. 2021;21(1):438.

81. Ragusa L, Crino A., Grugni G., Reale L., Fiorencis A., Licenziati M. R., Faienza M. F., Wasniewska M., Delvecchio M., Franzese A., Rutigliano I., Fusilli P., Corica D., Campana G., Greco D., Chiarito M., Sacco M., Toscano S., Marini M. G. Caring and living with Prader-Willi syndrome in Italy: integrating children, adults and parents' experiences through a multicentre narrative medicine research. BMJ Open. 2020;10(8):e036502.

82. Reilly C, Murtagh L., Senior J. The Impact on the Family of Four Neurogenetic Syndromes: A Comparative Study of Parental Views. J Genet Couns. 2015;24(5):851-61.

83. Rice LJ, Gray K. M., Howlin P., Taffe J., Tonge B. J., Einfeld S. L. The developmental trajectory of disruptive behavior in Down syndrome, fragile X syndrome, Prader-Willi syndrome and Williams syndrome. Am J Med Genet C Semin Med Genet. 2015;169(2):182-7.

84. Rice LJ, Gray K. M., Howlin P., Taffe J., Tonge B. J., Einfeld S. L. The Developmental Trajectory of Self-Injurious Behaviours in Individuals with Prader Willi Syndrome, Autism Spectrum Disorder and Intellectual Disability. Diseases. 2016;4(1):06.

85. Roof E, Deal C. L., McCandless S. E., Cowan R. L., Miller J. L., Hamilton J. K., Roeder E. R., McCormack S. E., Roshan Lal T. R., Abdul-Latif H. D., et al. Intranasal Carbetocin Reduces Hyperphagia, Anxiousness, and Distress in Prader-Willi Syndrome: CARE-PWS Phase 3 Trial. Journal of clinical endocrinology and metabolism. 2023;108(7):1696‐708.

86. Royston R, Waite J., Howlin P., Dosse A., Armitage P., Moss J., Oliver C. Cross-syndrome comparison of psychopathological risk factors in williams syndrome, fragile x syndrome and prader-willi syndrome. Journal of Intellectual Disability Research. 2018;62(8):670.

87. Royston R, Oliver C., Howlin P., Dosse A., Armitage P., Moss J., Waite J. The Profiles and Correlates of Psychopathology in Adolescents and Adults with Williams, Fragile X and Prader-Willi Syndromes. J Autism Dev Disord. 2020;50(3):893-903.

88. Rozensztrauch A, Smigiel R. Quality of Life in Children with Prader-Willi Syndrome and the Impact of the Disease on the Functioning of Families. International Journal of Environmental Research and Public Health. 2022;19(23) (no pagination).

89. Rubin DA, Wilson K. S., Castner D. M., Dumont-Driscoll M. C. Changes in Health-Related Outcomes in Youth With Obesity in Response to a Home-Based Parent-Led Physical Activity Program. Journal of Adolescent Health. 2019;65(3):323-30.

90. Rubin DA, Wilson K. S., Tucker J. M., Castner D. M., Dumont-Driscoll M. C., Rose D. J. Improved Motor Proficiency and Quality of Life in Youth With Prader-Willi Syndrome and Obesity 6 Months After Completing a Parent-Led, Game-Based Intervention. Pediatr Exerc Sci. 2021;33(4):177-85.

91. Schofield C, Martin K. S., Choong C., Gibson D., Skoss R., Downs J. Using a trauma informed practice framework to enhance understanding of and identify support strategies for behavioural difficulties in young people with Prader-Willi syndrome. Res Dev Disabil. 2021;110(no pagination).

92. Shivers CM, Leonczyk C. L., Dykens E. M. Life Satisfaction Among Mothers of Individuals with Prader-Willi Syndrome. J Autism Dev Disord. 2016;46(6):2126-37.

93. Shriki-Tal L, Avrahamy H., Pollak Y., Gross-Tsur V., Genstil L., Hirsch H. J., Benarroch F. Psychiatric disorders in a cohort of individuals with Prader-Willi syndrome. Eur Psychiatry. 2017;44:47-52.

94. Strong TV, Miller J. L., McCandless S. E., Gevers E., Yanovski J. A., Matesevac L., Bohonowych J., Ballal S., Yen K., Hirano P., Cowen N. M., Bhatnagar A. Behavioral changes in patients with Prader-Willi syndrome receiving diazoxide choline extended-release tablets compared to the PATH for PWS natural history study. J Neurodev Disord. 2024;16(1):22.

95. Thomson A, Glasson E., Roberts P., Bittles A. "Over time it just becomes easier...": parents of people with Angelman syndrome and Prader-Willi syndrome speak about their carer role. Disabil Rehabil. 2017;39(8):763-70.

96. Trueba-Timmermans DJ, Grootjen L. N., Juriaans A. F., Mahabier E. F., Kerkhof G. F., Rings E. H. H. M., Hokken-Koelega A. C. S. Cognitive function during 3 years of growth hormone in previously growth hormone-treated young adults with Prader-Willi syndrome. Eur. 2023;189(1):132-9.

97. Tvrdik T, Mason D., Dent K. M., Thornton L., Hornton S. N., Viskochil D. H., Stevenson D. A. Stress and coping in parents of children with Prader-Willi syndrome: Assessment of the impact of a structured plan of care. Am J Med Genet A. 2015;167A(5):974-82.

98. Vitale SA. Parent Recommendations for Family Functioning With Prader-Willi Syndrome: A Rare Genetic Cause of Childhood Obesity. J Pediatr Nurs. 2016;31(1):47-54.

99. Wieting J, Eberlein C., Bleich S., Frieling H., Deest M. Behavioural change in Prader-Willi syndrome during COVID-19 pandemic. Journal of Intellectual Disability Research. 2021;65(7):609-16.

100. Wieting J, Jahn K., Buchholz V., Lichtinghagen R., Bleich S., Eberlein C. K., Deest M., Frieling H. Alteration of serum leptin and LEP/LEPR promoter methylation in Prader-Willi syndrome. medRxiv. 2021;15.

101. Wieting J, Jahn K., Buchholz V., Lichtinghagen R., Deest-Gaubatz S., Bleich S., Eberlein C. K., Deest M., Frieling H. Alteration of serum leptin and LEP/LEPR promoter methylation in Prader-Willi syndrome. Psychoneuroendocrinology. 2022;143:105857.

102. Wieting J, Jahn K., Eberlein C. K., Bleich S., Frieling H., Deest M. Hypomethylation of the dopamine transporter (DAT) gene promoter is associated with hyperphagia-related behavior in Prader-Willi syndrome: A case-control study. Behav Brain Res. 2023;450:114494.

103. Wilson KS, Wiersma L. D., Rubin D. A. Quality of life in children with Prader Willi Syndrome: Parent and child reports. Res Dev Disabil. 2016;57:149-57.

104. Wong SB, Wang T. S., Tsai W. H., Tzeng I. S., Tsai L. P. Parenting stress in families of children with Prader-Willi syndrome. Am J Med Genet A. 2021;185(1):83-9.

105. Yamada K, Watanabe M., Suzuki K. Differential volume reductions in the subcortical, limbic, and brainstem structures associated with behavior in Prader-Willi syndrome. Sci. 2022;12(1):4978.

106. Baker EK, Arora S., Amor D. J., Date P., Cross M., O'Brien J., Simons C., Rogers C., Goodall S., Slee J., Cahir C., Godler D. E. The Cost of Raising Individuals with Fragile X or Chromosome 15 Imprinting Disorders in Australia. J Autism Dev Disord. 2023;53(4):1682-92.

107. Bar C, Diene G., Molinas C., Bieth E., Casper C., Tauber M. Early diagnosis and care is achieved but should be improved in infants with Prader-Willi syndrome. Orphanet Journal Of Rare Diseases. 2017;12(1):118.

108. Butler MG, Manzardo A., Strong T. V., Li J. W., Yin D., Meng Q., Silber A., Francis K., Hadker N., Czado S., Yeh M., Miller J. L. Pro22 Cost of Care Analysis for U.S. Patients with Prader-Willi Syndrome (Pws). Value in Health. 2020;23(Supplement 1):S332.

109. Chung AS, Renfree S, Lockwood DB, Karlen J, Belthur M. Syndromic Scoliosis: National Trends in Surgical Management and Inpatient Hospital Outcomes: A 12-Year Analysis. Spine. 2019;44(22):1564-70.

110. Clerc A, Coupaye M., Mosbah H., Pinto G., Laurier V., Mourre F., Merrien C., Diene G., Poitou C., Tauber M. Diabetes Mellitus in Prader-Willi Syndrome: Natural History during the Transition from Childhood to Adulthood in a Cohort of 39 Patients. J. 2021;10(22):15.

111. Elliott JP, Cherpes G., Kamal K., Chopra I., Harrison C., Riedy M., Herk B., McCrossin M., Kalarchian M. Relationship between antipsychotics and weight in patients with Prader-Willi syndrome. Pharmacotherapy. 2015;35(3):260-8.

112. Gul Y, Kapakli H., Aytekin S. E., Guner S. N., Keles S., Zamani A. G., Yildirim M. S., Reisli I. Evaluation of immunological abnormalities in patients with rare syndromes. Central European Journal of Urology. 2022;47(4):299-307.

113. Hughes BM, Holland A., Hodebeck-Stuntebeck N., Garrick L., Goldstone A. P., Lister M., Moore C., Hughes M. Body weight, behaviours of concern, and social contact in adults and adolescents with Prader-Willi syndrome in full-time care services: Findings from pooled international archival data. Orphanet Journal Of Rare Diseases. 2024;19(1):48.

114. Laurier V, Lapeyrade A., Copet P., Demeer G., Silvie M., Bieth E., Coupaye M., Poitou C., Lorenzini F., Labrousse F., Molinas C., Tauber M., Thuilleaux D., Jauregi J. Medical, psychological and social features in a large cohort of adults with Prader-Willi syndrome: Experience from a dedicated centre in France. Journal of Intellectual Disability Research. 2015;59(5):411-21.

115. Luccarelli J. Demographics and medical comorbidities among hospitalized patients with Prader-Willi Syndrome: A National Inpatient Sample analysis. American Journal of Medical Genetics, Part A. 2022;188(10):2899-907.

116. McQuivey KS, Sheridan J. R., Chung A., Mayfield C., Gulbrandsen M., Brinkman J. C., Belthur M. V. Hospital outcomes of scoliosis surgery in children with Prader-Willi Syndrome: comparison with adolescent idiopathic scoliosis. Spine Deformity. 2021;9(6):1641-7.

117. McQuivey KS, Chung A. S., Jones M. R., Makovicka J. L., Christopher Z. K., Brinkman J. C., Belthur M. Hospital outcomes in pediatric patients with Prader-Willi syndrome (PWS) undergoing orthopedic surgery: A 12-year analysis of national trends in surgical management and inpatient hospital outcomes. J Orthop Sci. 2022;27(6):1304-8.

118. Meade C, Martin R., McCrann A., Lyons J., Roche E. Growth and nutritional status in children with Prader Willi Syndrome. Archives of Disease in Childhood. 2019;104(Supplement 3):A117-A8.

119. Pellikaan K, Snijders F, Rosenberg AGW, Davidse K, van den Berg SAA, Visser WE, et al. Thyroid Function in Adults with Prader-Willi Syndrome; a Cohort Study and Literature Review. J. 2021;10(17):25.

120. Pemmasani G, Yandrapalli S. Age-stratified prevalence of relevant comorbidities and etiologies for hospitalizations in Prader-Willi syndrome patients. American Journal of Medical Genetics, Part A. 2021;185(2):600-1.

121. Potluri R, Ranjan S., Khurana R., Lele A. M., Prabhakar V., Bhandari H. Evaluation of average cost-effectiveness ratios of standards of care across different indications. Value in Health. 2015;18(7):A575.

122. Prapasrat C, Onsod P., Korkiatsakul V., Rerkamnuaychoke B., Wattanasirichaigoon D., Chareonsirisuthigul T. The Utilization of MS-MLPA as the First-Line Test for the Diagnosis of Prader-Willi Syndrome in Thai Patients. J Pediatr Genet. 2021;12(4):273-9.

123. Sequeira AR, Mentzakis E., Archangelidi O., Paolucci F. The economic and health impact of rare diseases: A meta-analysis. Health Policy and Technology. 2021;10(1):32-44.

124. Shepherd S, Saraff V., Shaw N., Banerjee I., Patel L. Growth hormone prescribing patterns in the UK, 2013-2016. Archives of Disease in Childhood. 2019;104(6):583-7.

125. Shoffstall AJ, Gaebler J. A., Kreher N. C., Niecko T., Douglas D., Strong T. V., Miller J. L., Stafford D. E., Butler M. G. The High Direct Medical Costs of Prader-Willi Syndrome. J Pediatr. 2016;175:137-43.

126. Van Bosse HJP, Gantz M. G., Ong K. L., Cox J. B. Comparison of Hip and Knee Arthroplasty Rates of Individuals With and Without Prader-Willi Syndrome. Journal of Pediatric Orthopaedics. 2020;40(5):E362-E6.

127. Yang L, Zou C. Perinatal features of prader-willi syndrome: A Chinese cohort. Hormone Research in Paediatrics. 2019;91(Supplement 1):300.

128. Yang L, Zhou Q., Ma B., Mao S., Dai Y., Zhu M., Zou C. Perinatal features of Prader-Willi syndrome: a Chinese cohort of 134 patients. Orphanet Journal Of Rare Diseases. 2020;15(1):24.
